# ViBra: Configuration Interaction for Anharmonic Vibrational Spectroscopy and Quantum-Sampled Configuration Spaces

**DOI:** 10.1021/acs.jctc.6c01564

**Published:** 2026-09-28

**Authors:** Raphael F. Ligorio, Marco Antonio Barroca, Alan Duriez, Mathias B. Steiner

**Affiliations:** † 74350 Centro Brasileiro de Pesquisas Físicas (CBPF), Rio de Janeiro, Rio de Janeiro 22290-180, Brasil; ‡ 840263 Instituto Kunumi, Belo Horizonte, Minas Gerais 30130-138, Brasil

## Abstract

Quantum-centric workflows are a promising route to improving the accuracy of property predictions in computational chemistry and materials science. By integrating quantum sampling algorithms with classical solvers, electronic structure calculations have recently demonstrated their potential even on noisy intermediate-scale quantum devices. In principle, the method of Vibrational Configuration Interaction (VCI) is suitable for integration with quantum sampling algorithms as well. However, demonstrations of computational workflows for quantum-centric, vibrational property predictions are still lacking. Here, we introduce a methodology for performing anharmonic vibrational structure calculations that can be deployed in a hybrid, quantum-classical mode. Starting from a quartic force field, the approach combines a Vibrational Self-Consistent Field (VSCF) with VCI in Full, Selected (S-VCI), or Symmetry-Adapted (SA-VCI) modes. In S-VCI, an Epstein–Nesbet perturbative screening significantly reduces the configuration space while retaining high predictive accuracy. A state-list input enables the integration of externally generated vibrational configurations as a seed space. As a proof-of-concept, we demonstrate a hybrid, quantum-classical computational workflow in which a quantum sampling algorithm provides the seed. Our vibrational wave function analysis package ViBra, equipped with a graphical interface, is available at https://github.com/raphafe96/ViBra.

## Graphical abstract

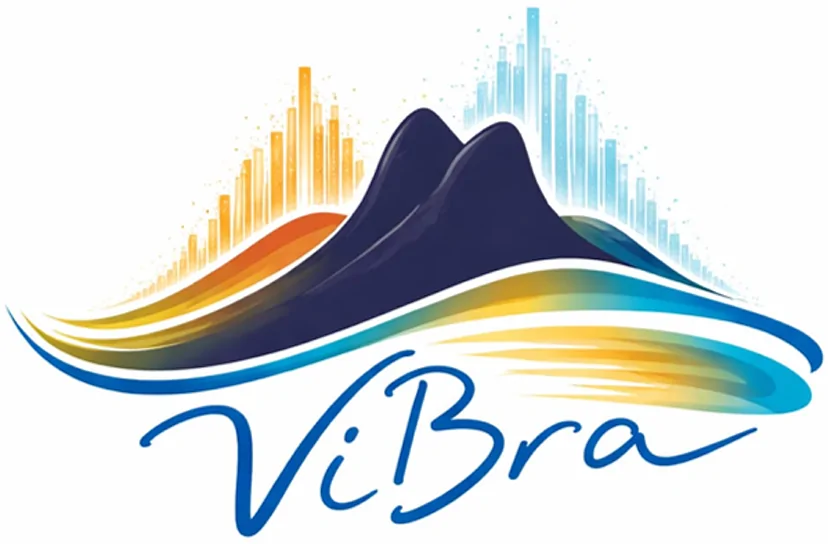

## 1 Introduction

Vibrational spectroscopy provides a direct window into the molecular structure and dynamics. The positions and intensities of fundamental, overtone, and combination bands encode information about bond strengths, mode couplings, and underlying potential energy surfaces (PESs). Accurate theoretical prediction of these observables therefore constitutes a stringent test of electronic structure methods and is indispensable for the assignment of the experimental spectra.

Within the Born–Oppenheimer approximation, the nuclear vibrational problem can be formulated as the solution of the vibrational Schrödinger equation in mass-weighted normal coordinates **Q** = (*Q*_1_, ..., *Q*_*M*_), where M = 3*N*_at_ – 6 or M = 3*N*_at_ – 5 for linear molecules is the number of vibrational degrees of freedom. The simplest harmonic approach approximation uses a quadratic PES and diagonalizes the mass-weighted Hessian to yield independent normal modes. However, this approximation fails to reproduce experimental fundamental frequencies, cannot describe overtones and combination bands, and gives vanishing intensities for these transitions within the double-harmonic (mechanical and electrical) approximation. The accurate treatment of mechanical anharmonicity requires the inclusion of at least cubic and quartic terms in the PES expansion.^1,2^

Two broad classes of methods have been developed for the anharmonic vibrational problem. **Perturbative** approaches, exemplified by second-order vibrational perturbation theory (VPT2),^1,3^ are computationally efficient and have been widely deployed in quantum chemistry codes such as Gaussian^4^ and ORCA.^5,6^ However, VPT2 breaks down in the presence of Fermi and Darling–Dennison resonances, requiring ad hoc treatments that can compromise accuracy.

**Variational** methods, by contrast, are systematically improvable and robust in the presence of resonances. The vibrational self-consistent field (VSCF) approach, introduced independently by Bowman^7^ and by Gerber and Ratner,^8^ provides a mean-field description in which each normal mode evolves in the average potential of all other modes. To recover the correlation missed by the mean-field treatment, VSCF is typically followed by vibrational configuration interaction (VCI), where the wave function is expanded in a basis of Hartree products of VSCF-optimized one-mode functions (modals) and the Hamiltonian is diagonalized in this space.^2,9,10^

Over the past three decades, several powerful VCI implementations have been developed. The **MULTIMODE** code, developed by Bowman and coworkers,^10^ uses an *n*-mode representation of the potential in normal coordinates with a hierarchical coupling scheme and has been applied to molecules with up to dozens of atoms. The **MOLPRO** package features state-specific configuration-selective VCI (cs-VCI) algorithms pioneered by Rauhut and coworkers,^11,12^ which use perturbative estimates to efficiently screen the correlation space. More recently, Mathea and collaborators have introduced advanced screening techniques and efficient handling of rovibrational angular-momentum terms that substantially accelerate these calculations.^13,14^

The **CRYSTAL** program, developed by Erba, Maul, Dovesi, and coworkers, has extended VSCF and VCI methods to periodic systems (solids), implementing a fully automated scheme for constructing the anharmonic PES from density functional theory (DFT) calculations and computing anharmonic vibrational states of solids.^15,16^ A subsequent extension by Carbonnière et al. additionally enabled the calculation of anharmonic infrared and Raman intensities for periodic systems.^17^ Since then, anharmonic vibrations in materials have been extensively explored.^18–21^

In the open-source domain, the **PyVCI** package by Sibaev and Crittenden^22^ provides a flexible Python-based VCI implementation with computationally intensive routines written in C via Cython. The **DVCI** (Dual Vibration Configuration Interaction) method by Garnier et al.^23^ employs an efficient factorization of the Hamiltonian to target specific spectral transitions with minimal memory usage, while the **A-VCI** (Adaptive VCI) algorithm^24,25^ iteratively constructs nested bases guided by a posteriori error estimates.

Because the size of the VCI matrix scales combinatorially with the number of modes, selecting only the most relevant configurations rather than expanding the space exhaustively is essential for practical calculations. Perturbation-theory screening offers a systematic way to identify strongly coupled basis states to a small reference, significantly compressing the problem while retaining the precision.^26^ This philosophy motivates the vibrational heat-bath configuration interaction (VHCI) method, introduced independently by Fetherolf and Berkelbach^27^ and by Bhatty and Brorsen.^28^ By borrowing the heat-bath selection idea from electronic structure theory, VHCI focuses the variational treatment on a compact, dynamically chosen set of configurations. More recently, Tran et al.^29^ expanded the approach by introducing VSCF-optimized modals and a semistochastic perturbation theory framework, which together accelerate convergence and extend its applicability.

Despite this wealth of methodology, several challenges remain: the exponential scaling of the VCI basis with *M*, the accurate treatment of transition intensities beyond the linear dipole surface, and the need for user-friendly software that bridges computation and visualization. We therefore designed ViBra to address these challenges through a combination of:

- **Selected CI (S-VCI):** an Epstein–Nesbet perturbation-theory-driven configuration selection that significantly reduces the VCI space while preserving accuracy for low-lying states (Section 2.5);
- **Symmetry-adapted VCI:** exact block-diagonalization of the Hamiltonian for molecules in Abelian point groups with one-dimensional irreducible representations (*C*_1_, *C*_*s*_, *C*_*i*_, *C*_2_, *C*_2*h*_, *C*_2*v*_, *D*_2_, *D*_2*h*_), providing irrep labels and reducing diagonalization cost by up to a factor of $n_{\Gamma }^{2}$ (Section 2.6), where *n*_Γ_ is the number of symmetry elements of a given Abelian point group;
- **Treatment of electrical anharmonicity:** all VCI-level transition dipoles include both first- and second-order dipole-moment derivatives, essential for overtone and combination band intensities and for the correct redistribution of oscillator strength through Fermi resonances (Section 2.7);
- **Graphical interface and interactive viewer:** a Python/CustomTkinter GUI for input preparation and job execution and a Matplotlib-based viewer with temperature-dependent spectra, experimental overlay, baseline correction, and 3D normal-mode animation via 3Dmol.js (Section 3.1).

A further motivation for the development of ViBra is the growing interest in quantum algorithms for the vibrational structure and spectroscopy. In quantum chemistry and materials science, recent quantum-centric workflows such as sample-based quantum diagonalization (SQD) and sample-based Krylov quantum diagonalization (SKQD) have highlighted a practical near-term paradigm in which quantum processors are used as sampling devices, while compact Hamiltonian construction, diagonalization, and observable evaluation are performed classically.^30,31^ More broadly, quantum-centric supercomputing has been proposed as a route for integrating quantum processors with high-performance classical resources in materials science workflows.^32^ In contrast to electronic-structure applications, however, quantum algorithms for anharmonic vibrational dynamics remain comparatively underdeveloped, despite recent progress in quantum algorithms for vibronic spectra, vibrational structure calculations, and Trotterized simulations of vibrational Hamiltonians.^33–35^

The selected-VCI infrastructure implemented in ViBra is well-aligned with this sampling-based perspective because it can serve as the classical VSCI engine after a quantum-generated seed space has been obtained. A quantum sampling algorithm proposes a compact set of vibrational configurations, while ViBra can either construct and diagonalize the Hamiltonian directly in that sampled space or enlarge it through an EN-PT2-guided selection before variational rediagonalization. In both cases, ViBra evaluates transition observables and generates anharmonic vibrational spectra from the final configuration space. Thus, ViBra provides both the projected-VCI postprocessing layer and an optional classical refinement stage for quantum-seeded vibrational configuration spaces.

## 2 Theoretical Framework

The potential energy surface (PES) governing the nuclear motion can be represented in various forms, including numerical grids or analytical expansions. In ViBra, we have opted for a Taylor expansion of the PES in terms of the normal coordinates, truncated at the fourth order.^16^ This quartic force field representation provides a compact, analytically tractable form that enables efficient evaluation of all required matrix elements in closed form since low-order polynomial terms in *Q*_i_ have simple analytic matrix elements in the harmonic-oscillator basis. Furthermore, it allows for a clean separation of the potential into harmonic, diagonal anharmonic, and coupling contributions, which are essential for the VSCF and VCI formulations described below. Grid-based representations such as the discrete variable representation (DVR),^36^ while more flexible and not restricted to a low-order polynomial form, require the PES to be evaluated on a dense multidimensional grid whose size grows exponentially with the number of modes, and they do not offer a natural analytic separation into diagonal and coupling terms; this makes them less suitable for the sparsity-driven algorithms (Section 5) that ViBra relies on.

### 2.1 Vibrational Hamiltonian and Quartic Force Field

For a nonlinear molecule with *N*_at_ atoms, mass-weighted normal coordinates **Q** = (*Q*_1_, ..., *Q*_*M*_) are obtained by diagonalizing the mass-weighted Cartesian Hessian, where M = 3*N*_at_ – 6 is the number of vibrational degrees of freedom. Neglecting rotation–vibration coupling (*J* = 0) and the Watson correction terms that are small for semirigid molecules, the vibrational Hamiltonian in atomic units reads

1 $${Ĥ}_{\mathrm{vib}}=-\frac{1}{2}\overset{M}{\underset{i=1}{\sum }}\frac{\partial^{2}}{\partial Q_{i}^{2}}+V\left(Q\right)$$

The potential energy surface *V*(**Q**) is expanded in a Taylor series about the equilibrium geometry, here up to fourth order:

2 $$V\left(Q\right)=\frac{1}{2}\overset{M}{\underset{i=1}{\sum }}\omega_{i}^{2}Q_{i}^{2}+\frac{1}{6}\overset{M}{\underset{i=1}{\sum }}\overset{M}{\underset{j=1}{\sum }}\overset{M}{\underset{k=1}{\sum }}\phi_{ijk}Q_{i}Q_{j}Q_{k}+\frac{1}{24}\overset{M}{\underset{i=1}{\sum }}\overset{M}{\underset{j=1}{\sum }}\overset{M}{\underset{k=1}{\sum }}\overset{M}{\underset{l=1}{\sum }}\phi_{ijkl}Q_{i}Q_{j}Q_{k}Q_{l}$$

The tensors are totally symmetric under index permutation; only the upper-triangular components are stored, and each distinct term carries a permutational degeneracy factor. For cubic terms with *i* ≤ *j* ≤ *k*, the weight *g* is 1 (all indices equal), 3 (two equal), or 6 (all distinct). For quartic terms *i* ≤ *j* ≤ *k* ≤ *l*, the weight is 1 (all equal), 4 (three equal), 6 (two doubles), 12 (one double, two singles), or 24 (all distinct). The code stores the effective potential in terms of these weighted coefficients as

3 $$V\left(Q\right)=\underset{i}{\sum }\left(\frac{1}{2}\omega_{i}^{2}Q_{i}^{2}+{\mathrm{\phi ̃}}_{iii}Q_{i}^{3}+{\mathrm{\phi ̃}}_{iiii}Q_{i}^{4}\right)+\underset{i<j\leq k}{\sum }{\mathrm{\phi ̃}}_{ijk}Q_{i}Q_{j}Q_{k}+\underset{i<j\leq k\leq l}{\sum }{\mathrm{\phi ̃}}_{ijkl}Q_{i}Q_{j}Q_{k}Q_{l}$$

The coefficients ϕ̃_···_ incorporate the original force constants and the appropriate degeneracy factors. Internally, a separate diagonal potential $V_{i}^{\mathrm{diag}}\left(Q_{i}\right)=\frac{1}{2}\omega_{i}^{2}Q_{i}^{2}+{\mathrm{\phi ̃}}_{iii}Q_{i}^{3}+{\mathrm{\phi ̃}}_{iiii}Q_{i}^{4}$ is identified for each mode, and the coupling potential *V*_*c*_ groups all terms involving at least two distinct mode indices.

It is important to note here that the harmonic frequencies ω_*i*_ and the cubic and quartic force constants ϕ_*ijk*_ and ϕ_*ijkl*_ are provided exclusively in the ORCA VPT2 output format (.vpt2).^5,6^ This format was chosen because it provides all necessary information, including harmonic frequencies, cubic and quartic force constants with their permutational degeneracy factors, normal-coordinate transformation matrices, and first and second dipole derivatives, in a self-contained, well-documented text structure. Notably, ORCA is a free quantum chemistry package, making the entire computational workflow from force constant evaluation to anharmonic vibrational analysis fully freely accessible. Users of other quantum chemistry packages can convert their outputs to the equivalent format using as reference the ORCA output files provided in Supporting Information.

### 2.2 One-Mode Basis: Harmonic Oscillator

The primitive one-mode basis consists of harmonic oscillator eigenfunctions ϕ_*n*_(*Q*_*i*_) with frequency ω_*i*_. Their matrix elements

4 $$m_{p}^{\left(\mu ,\nu \right)}\equiv \left\langle \phi_{\mu }\right|Q_{i}^{p}\left|\phi_{\nu }\right\rangle$$

for *p* = 0, ..., 4 are evaluated analytically using ladder-operator algebra. The kinetic energy matrix elements are computed from the second-derivative operator:

5 $$\left\langle \phi_{\mu }\right|{\mathrm{T̂}}_{i}\left|\phi_{\nu }\right\rangle =-\frac{1}{2}\left\langle \phi_{\mu }\right|\frac{\partial^{2}}{\partial Q_{i}^{2}}\left|\phi_{\nu }\right\rangle$$

A fundamental selection rule governs these integrals: $\left\langle \phi_{\mu }\right|Q_{i}^{p}\left|\phi_{\nu }\right\rangle =0$ whenever |μ – ν| > *p*. All required matrix elements up to *p* = 4 are precomputed by the compute_integrals module and stored in memory for efficient look-up.

### 2.3 VSCF Method and Modal Basis

The VSCF wave function is the Hartree product

6 $$\Psi_{n}\left(Q\right)=\overset{M}{\underset{i=1}{\prod }}\varphi_{n_{i}}^{\left(i\right)}\left(Q_{i}\right)$$

where **n** = (*n*_1_, ..., *n*_*M*_) specifies the quantum state of each mode. Each one-mode function (modal) is expanded in the HO basis:

7 $$\varphi_{n_{i}}^{\left(i\right)}\left(Q_{i}\right)=\overset{N_{\exp}}{\underset{\mu =1}{\sum }}C_{\mu ,n_{i}}^{\left(i\right)}\phi_{\mu -1}\left(Q_{i}\right)$$

Here, *N*_exp_ is the number of HO basis functions per mode (user-supplied keyword NEXPAN; typical values range from 8 to 16).

Requiring the energy functional *E*_**n**_ = ⟨Ψ_**n**_|*Ĥ*_vib_|Ψ_**n**_⟩ to be stationary with respect to variations of each modal under the constraint $\left\langle \varphi_{n_{i}}^{\left(i\right)}\right|\varphi_{n_{i}}^{\left(i\right)}\rangle =1$ yields *M* coupled effective one-mode equations:

8 $${ĥ}_{i}^{\mathrm{eff}}\varphi_{n_{i}}^{\left(i\right)}\left(Q_{i}\right)=\varepsilon_{n_{i}}^{\left(i\right)}\varphi_{n_{i}}^{\left(i\right)}\left(Q_{i}\right)$$

where $\varepsilon_{n_{i}}^{\left(i\right)}$ acts as the Lagrange multiplier enforcing normalization and has the physical interpretation of a modal energy. The effective Hamiltonian

9 $${ĥ}_{i}^{\mathrm{eff}}={\mathrm{T̂}}_{i}+V_{i}^{\mathrm{diag}}\left(Q_{i}\right)+{\langle V_{c}\rangle }_{j\neq i}$$

contains the kinetic energy of mode *i*, its diagonal potential, and the mean-field average of the coupling potential over all other modes:

10 $$\left\langle V_{c}\rangle_{j\neq i}=\left\langle \underset{j\neq i}{\prod }\varphi_{n_{j}}^{\left(j\right)}\right|V_{c}\left(Q\right)\right|\underset{j\neq i}{\prod }\varphi_{n_{j}}^{\left(j\right)}\rangle$$

Because *V*_*c*_ is a polynomial of degree four in the normal coordinates, the mean-field average is itself a polynomial in *Q*_*i*_ so that

11 $${ĥ}_{i}^{\mathrm{eff}}={\mathrm{T̂}}_{i}+\overset{4}{\underset{p=0}{\sum }}X_{i}^{\left(p\right)}Q_{i}^{p}$$

The coefficients $X_{i}^{\left(p\right)}$ are real numbers that depend on the force constants and on the current expectation values $\left\langle \varphi_{n_{j}}^{\left(j\right)}\right|Q_{j}^{c}\left|\varphi_{n_{j}}^{\left(j\right)}\right\rangle$ of all modes *j* ≠ *i*. They are updated at each self-consistent iteration.

Substituting the modal expansion ([Disp-formula eq7]) into ([Disp-formula eq8]) and projecting onto ⟨ϕ_ν–1_| yield an *N*_exp_ × *N*_exp_ real symmetric matrix eigenvalue problem:

12 $$\overset{N_{\exp}}{\underset{\mu =1}{\sum }}H_{\nu \mu }^{\left(i\right)}C_{\mu ,n_{i}}^{\left(i\right)}=\varepsilon_{n_{i}}^{\left(i\right)}C_{\nu ,n_{i}}^{\left(i\right)}$$

with matrix elements

13 $$H_{\nu \mu }^{\left(i\right)}=\left\langle \phi_{\nu -1}\right|{\mathrm{T̂}}_{i}\left|\phi_{\mu -1}\right\rangle +\overset{4}{\underset{p=0}{\sum }}X_{i}^{\left(p\right)}\left\langle \phi_{\nu -1}\right|Q_{i}^{p}\left|\phi_{\mu -1}\right\rangle$$

The *n*_*i*_-th eigenvector gives the updated coefficients $C_{\mu ,n_{i}}^{\left(i\right)}$ . The VSCF equations are solved iteratively (Algorithm 1) until the energy change between successive iterations falls below 10^–CVGSCF^ cm^–1^. The ground-state modals are stored for use as the VCI one-mode basis.

Because each coupling term in *V*_*c*_ is counted in the effective Hamiltonian of every mode (either as an operator or as a constant shift $X_{i}^{\left(0\right)}$ ), the sum of modal energies $\underset{i}{\sum }\varepsilon_{n_{i}}^{\left(i\right)}$ overcounts the coupling energy. The correct VSCF total energy is therefore

14 $$E_{\mathrm{VSCF}}=\overset{M}{\underset{i=1}{\sum }}\varepsilon_{n_{i}}^{\left(i\right)}-\left(M-1\right){\mathrm{V̅}}_{c}$$

where the correlation potential

15 $${\mathrm{V̅}}_{c}=\left\langle \overset{M}{\underset{i=1}{\prod }}\varphi_{n_{i}}^{\left(i\right)}\right|V_{c}\left(Q\right)\left|\overset{M}{\underset{i=1}{\prod }}\varphi_{n_{i}}^{\left(i\right)}\right\rangle$$

is the fully averaged coupling potential. This correction ensures that each coupling term appears exactly once in the total energy.
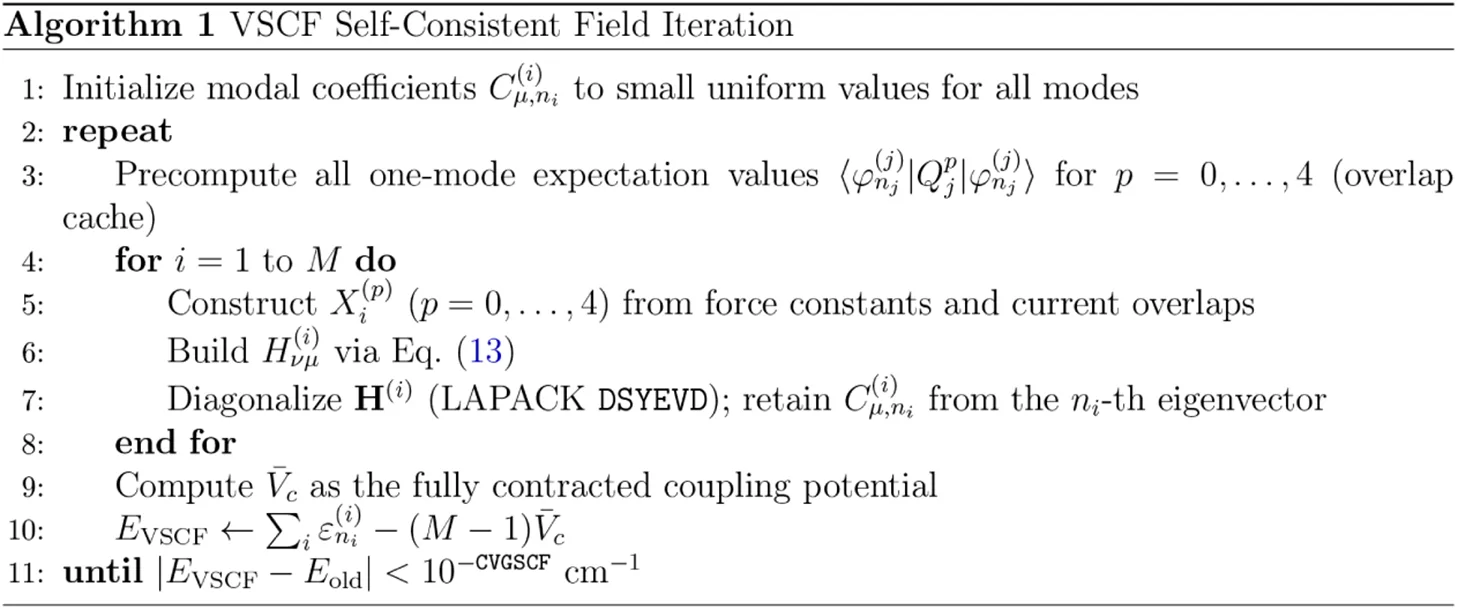


### 2.4 VCI Expansion and Precomputed Modal Integrals

The VCI expansion can be built using either the primitive harmonic oscillator basis or the ground-state VSCF modals. The VSCF modals yield faster variational convergence of the VCI energies with respect to increasing the maximum total quanta, as they already account for the average effect of anharmonic couplings. This is achieved without additional computational overhead since all required modal integrals are precomputed and stored (see Section 4). The VSCF-based VCI approach is therefore recommended for routine anharmonic vibrational calculations and thus employed in ViBra. As a result, the wave function is a linear combination of Hartree products built from the *ground-state* VSCF modals:

16 $$\left|\Psi_{I}\right\rangle =\underset{n\in S}{\sum }c_{n}^{\left(I\right)}\left|\Phi_{n}\right\rangle ,,\left|\Phi_{n}\right\rangle =\overset{M}{\underset{i=1}{\prod }}\left|\varphi_{n_{i}}^{\left(i\right)}\right\rangle$$

The configuration space S is truncated by a maximum total quantum number *N*_*q*_ (keyword NQUANT) such that $\overset{M}{\underset{i=1}{\sum }}n_{i}\leq N_{q}$ . Using a single fixed set of ground-state modals ensures orthonormality of the configuration basis. A critical component enabling efficient VCI is the precomputed modal integral table:

17 $$I_{i}\left(v,v^{\prime };p\right)\equiv \left\langle \varphi_{v}^{\left(i\right)}\right|Q_{i}^{p}\left|\varphi_{v^{\prime }}^{\left(i\right)}\right\rangle =\overset{N_{\exp}}{\underset{\mu ,\nu =1}{\sum }}C_{\mu ,v}^{\left(i\right)}C_{\nu ,v^{\prime }}^{\left(i\right)}\left\langle \phi_{\mu -1}\right|Q_{i}^{p}\left|\phi_{\nu -1}\right\rangle$$

with *p* = 0, ..., 5 (where *p* = 5 stores kinetic energy matrix elements in the modal basis). The table is symmetric in its first two arguments and occupies *M* × (*N*_*q*_ + 1)^2^ × 6 double-precision entries—typically a few tens of megabytes. Once computed, every one-mode integral required for the VCI Hamiltonian construction becomes an *O*(1) look-up, eliminating the need for the $O\left(N_{\exp}^{2}\right)$ double sum otherwise required for each evaluation.

The Hamiltonian matrix element *H*_**mn**_ ≡ ⟨Φ_**m**_|*Ĥ*_vib_|Φ_**n**_⟩ is assembled from three types of contribution:

**One-body terms** collect all diagonal anharmonic corrections for each mode *i*:

18 $$H^{\left(1\right)}=\overset{M}{\underset{i=1}{\sum }}\left[I_{i}\left(m_{i},n_{i};5\right)+{\mathrm{\phi ̃}}_{iiii}I_{i}\left(m_{i},n_{i};4\right)+{\mathrm{\phi ̃}}_{iii}I_{i}\left(m_{i},n_{i};3\right)+\frac{\omega_{i}}{2}I_{i}\left(m_{i},n_{i};2\right)\right]S_{\mathrm{all}\backslash i}$$

**Cubic coupling terms** with unique modes {*s*_1_, *s*_2_, *s*_3_} and multiplicities {*p*_1_, *p*_2_, *p*_3_} contribute

19 $$H^{\left(3,\mathrm{term}\right)}={\mathrm{\phi ̃}}_{\mathrm{term}}\overset{3}{\underset{r=1}{\prod }}I_{s_{r}}\left(m_{s_{r}},n_{s_{r}};p_{r}\right)S_{\mathrm{all}\backslash \{s_{1},s_{2},s_{3}\}}$$

**Quartic coupling terms** are treated analogously, with up to four unique modes.

In the above, *S*_all\*S*_ denotes the product of spectator-mode overlaps:

20 $$S_{\mathrm{all}\backslash S}=\underset{k\notin S}{\prod }I_{k}\left(m_{k},n_{k};0\right)$$

A fundamental selection rule emerges: because $I_{k}\left(m_{k},n_{k};0\right)=\delta_{m_{k},n_{k}}$ (modal orthonormality), a potential term can couple configurations **m** and **n** only if it involves *all* modes in which the two configurations differ. Consequently, two VCI configurations can be coupled only if they differ in at most four modes (the maximum number of distinct indices in a quartic term). This sparsity is exploited through a precomputed pair list and an inverted potential index.

The Hamiltonian is diagonalized using LAPACK routines: DSYEVD (divide-and-conquer) for full eigenvalue spectra and DSYEVR (relatively robust representations) for partial spectra when only the lowest *N*_state_ eigenvalues are required.

### 2.5 Selected Vibrational CI (S-VCI)

Full VCI becomes prohibitive for large *M* and *N*_*q*_ since $\left|S\right|∼\left(\begin{array}{l} M+N_{q}\\ N_{q} \end{array}\right)$ . ViBra’s SCI algorithm addresses this by constructing a compact active space through Epstein–Nesbet perturbation theory^37,38^ screening (EN-PT).

#### 2.5.1 Reference Space

The reference space $S_{\mathrm{ref}}$ is set by the user through the MAXSCI keyword,
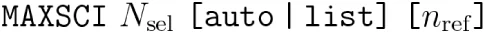


where *N*_sel_ is the number of external configurations retained per reference state (Section 2.5.3); the second argument selects how the reference space is built; and the third, optional, argument sets the truncation order *n*_ref_, used only when the auto option is chosen.

In auto mode, the reference space comprises all configurations whose total excitation does not exceed *n*_ref_,

21 $$S_{\mathrm{ref}}=\left\{n\in S:\underset{i}{\sum }n_{i}\leq n_{\mathrm{ref}}\right\}$$

For *n*_ref_ = 2 (ground state, one-mode singles *n*_*i*_ = 1, and doubles *n*_*i*_ = 2 or *n*_*i*_ = *n*_*j*_ = 1 pairs), this has dimension $1+2M+\left(\begin{array}{l} M\\ 2 \end{array}\right)$ , linear in *M* for the singles and quadratic for the doubles; larger values of *n*_ref_ extend the reference to include triples, quadruples, and so on, at the cost of a larger reference Hamiltonian.

In list mode, the reference space is instead supplied explicitly by the user through a plain-text file, list_states.txt, whose first line gives the number of reference configurations, followed by one configuration (a length-*M* vector of mode quanta, *n*_*i*_) per line. This allows the user to target configurations of specific chemical or spectroscopic interest, independent of any excitation-order truncation. In either case, the Hamiltonian in the resulting reference space $S_{\mathrm{ref}}$ is built and diagonalized exactly, yielding reference energies $E_{I}^{\left(0\right)}$ and eigenvectors $\left|\Psi_{I}^{\left(0\right)}\right\rangle =\underset{n\in S_{\mathrm{ref}}}{\sum }c_{n}^{\left(I\right)}\left|\Phi_{n}\right\rangle$ , for *I* = 1, ..., *N*_state_, where *N*_state_ here is the number of vibrational reference states. Notably, for both auto and list modes, no iterative cycles are performed automatically; that is, the final active space is not used as the reference space for a subsequent selected VCI calculation. If such an iterative procedure is desired, the user can instead provide the active space resulting from a previous S-VCI run as a new list.

#### 2.5.2 Epstein–Nesbet PT2 Screening

For each external configuration $\left|\Phi_{\alpha }\right\rangle \notin S_{\mathrm{ref}}$ and each reference state *I* to be converged, the Epstein–Nesbet second-order energy correction is computed:

22 $$e_{\alpha }^{\left(I\right)}=\frac{{|\sum_{n\in S_{\mathrm{ref}}}c_{n}^{\left(I\right)}H_{\alpha n}|}^{2}}{E_{I}^{\left(0\right)}-H_{\alpha \alpha }}$$

The denominator *H*_*αα*_ is the diagonal Hamiltonian element of the external configuration itself, *H*_*αα*_ = ⟨Φ_α_|*H*|Φ_α_⟩. This is the defining feature of the Epstein–Nesbet partitioning of the Hamiltonian: rather than approximating a configuration’s zeroth-order energy as a sum of independent-mode (harmonic) energies, as would be done in a Møller–Plesset-type partitioning, the exact diagonal matrix element—already including diagonal anharmonic corrections—is used directly. This makes the energy denominator in eq 22 a tighter and more physically faithful estimate for each configuration, at the cost of no additional computation beyond evaluating *H*_*αα*_. The numerator in eq 22 instead couples |Φ_α_⟩ to every reference configuration **n** that differs by at most four quanta; this coupling is evaluated efficiently using the inverted potential index (Section 4). The external states are automatically generated based on the maximum number of quanta specified in the input file. When the user sets the maximum number of quanta, the program checks whether any states in the reference contain more quanta than the specified value. If such states are found, the program stops and displays a warning asking the user to increase the maximum number of quanta.

#### 2.5.3 Top-*N* Selection and Active Space Construction

For each reference state *I*, the *N*_sel_ external configurations with the largest $\left|e_{\alpha }^{\left(I\right)}\right|$ are retained, where *N*_sel_ is the first argument of the MAXSCI keyword. A sorted insertion procedure maintains the top-*N* candidates for each state as the external space is scanned once, avoiding a full sort of all PT2 contributions. The final active space is the union

23 $$S_{\mathrm{active}}=S_{\mathrm{ref}}\cup \overset{N_{\mathrm{state}}}{\underset{I=1}{\cup }}S_{\mathrm{sel}}^{\left(I\right)}$$

Because a single external configuration may be selected by multiple reference states, duplicate insertions are automatically avoided when the union is formed. The full VCI Hamiltonian is then rebuilt in the final active space and diagonalized using the same optimized kernel used for the reference space and for the conventional full-VCI calculation.

For example, for the ethylene molecule, with 12 vibrational modes, a choice of 6 quanta, using (MAXSCI 100 auto d) yields an active space corresponding to only 32% of the full VCI dimension, while the lowest eigenvalues agree with the full VCI results within 1–5 cm^–1^. S-VCI is activated automatically when MAXSCI *N*_sel_ > 0 and no molecular symmetry is specified (i.e., PGROUP is C1 or absent). If symmetry is active, the code defaults to the symmetry-adapted full VCI.

### 2.6 Symmetry-Adapted VCI (SA-VCI)

#### 2.6.1 Motivation and Scope

The vibrational Hamiltonian *Ĥ*_vib_ is invariant under every symmetry operation of the molecular point group $G:\left[{Ĥ}_{\mathrm{vib}},\mathrm{R̂}\right]=0$ for all R̂∈G . Consequently, its matrix representation in a symmetry-adapted basis is block-diagonal: matrix elements between configurations of different irreducible representations (irreps) are identically zero.

ViBra exploits this for the eight Abelian point groups with exclusively one-dimensional irreps: *C*_1_, *C*_*s*_, *C*_*i*_, *C*_2_, *C*_2*h*_, *C*_2*v*_, *D*_2_, *D*_2*h*_. In these groups, all characters are ±1, and the irrep of a Hartree product factorizes as a direct product of one-mode irreps.

#### 2.6.2 Determination of Normal-Mode Irreps

The irrep of each normal mode *k* is determined by projecting its Cartesian displacement vector **L** ^(*k*)^ (a 3*N*_at_-dimensional vector) onto the symmetry operations in the principal-axis frame (PAF). The character of mode *k* under operation *R̂* is

24 $$\chi^{\left(k\right)}\left(\mathrm{R̂}\right)=\frac{1}{{∥L^{\left(k\right)}∥}^{2}}\overset{N_{\mathrm{at}}}{\underset{a=1}{\sum }}\underset{\alpha ,\beta }{\sum }L_{\alpha ,\mathrm{R̂}(a)}^{\left(k\right)}R_{\alpha \beta }L_{\beta ,a}^{\left(k\right)}$$

Here, **R** is the 3 × 3 rotation/reflection matrix of the operation, *R̂*(*a*) is the atom to which atom *a* is mapped, and the double sum runs over all three Cartesian components and all atoms. The sum includes both “diagonal” contributions (atoms mapped to themselves) and “off-diagonal” (permuted) ones, both of which are essential for obtaining the correct ±1 character when the mode displacement is concentrated on permuted atoms.

The PAF is obtained by translating the molecule to its center of mass, computing the inertia tensor *I*_*pq*_ = ∑_*a*_ *m*_*a*_(δ_*pq*_|**r**_*a*_|^2^ – *r*_*a*,*p*_ *r*_*a*,*q*_), and diagonalizing it (LAPACK dsyev). The rotated coordinates and displacement vectors are used in eq 24.

The irrep Γ_*k*_ of mode *k* is identified by the minimal *L* ^1^ distance between the computed character vector **χ** ^(*k*)^ and the rows of the point-group character table.

#### 2.6.3 Configuration Irreps and Block Diagonalization

The VSCF modals inherit the parity structure of the HO basis: $\mathrm{R̂}\varphi_{n_{i}}^{\left(i\right)}\left(Q_{i}\right)={[\chi (\Gamma_{i},\mathrm{R̂})]}^{n_{i}}\varphi_{n_{i}}^{\left(i\right)}\left(Q_{i}\right)$ . Therefore, the contribution of mode *i* to the irrep of a configuration is Γ_*i*_ when *n*_*i*_ is odd and Γ_1_ (the totally symmetric irrep) when *n*_*i*_ is even. The irrep of a configuration is the direct product of all contributing odd-quanta mode irreps:

25 $$\Gamma \left(\left|\Phi_{n}\right\rangle \right)=\overset{M}{\underset{\begin{array}{c} i=1\\ n_{i}\mathrm{odd} \end{array}}{⊗}}\Gamma \left(Q_{i}\right)$$

The configuration space partitions into *n*_Γ_ blocks: $S=\underset{\Gamma }{\cup }B_{\Gamma }$ . Each block is diagonalized independently, reducing the cost from $O\left({|S|}^{3}\right)$ to $\underset{\Gamma }{\sum }O\left({|B_{\Gamma }|}^{3}\right)$ . No approximation is introduced: the symmetry-adapted VCI is exactly equivalent to the full VCI for the correct group assignment.

#### 2.6.4 Diagnostic Output and User Verification

The user specifies the point group via the PGROUP keyword. Because ViBra cannot autodetect the true symmetry, extensive diagnostic output is printed to stdout and debug_sym.txt: PAF coordinates, atom permutation tables, per-atom contributions to each mode character, and the assigned irrep. A complete discussion of the consequences of incorrect group assignment is provided as Supporting Information in the manual.

### 2.7 Transition Dipoles and Infrared Intensities

Infrared intensities are proportional to the squared transition dipole moment:

26 $$I_{0I}\propto \underset{\alpha =x,y,z}{\sum }{|\langle \Psi_{0}|{\mathrm{\mu ̂}}_{\alpha }|\Psi_{I}\rangle |}^{2}$$

The dipole operator is expanded to second order in normal coordinates:

27 $${\mathrm{\mu ̂}}_{\alpha }=\mu_{\alpha }^{\left(0\right)}+\overset{M}{\underset{i=1}{\sum }}\mu_{\alpha }^{\left(i\right)}Q_{i}+\frac{1}{2}\overset{M}{\underset{i=1}{\sum }}\overset{M}{\underset{j=1}{\sum }}\mu_{\alpha }^{\left(ij\right)}Q_{i}Q_{j}$$

where $\mu_{\alpha }^{\left(i\right)}\equiv \partial \mu_{\alpha }/\partial Q_{i}{|}_{0}$ and $\mu_{\alpha }^{\left(ij\right)}\equiv \partial^{2}\mu_{\alpha }/\partial Q_{i}\partial Q_{j}{|}_{0}$ .

#### 2.7.1 Transformation of Dipole Derivatives from ORCA

The ORCA VPT2 output provides the first derivatives in Cartesian coordinates, ∂μ_α_/∂*x*_*k*_. They are transformed to normal coordinates via

28 $$\frac{\partial \mu_{\alpha }}{\partial Q_{i}}=\overset{3N_{\mathrm{at}}}{\underset{k=1}{\sum }}\frac{\partial \mu_{\alpha }}{\partial x_{k}}\frac{L_{ki}}{\sqrt{m_{a}}}$$

where *L*_*ki*_ are the mass-weighted Hessian eigenvectors and *a* = ⌈*k*/3⌉ is the atom index. The result is a (3*N*_at_ × 3) matrix; only the *M* vibrational rows are retained.

The second derivatives are supplied in a mixed representation: ∂^2^μ_α_/∂*Q*_*i*_∂*x*_*k*_, with the first index already in normal coordinates. Only the second Cartesian index requires transformation:

29 $$\frac{\partial^{2}\mu_{\alpha }}{\partial Q_{i}\partial Q_{j}}=\overset{3N_{\mathrm{at}}}{\underset{k=1}{\sum }}\frac{\partial^{2}\mu_{\alpha }}{\partial Q_{i}\partial x_{k}}\frac{L_{kj}}{\sqrt{m_{a}}}$$

The ORCA-provided array has dimensions *M* × 3*N*_at_ × 3. Internally, the resulting *M* × *M* × 3 array is divided by 2 to absorb the factor of 1/2 from the Taylor expansion so that the VCI dipole routine multiplies directly by the stored value.

#### 2.7.2 Dipole Matrix Elements in the VCI Basis

The full dipole matrix element between configurations **m** and **n** consists of three contributions:

**Linear Dipole (One-Body):**

30 $$D_{\alpha }^{\left(1\right)}\left(m,n\right)=\overset{M}{\underset{i=1}{\sum }}\mu_{\alpha }^{\left(i\right)}I_{i}\left(m_{i},n_{i};1\right)S_{\mathrm{all}\backslash i}$$

**Diagonal Second Derivative (One-Body):**

31 $$D_{\alpha }^{\left(2a\right)}\left(m,n\right)=\overset{M}{\underset{i=1}{\sum }}\mu_{\alpha }^{\left(ii\right)}I_{i}\left(m_{i},n_{i};2\right)S_{\mathrm{all}\backslash i}$$

**Off-Diagonal Second Derivative (Two-Body):**

32 $$D_{\alpha }^{\left(2b\right)}\left(m,n\right)=\underset{i<j}{\sum }2\mu_{\alpha }^{\left(ij\right)}I_{i}\left(m_{i},n_{i};1\right)I_{j}\left(m_{j},n_{j};1\right)S_{\mathrm{all}\backslash \{i,j\}}$$

At the HO and VSCF levels, only $D_{\alpha }^{\left(1\right)}$ is used, i.e., the linear dipole surface. Refer the manual provided as Supporting Information for details on how the VSCF intensities are calculated. At the VCI, S-VCI, and SA-VCI levels, all three contributions are evaluated. The quadratic terms $D_{\alpha }^{\left(2a\right)}$ and $D_{\alpha }^{\left(2b\right)}$ are essential for overtone and combination band intensities, which may not be computed in the double-harmonic approximation, and for correctly redistributing oscillator strength among Fermi-resonant states.

The configuration-space dipole matrix is transformed to the eigenstate basis using BLAS: first, **t**_α_ = **μ**_α_ · **c**_0_ (DGEMV), then $\left\langle \Psi_{I}\right|{\mathrm{\mu ̂}}_{\alpha }\left|\Psi_{0}\right\rangle =c_{I}^{T}\cdot t_{\alpha }$ (DDOT). Intensities are globally normalized by default for convenience in spectral visualization and peak assignment. The normalization is performed according to

33 $$I_{0I}^{\mathrm{norm}}=100{Ĩ}_{0I}/\sqrt{\underset{\Delta E\leq E_{\mathrm{cutoff}}}{\sum }{Ĩ}_{0J}^{2}}$$

where *Ĩ*_0*I*_ = ∑_α_ |μ_α,0*I*_|^2^, Δ*E* = *E*_*I*_ – *E*_0_ is the transition energy from the ground state, and *E*_cutoff_ is a user-specified energy threshold with a default value of 4500 cm^–1^, which can be modified. This ensures that the relative intensities are independent of higher-energy states that are often irrelevant for practical comparisons. Transitions with energy above *E*_cutoff_ are not displayed in the spectrum. The normalization is explicitly defined so that the ratios between peaks are invariant with respect to the total number of calculated states.

## 3 Program Architecture and User Utilities

ViBra consists of a Fortran 90/95 computational engine and a Python 3 graphical interface. The computational engine is organized into modular components, with each responsible for a distinct stage of the anharmonic vibrational spectroscopy workflow. The principal modules are summarized in Table 1.

**Table 1:**
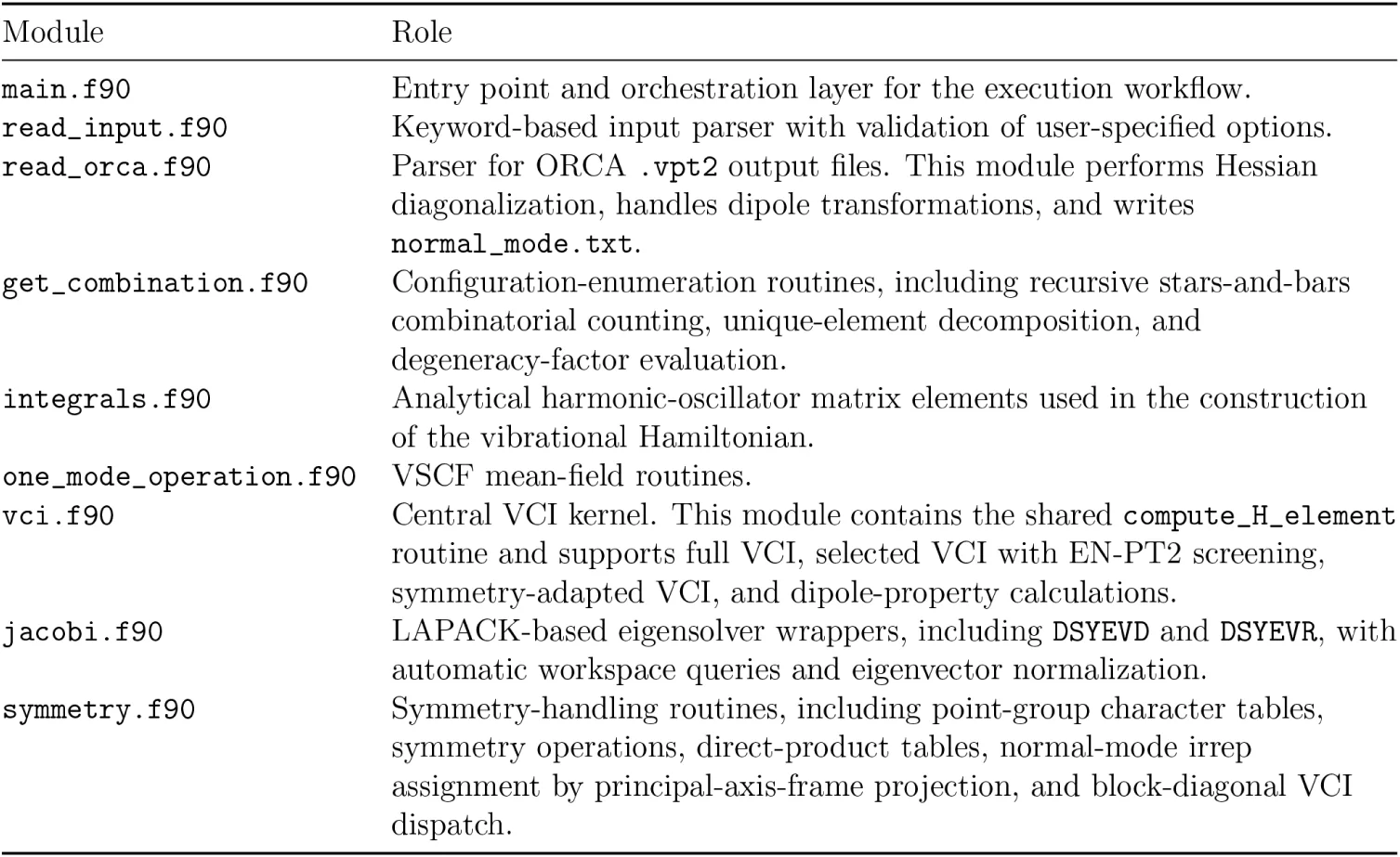
Main Computational Modules in the ViBra Fortran Engine

The engine produces three output files, which can be utilized by the visualization interface:

- vscf.out—complete log: VSCF convergence histories, modal coefficient matrices, VCI configuration list, eigenstate energies, and the three leading CI coefficients with quantum-number assignments;
- intensities.txt—transition frequencies (cm^–1^) and normalized intensities for HO, VSCF, and VCI/SCI methods;
- normal_mode.txt—equilibrium geometry (Å) and Cartesian displacement vectors for all 3*N*_at_ modes.

### 3.1 Graphical User Interface

The CustomTkinter application, Figure 1 (VSCFVCIApp), simplifies job preparation. The user navigates to the ORCA .vpt2 file; the working directory is set automatically. A modal dialog collects all keywords (Table 2), validates their ranges with tooltip guidance, and writes input_vscf.txt. The Fortran executable is launched as a subprocess in a background thread, with real-time output streaming to a scrollable panel. A stop button terminates a running job, and the panel supports saving and clearing operations.

**Figure 1:**
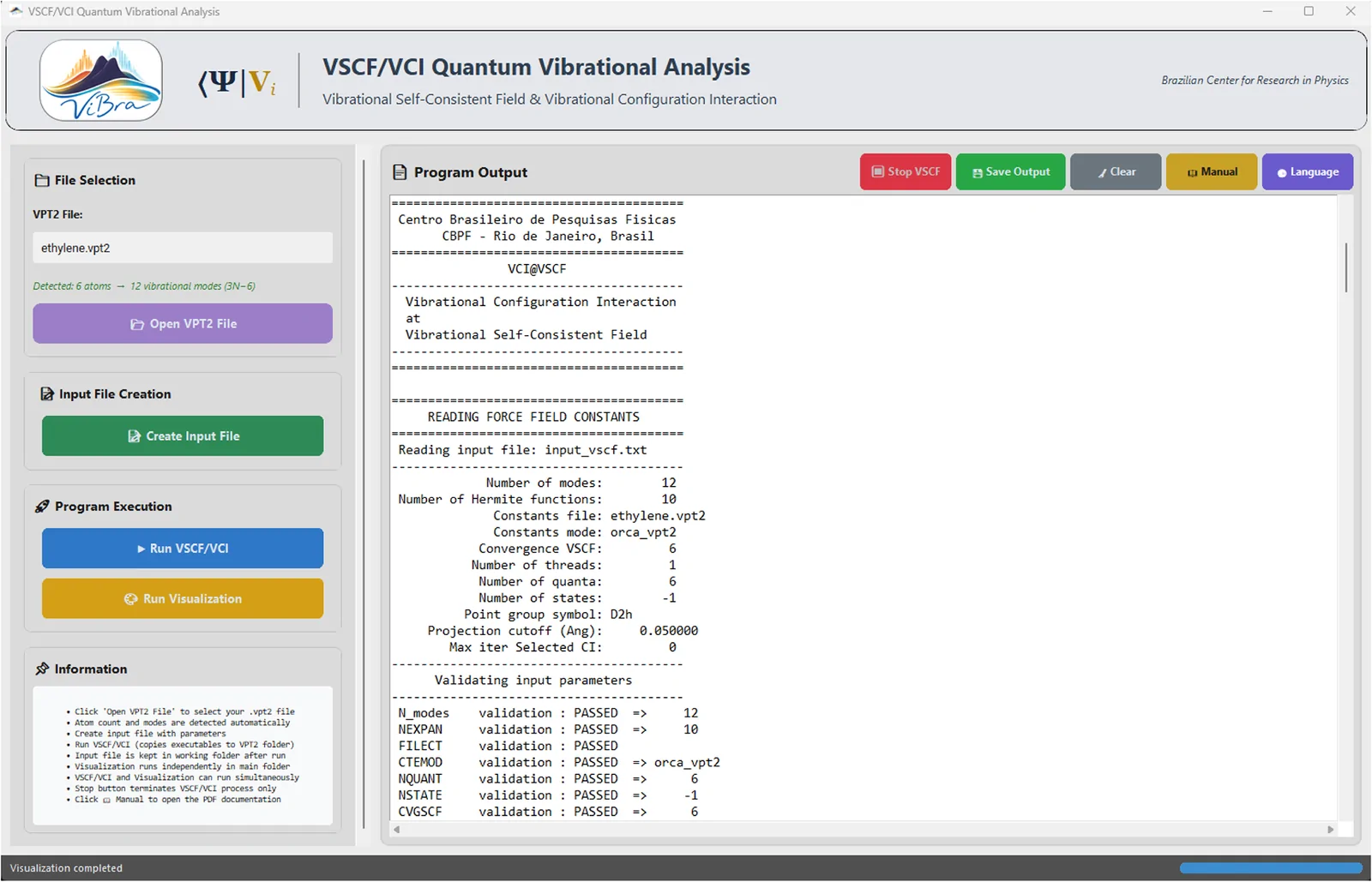
Graphical interface of ViBra, showing the capability to directly load ORCA VPT2 output files, generate input files, and execute the code.

**Table 2:**
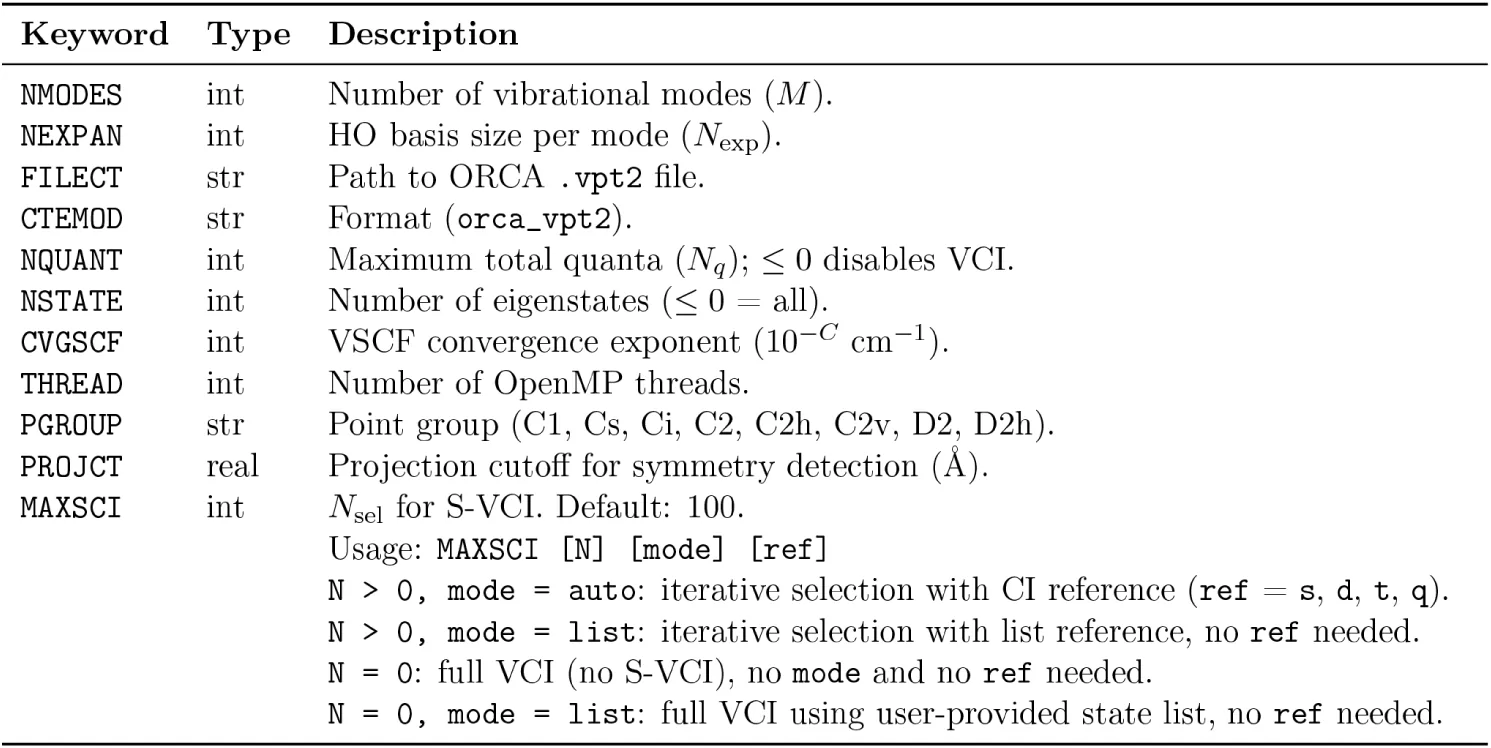
Principal Input Keywords of ViBra

### 3.2 Interactive Spectral Viewer

The viewer, Figure 2 (MainApplication, Python 3, Matplotlib, 3Dmol.js), reads the three output files and provides a rich environment for analysis and comparison:

- **Gaussian broadening:** each transition at frequency ν_*k*_ with intensity *I*_*k*_ is represented as *S*(ν) = ∑_*k*_ *I*_*k*_ exp­[−(ν – ν_*k*_)^2^/(2σ^2^)], with $\sigma =\mathrm{FWHM}/\left(2\sqrt{2\ln2}\right)$ ; fwhm is adjustable via a stepper and direct text entry.
- **Temperature-dependent spectra:** for *T* > 0 K, VCI intensities are Boltzmann-weighted: $I_{k}^{\mathrm{eff}}=I_{k}\left(p_{0}-p_{k}\right)$ , where *p*_*k*_ = exp­(−*hcν*_*k*_/*k*_*B*_ *T*)/*Z* and *hc*/*k*_*B*_ = 1.438777 cm·K. Temperature is adjustable from 0 to 950 K.
- **JCAMP-DX overlay:** experimental spectra in the JCAMP-DX format (.jdx/.dx) can be loaded. Transmittance is automatically converted to absorbance, negative values are clipped, and the result is normalized.
- **ALS baseline correction:** for experimental spectra exhibiting a sloping baseline, an Asymmetric Least Squares (ALS) algorithm^40^ estimates and subtracts a smooth baseline. The cost function *Q*(**z**) = ∑_*i*_ *w*_*i*_(*y*_*i*_ – *z*_*i*_)^2^ + λ∥**Dz**∥^2^ is minimized iteratively, where **D** is the second-difference matrix. The smoothness parameter λ and asymmetry parameter *p* are adjustable via steppers with real-time redisplay.
- **Peak inspection:** clicking a VCI peak label displays the frequency, intensity, and three leading CI coefficients with quantum-number assignments (e.g., “*v*_1_ + 2*v*_3_”). Buttons launch 3D normal-mode animations for any involved mode.
- **3D normal-mode animation:** sinusoidal displacement trajectories (60 frames, adjustable amplitude) are generated from normal_mode.txt, saved as JSON, and rendered via an autogenerated HTML page with an embedded 3Dmol.js viewer supporting play/pause, a frame slider, speed control, and adjustable rendering styles and atomic radii. See Figure 3.
- **Data export:** the displayed spectrum can be saved as a text file and as a 300 dpi PNG image.

**Figure 2:**
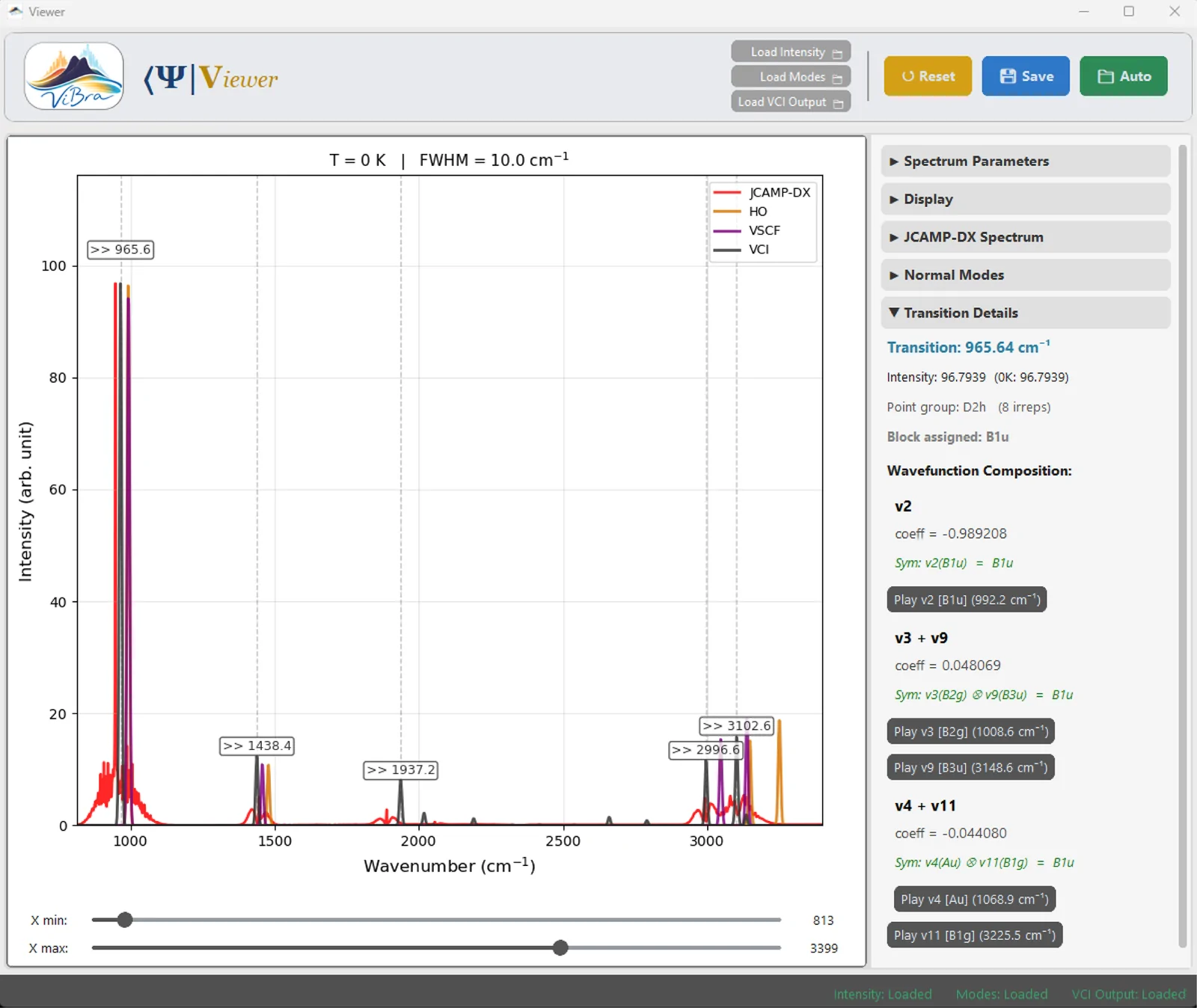
Dedicated ViBra spectrum visualizer, highlighting the capability to directly compare HO, VSCF, and VCI calculations with experimental data, while seamlessly analyzing the peak composition associated with each assigned mode. The displayed results correspond to the ethylene molecule, where the full VCI method was applied, including up to 6 quanta, and 10 HO functions per mode were used in the VSCF calculation. Experimental data were retrieved from the National Institute of Standards and Technology database.^39^

**Figure 3:**
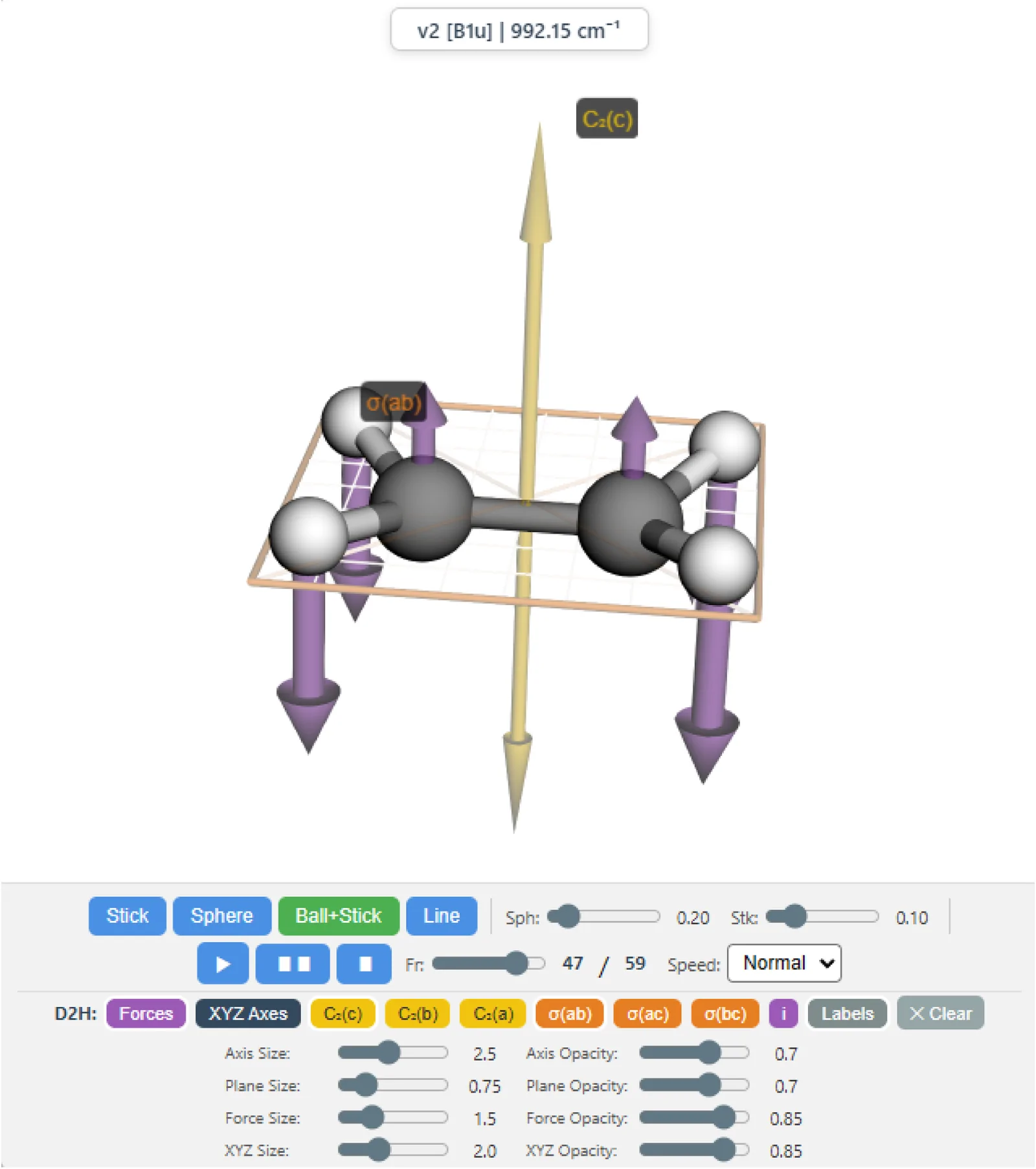
Normal mode animator, featuring the ability to display force vectors and symmetry elements whenever a point group other than C1 is employed.

## 4 Computational Optimization Strategies

ViBra employs several targeted algorithmic optimizations that are essential for making VCI calculations on molecules with *M* ≈ 30–40 and larger tractable. The complete rationale for each strategy is given in the manual, provided as Supporting Information; the most impactful are summarized here.

### 4.1 Overlap Cache in VSCF

At the beginning of each VSCF iteration, all scalar overlaps $\left\langle \varphi_{n_{j}}^{\left(j\right)}\right|Q_{j}^{c}\left|\varphi_{n_{j}}^{\left(j\right)}\right\rangle$ (*c* = 0, ..., 4) are precomputed and cached. The $X_{i}^{\left(p\right)}$ coefficients are then assembled via *O*(1) look-ups rather than repeated $O\left(N_{\exp}^{2}\right)$ double sums.

### 4.2 Vectorized Potentials and Nonzero Flags

Force constants *V*_*ijk*_ and *V*_*ijkl*_ are permutationally symmetric, so a dense *M* ^3^ or *M* ^4^ array wastes memory and access time on redundant duplicates. Instead, each is flattened into a 1D array of length *T*_3_ (respectively, *T*_4_), containing one entry per sorted index combination *i* ≤ *j* ≤ *k* (respectively, *i* ≤ *j* ≤ *k* ≤ *l*), together with precomputed metadata: the distinct mode indices appearing in the term, their multiplicities (e.g., *V*_*iij*_ has mode *i* with multiplicity 2 and mode *j* with multiplicity 1), and a boolean flag marking whether the term exceeds a numerical threshold and is therefore treated as nonzero. This flattening removes redundant storage and lets the Hamiltonian-construction loop iterate directly over the *T*_3_ + *T*_4_ distinct terms rather than the full *M* ^3^ + *M* ^4^ index space, with the nonzero flag skipping negligible terms at no extra cost.

### 4.3 Sparse Pair List

Before building *H*, the code makes a single pass over all configuration pairs and records only those with *d* ≤ 4 differing modes, storing for each such pair the indices of the modes that differ. The Hamiltonian-construction loop then iterates directly over this precomputed list rather than over all |S|(|S|+1)/2 pairs, so pairs that are known in advance to give zero coupling are never visited.

### 4.4 Inverted Potential Index

For a pair differing in *d* modes, a cubic or quartic term contributes only if it involves all *d* differing modes; otherwise, modal orthogonality forces at least one factor to vanish. Scanning the full list of *T*_3_ + *T*_4_ potential terms for every pair would waste most of the work rejecting nonmatching terms. Instead, each mode is preindexed with the list of potential terms it appears in. For a given pair, only the terms attached to the *first* differing mode are examined, and each is quickly checked for the remaining *d* – 1 differing modes. This reduces the average per-pair potential scan from *O*(*T*_3_ + *T*_4_) to $O\left(\frac{T_{3}+T_{4}}{M^{d-1}}\right)$ since a term touches a given mode with probability ∼1/*M*.

### 4.5 Prefix–Suffix Overlap Products

The spectator product *S*_all\*S*_ (eq 20) is needed for every Hamiltonian and dipole contribution. Prefix and suffix arrays of $o_{k}=I_{k}\left(m_{k},n_{k};0\right)$ are computed once per matrix element in *O*(*M*) time, enabling *O*(1) evaluation of any excluded-mode product. For multimode exclusion, the total product is divided by the excluded factors; a fallback explicit product handles the case where any overlap vanishes.

### 4.6 OpenMP Parallelism

The Hamiltonian construction loop over the sparse pair list, the EN-PT2 screening in SCI, and the dipole matrix construction are parallelized with dynamic scheduling and empirically tuned chunk sizes (16-256). Dynamic scheduling is essential because pairs with *n*_diff_ = 0 (diagonal) evaluate all nonzero potential terms, while high-*n*_diff_ pairs evaluate far fewer, creating significant load imbalance. The VSCF stage runs in serial since, with the overlap cache, it completes significantly faster.

### 4.7 LAPACK/BLAS Integration

The VSCF modal matrices are diagonalized with DSYEVD. For VCI/S-VCI, DSYEVD is used when all eigenvalues are requested; DSYEVR computes only the lowest *N*_state_ eigenvalues (if the point group is C1 and no external list is given) for partial spectra, saving both memory and time. Dipole transformations use DGEMV and DDOT.

### 4.8 Memory Considerations

The full VCI Hamiltonian is stored as a dense symmetric matrix, which limits the practical configuration space size to $\left|S\right|∼10^{4}-10^{5}$ depending on available memory (the matrix requires ${|S|}^{2}\times 8$ bytes). The SCI approach directly alleviates this by reducing the matrix dimension. The modal integral table is compact (*M* × (*N*_*q*_ + 1)^2^ × 6 entries). The sparse pair list and inverted index use integer arrays whose size is data-dependent but typically modest compared to the Hamiltonian itself.

## 5 Validating and Using ViBra

### 5.1 Validation of the ViBra Implementation

We carried out a systematic validation of the ViBra code through four complementary benchmarks, progressing from the simplest VSCF level to fully correlated VCI and symmetry-adapted formulations. All geometry files, ORCA inputs and outputs, ViBra inputs and outputs, as well as the Crystal23 output for water, are provided in a public Zenodo data set^41^ for numerical comparison. For the water benchmark (steps 1 and 2), the agreement with reference data is within numerical precision; therefore, we do not report the numerical outputs in the main text. For the validation of the SA-VCI and S-VCI implementations (steps 3 and 4), the complete data set, comprising all vibrational modes, VCI coefficients, and energy contributions for each molecule, is extensive and is provided in its entirety in the Zenodo data set. In the following section (section 5.2), we present the ethylene molecule as a representative case study to illustrate the key findings.

#### 5.1.1 Step 1: Validation of the VSCF Implementation

**Goal:** to verify the correctness of the VSCF module in ViBra. **Action:** we first validated the VCI@VSCF implementation by benchmarking VSCF ground-state energies of a water molecule computed with Crystal23 at the B3LYP/6-31G* level of theory. VSCF calculations were performed using a basis set of 10 harmonic oscillator (HO) functions per vibrational mode. We obtained the reference data (i.e., cubic and quartic force constants and normal-mode frequencies) from the Crystal23 website, where they are provided as part of the anharmonic vibrational tutorial.^15,42^ Therefore, we performed no computations at this level of theory, nor did we make use of Crystal23. **Comparison:** we compared ground-state energies, fundamental transition energies, and optimized modal coefficients. The computed energies show excellent agreement with the reference values, with deviations in the range of 0.01–0.001 cm^–1^. The modal coefficients differ on the order of 10^–3^ or lower, also indicating excellent agreement. **Conclusion:** the VSCF implementation in ViBra is correct and reproduces reference data with numerical accuracy.

#### 5.1.2 Step 2: Validation of the VCI Implementation

**Goal:** to verify the correctness of VCI diagonalization and eigenstate analysis in ViBra. **Action:** we analyzed VCI energies and eigenstate coefficients using the same water molecule benchmark. A total excitation truncation of up to 5 quanta was utilized. **Comparison:** the observed discrepancies between ViBra and Crystal23 are of the same magnitude as those obtained at the VSCF level and are attributed to numerical noise. The most important configurations, together with their numerical contributions, for each VCI state were correctly identified. **Conclusion:** these results demonstrate that the entire workflow, from the initial VSCF step to the final diagonalization of the VCI Hamiltonian matrix, is correctly implemented in ViBra. In particular, the computed eigenvalues (energies) and eigenvectors (VCI coefficients) are in full agreement with the reference results, confirming the correctness of the implementation.

#### 5.1.3 Step 3: Validation of the SA-VCI Implementation

**Goal:** to validate the symmetry-adapted VCI (SA-VCI) formulation against full VCI calculations. **Methodology:** we employed semiquartic force fields, defined here as cubic force constants together with quartic contributions in which at least two vibrational modes are identical. Geometry optimizations, energy minimizations, and force constant computations were carried out using ORCA 6.1 at the wB97X-D4/aug-cc-pVTZ level of theory. The test set comprised molecules spanning several point-group symmetries: HFCO (*C*_*s*_), N_2_H_4_ (*C*_2_), H_2_O (*C*_2*v*_), *trans*-N_2_H_2_ (*C*_2*h*_), and C_2_H_4_ (*D*_2*h*_). **Comparison:** for all molecules examined, the low lying state energies obtained from full VCI and SA-VCI agree to within numerical noise, on the order of 10^–2^ cm^–1^ or better. **Conclusion:** the SA-VCI formulation is correctly implemented and reproduces full VCI results to numerical precision. **Computational details:** our choice of functional and basis set was driven not by the pursuit of highly accurate vibrational frequencies, but by considerations of balanced computational cost and methodological robustness. The range-separated hybrid functional wB97X-D4 combined with the aug-cc-pVTZ basis set is widely used in benchmark vibrational studies, including several entries at the CCCBDB/NIST frequency database.^43^ In the VSCF step, we adopted a basis of 10 harmonic-oscillator functions per vibrational mode, a choice motivated by the rapid convergence of VSCF energies with respect to the number of HO functions and by the fact that this basis provides a balanced description of vibrational states with few quanta of excitation per mode.^15^ For both the full VCI and SA-VCI expansions, we included configurations with up to 4 quanta. **Important note on symmetry handling:** despite the tight convergence criteria for minimizing the geometries, no symmetry constraints were imposed during the ORCA computations; geometries, Hessians, and force constants were evaluated entirely without point-group restrictions. This choice was deliberate for the present validation study: our objective is also to probe the robustness of the SA-VCI implementation against minute numerical asymmetries that may arise in the quartic force field; we caution that for production-level applications, users are strongly advised to enforce appropriate symmetry constraints during the electronic structure calculations.^18^ Even tiny deviations introduced by a non-symmetrized quartic force field can accumulate, leading to larger errors, specially for higher energy states.

#### 5.1.4 Step 4: Three-Tier Benchmark of Intensities and Variational Selection

**Goal:** to validate the intensity calculations and the selected-state VCI (S-VCI) procedure. **Action:** we carried out a three tier benchmark that begins from the standard double harmonic approximation and progressively includes higher-order effects. The electronic structure calculations were the same as those described in Step 3, and all molecules in the test set were benchmarked. **Tier 1 (DHA validation):** we benchmarked intensities obtained with ORCA 6.1 and ViBra within the harmonic approximation using linear dipole derivatives, finding identical results for all molecules, noting that ViBra intensities are internally normalized. This step corresponds to the full double harmonic approximation (harmonic frequencies + linear dipole derivatives). **Tier 2 (Electrical anharmonicity):** we analyzed ViBra energies and intensities including also second-order dipole derivatives, both within the full VCI framework and within the symmetry-adapted VCI (SA-VCI) formulation. For all molecules, the resulting energies agree within numerical noise, on the order of 10^–2^ cm^–1^ or lower. The intensities obtained from SA-VCI and full VCI are identical within numerical precision. **Tier 3 (S-VCI variational validation):** to validate the S-VCI (selected states via EN-PT2), we analyzed the variational behavior of the method by systematically increasing the number of included states. As expected for a variational approach, all state energies decreased monotonically upon enlarging the configuration space by increasing MAXSCI. **Conclusion:** the intensity implementations (both DHA and with second-order derivatives) are correct, and the S-VCI selection procedure behaves variationally as expected.

### 5.2 Using ViBra

To demonstrate the efficiency of the software, particularly for VCI and its variants, we consider the ethylene (C_2_H_4_) molecule. Its *D*_2*h*_ symmetry provides a clear example of the computational advantages gained from block diagonalization. Since VSCF calculations are computationally inexpensive, we do not provide a detailed performance analysis for this step. We employ a fixed basis of 10 harmonic oscillator functions per vibrational mode and an energy convergence threshold of 10^–6^ cm^–1^ in the VSCF procedure preceding all VCI and VCI-variant calculations. It is important to note that all frequency calculations performed with ViBra were carried out on an 11th Gen Intel Core i9-11900K processor, with the CPU frequency capped at 3.5 GHz. The machine was equipped with 2 × 32 GB of RAM running at 3200 MHz, and cache sizes of 640 KB (L1), 4.0 MB (L2), and 16.0 MB (L3). The code is compiled with ifx, Intel’s next-generation LLVM-based Fortran compiler, using -O3 for aggressive optimization including loop transformations and vectorization. Parallelism is enabled at multiple levels: /Qopenmp activates explicit OpenMP directives, /threads enables compiler autoparallelization of loops, and /Qmkl:parallel links the threaded Intel Math Kernel Library for parallel BLAS/LAPACK routines. The /Qm64 flag targets the 64-bit instruction set, while /heap-arrays allocates temporary arrays on the heap to prevent stack overflow from large automatic arrays. Finally, /fpscomp:logicals enforces compatibility with older Intel Fortran logical behavior. A precompiled executable is provided alongside the source code in the repository.

Using this computational setup, we examine the dependence of basis size, energy convergence, and diagonalization cost on the maximum number of quanta included in the VCI expansion, as summarized in Table 3. Note that a single core was used for this benchmark. The Hamiltonian dimension increases rapidly as the maximum excitation level increases. Increasing the maximum number of allowed quanta from 4 to 5, and subsequently from 5 to 6, approximately triples the number of vibrational configurations at each stage, resulting in an approximately ninefold increase in the number of Hamiltonian matrix elements (and hence memory footprint) for every additional maximum quanta.

**Table 3:** Convergence of VCI Energies and Computational Cost with Increasing Excitation Quanta for Ethylene^a^

# Quanta	# States	*t*_diag_	*t*_tot_	ZPE	*E*_1_	Δ*E*
2	91	0	0	11191.4056	12036.0327	844.6271
3	455	0	0	11129.7226	12034.6711	904.9484
4	1820	1	2	11127.0028	11959.2915	832.2886
5	6188	38	44	11126.7737	11953.6805	826.9069
6	18564	1400	1438	11125.5159	11952.9510	827.4351
6(20)	18564	632	670	11125.5159	11952.9510	827.4351

a The final row corresponds to partial diagonalization of the 6-quanta Hamiltonian, requesting only the lowest 20 eigenvalues. All energies are reported in cm^–1^ and timings in seconds.

The zero point energy (ZPE) and the first excited state energy (*E*_1_) both decrease monotonically as the maximum excitation level is increased, as expected from the systematic enlargement of the VCI configuration space. However, their convergence rates differ significantly. The ZPE is already effectively converged when a maximum of 3 quanta is included, whereas *E*_1_ requires at least 4 quanta to reach a practically stable value. This behavior is consistent with the semi-quartic character of the force field. Because the reductions in the ZPE and *E*_1_ are not proportional, the transition energy, Δ*E* = *E*_1_ – ZPE, does not exhibit monotonic convergence. Instead, it increases by approximately 60 cm^–1^ when the maximum excitation level is increased from 2 to 3 quanta before decreasing to a value below that obtained in the calculation with 2 quanta.

The apparent stabilization at 4 quanta should not be interpreted as strict numerical convergence. Rather, it indicates that the remaining changes are sufficiently small for the practical applications considered here and do not justify the substantial additional computational expense associated with the inclusion of higher excitation levels. Therefore, a maximum of 4 quanta provides an appropriate compromise between accuracy and computational efficiency for routine calculations. When higher numerical accuracy is required, however, calculations including 5 or 6 quanta may still be necessary.

This need for increased accuracy must be balanced against the rapidly increasing computational costs. Both the memory requirements and the CPU time increase dramatically as the maximum number of allowed quanta is increased from 4 to 6, with matrix diagonalization becoming the dominant computational bottleneck. For the largest calculation, corresponding to a maximum of 6 quanta and a Hamiltonian containing 18564 vibrational configurations, diagonalization with DSYEVD required 1400 s (23.3 min) out of a total runtime of 1438 s.

To reduce this computational expense, a partial diagonalization was also performed using DSYEVR, requesting only the lowest 20 eigenvalues. This approach avoids constructing and storing the complete set of eigenvectors, reducing the diagonalization time to 632 s (10.5 min) and the total runtime to 670 s, as reported in the final line of Table 3. Although the eigenvectors are no longer available for subsequent intensity calculations, the reduction in computational cost is substantial and becomes increasingly advantageous when only a limited number of state energies are required. Consequently, for applications that require only the ZPE, such as the calculation of zero-point energy corrections to electronic energies in chemical reaction transition barrier calculations, partial diagonalization provides a particularly efficient strategy.

The full diagonalization of the 6-quanta Hamiltonian, although manageable for ethylene, remains computationally demanding. This motivates the use of selected VCI (S-VCI) and symmetry-adapted VCI (SA-VCI) to further reduce the cost while preserving accuracy. Table 4 compares the performance of both approaches using 8 OpenMP threads; the reported real time refers to the total wall-clock time encompassing state selection (S-VCI) or symmetrization (SA-VCI), Hamiltonian construction, diagonalization, and intensity evaluation. In the S-VCI columns, the number following the slash denotes the value of the MAXSCI keyword, which controls how many states are retained by the perturbative selection criterion. Increasing MAXSCI systematically enlarges the selected space and drives the energies toward the full-VCI limit. Already at MAXSCI = 50, the ZPE and *E*_1_ lie within 1.6 and 2.3 cm^–1^ of the full-VCI result, respectively, and further increasing the keyword from 100 to 300 yields only marginal improvement. This indicates that low MAXSCI values may filter too aggressively, discarding states that contribute significantly to the correlation energy (see Section 6.1); a moderate threshold (here 50) already captures the essential physics. The optimal value is molecule-dependent and should be calibrated, when possible, by comparing a small-basis S-VCI calculation against full VCI, as done here. Notably, within the S-VCI kernel, we used the default VCISD reference, with external states generated up to NQUANT = 6.

**Table 4:** Performance Comparison of Selected VCI (S-VCI) and Symmetry-Adapted VCI (SA-VCI) against Full VCI for Ethylene at the 6-Quanta Level^a^

Method	# States	CPU time (s)	Real time (s)	ZPE (cm^–1^)	*E*_1_ (cm^–1^)
S-VCI/50	3193	31	4	11127.0820	11955.2475
S-VCI/100	5921	138	18	11126.7083	11953.8296
S-VCI/150	7895	208	28	11126.5374	11953.6018
S-VCI/200	9241	303	40	11126.5056	11953.4682
S-VCI/250	10339	414	55	11126.4265	11953.4365
S-VCI/300	11140	494	66	11126.3749	11953.4153
Full VCI	18564	2005	265	11125.5159	11952.9510
SA-VCI (*D*_2*h*_)	18564	89	13	11125.5202	11952.9591
SA-VCI (*C*_*i*_)	18564	601	81	11125.5177	11952.9550

a S-VCI rows are labeled by the MAXSCI keyword value. All calculations used 8 OpenMP threads.

Turning to symmetry-adapted VCI, the *D*_2*h*_ point group of ethylene partitions the Hamiltonian into eight irreducible representation blocks. In the ideal scenario where all blocks are of equal size, each block has dimension *N*/8, and the cubic scaling of diagonalization (e.g., using the DSYEVD subroutine) reduces the total cost by a factor of 8 × (1/8)^3^ = 1/64, yielding a theoretical maximum speedup of 64×. In our implementation, the symmetry blocks are processed sequentially, but each block diagonalization is executed using all available OpenMP threads, rather than distributing blocks across threads and subdividing the thread team. This design ensures that the full multithreaded capability of the diagonalizer is applied to every block, avoiding load imbalance when the number of symmetry operations does not evenly divide the thread count.

In practice, the observed reduction in real time is approximately 20× (from 265 s for full VCI to 13 s for SA-VCI/*D*_2*h*_). The primary reason for the 20× speedup is simple: not all operations scale cubically, so a 64× speedup is never expected; it will always be less. Moreover, it should be noted that SA-VCI and full VCI employ different computational kernels, and the timings reported in Table 4 reflect the total execution time from initialization to completion, not solely the diagonalization step. Furthermore, the irreducible blocks are not perfectly equal in size, reducing the theoretical gain. Also, the nondiagonalization overhead (symmetrization, integral transformation, etc.) accounts for a larger fraction of the total time in the SA-VCI run, further diluting the speedup. Finally, the per-block matrices (around 2300 × 2300) may be too small to keep all threads fully utilized, leading to higher parallel overhead than in the full VCI diagonalization of the much larger unblocked matrix. These effects are partially offset by improved cache efficiency when blocks or specific operations require arrays that fit into lower cache levels, but the net result is a reduction from the ideal 64× to ∼20×.

Notably, the gap between ideal and observed speedup narrows as the number of symmetry blocks decreases. Using a smaller symmetry group, such as *C*_*i*_, yields only two blocks, a theoretical speedup of 4×, and an observed reduction to 81 s—an efficiency of 82% relative to the ideal, compared to 31% for the *D*_2*h*_ case. The larger block size in *C*_*i*_ restores diagonalization as the dominant cost, and the ∼2300 × 2300 blocks are replaced by matrices roughly 16 times larger, ∼9200 × 9200, which make better use of the available threads. This trend indicates that our sequential-block parallelization strategy performs best when blocks are large enough to saturate the multithreaded overhead.

Importantly, the energies obtained with SA-VCI/*D*_2*h*_, SA-VCI/*C*_*i*_, and full VCI are numerically equivalent to within a few hundredths of a cm^–1^, confirming that symmetry adaptation introduces no approximation beyond block diagonalization. A word of caution is warranted: assigning normal modes to irreps requires the correct point group. Using a lower symmetry than the true molecular symmetry (e.g., *D*_2*h*_ for benzene instead of *D*_6*h*_) can lead to incorrect irrep assignments, particularly for degenerate modes. It is therefore advisable to run a low-quanta SA-VCI calculation and verify that the resulting energies match the corresponding full-VCI values before proceeding to larger bases.

## 6 Selected VCI as a Bridge to Quantum Simulation Algorithms

The selected VCI machinery in ViBra can also be used in a quantum-centric workflow for vibrational problems. In a conventional ViBra calculation, the initial and selected spaces are generated classically using the procedures described in Section 2.5. In the quantum-centric setting considered here, a quantum-sampling routine instead provides an externally generated seed set of vibrational configurations. ViBra then acts as the classical selected-VCI engine: it can construct and diagonalize the projected anharmonic Hamiltonian directly in that seed or optionally enlarge it through EN-PT2-guided configuration selection before evaluating energies, eigenvectors, transition moments, and spectra.

This division of labor is closely related to recent sample-based quantum diagonalization strategies in electronic-structure theory. In sample-based quantum diagonalization (SQD) and sample-based Krylov quantum diagonalization (SKQD), a quantum processor is used to generate basis-state samples, while the final Hamiltonian projection, diagonalization, and observable evaluation are performed classically.^30,31,44^ The same principle can be applied to vibrational structure. Instead of electronic occupation strings, the samples correspond to vibrational configurations

$$n=\left(n_{1},...,n_{M}\right)$$

where *n*_*i*_ labels the occupied modal or harmonic-oscillator level of mode *i*. The sampled configurations define a compact subspace $S_{Q}$ , and ViBra evaluates

34 $$H_{\alpha \beta }^{\left(S_{Q}\right)}=\left\langle \Phi_{\alpha }\right|{Ĥ}_{\mathrm{vib}}\left|\Phi_{\beta }\right\rangle ,,\Phi_{\alpha },\Phi_{\beta }\in S_{Q}$$

using the same modal-integral and Hamiltonian-construction kernels employed in the classical VCI and S-VCI calculations. This distinction is central: ViBra does not replace the quantum sampling step. The quantum-facing routine supplies the initial seed configurations, while ViBra converts that seed into a projected VCI calculation and, when requested, systematically enriches it through classical EN-PT2-guided selection.

Figure 4 summarizes the proposed workflow. The starting point is the same as in a conventional anharmonic vibrational calculation: electronic-structure calculations provide normal-mode frequencies, cubic and quartic force constants, and dipole derivatives. From these data, ViBra constructs the VSCF modal basis. Before quantum sampling, the vibrational Hamiltonian is represented in the complete total-quanta-truncated product basis of ground-state VSCF modals,

35 $$B\left(N_{q}\right)=\left\{\left|\Phi_{n}\right\rangle :\overset{M}{\underset{i=1}{\sum }}n_{i}\leq N_{q}\right\},,D\left(M,N_{q}\right)=|B\left(N_{q}\right)|=\left(\begin{array}{l} N_{q}+M\\ M \end{array}\right)$$

**Figure 4:**
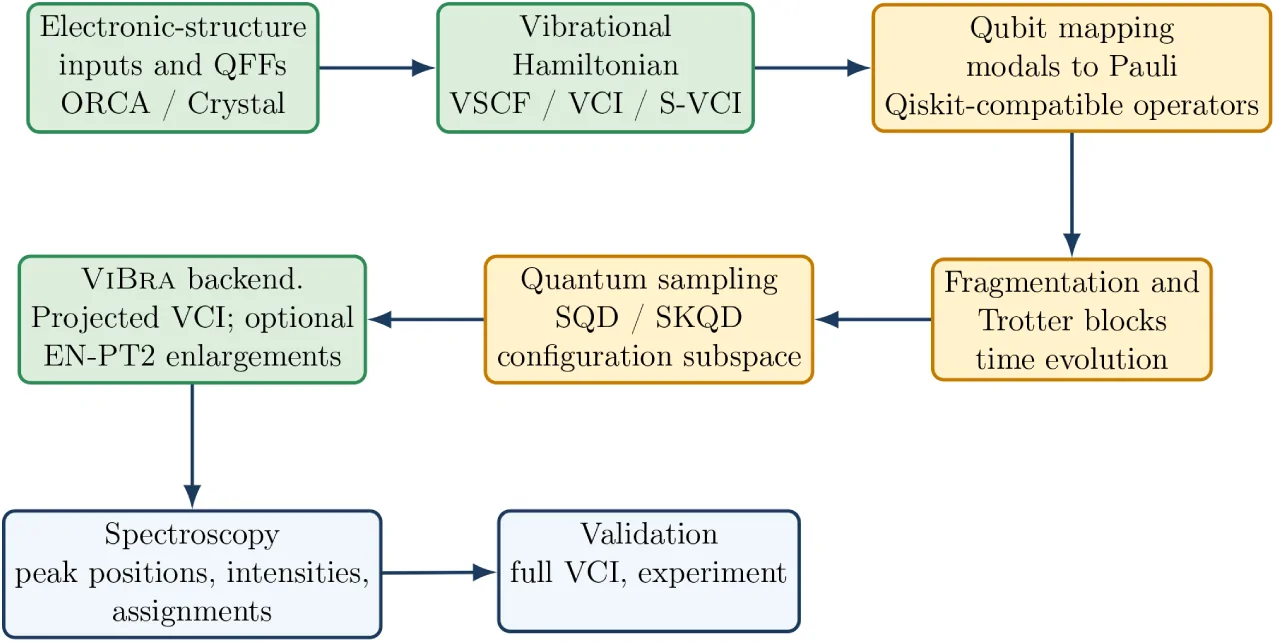
Quantum–classical workflow connecting ViBra (green) to quantum vibrational spectroscopy algorithms. The first green Hamiltonian block denotes construction of the Hamiltonian in the complete *N_q_*-truncated configuration basis; it does not denote a full-space classical diagonalization. The quantum-facing part of the workflow (yellow) maps this Hamiltonian to qubit operators and uses fragmentation and Trotterized time evolution to generate samples. The sampled configuration space is then passed back to ViBra, which constructs and diagonalizes the projected VCI Hamiltonian, optionally after EN-PT2-guided enlargement, and evaluates spectroscopic observables (blue). These results can then be validated against full VCI or experimental data when available.

At this stage, the quantum-sampled space $S_{Q}$ is not yet known. Consequently, the present implementation retains the same *D*(*M*, *N*_*q*_) × *D*(*M*, *N*_*q*_) Hamiltonian-construction and storage requirements as the corresponding stage of a full VCI­(*N*_*q*_) calculation. No full-space eigenvalue problem is solved to generate the quantum samples; full VCI diagonalization is performed separately only to provide the validation reference. The subsequent Pauli mapping and classical circuit simulation introduce separate costs. Thus, this proof of concept does not remove the full-space Hamiltonian-construction bottleneck and is not presented as evidence of an end-to-end quantum advantage.

For the quantum-facing part of the workflow, this Hamiltonian is mapped to qubit operators using a boson-to-qubit or modal-to-qubit representation. In the compact configuration-index encoding employed here, the *D*(*M*, *N*_*q*_) configurations are mapped bijectively to computational-basis states of an *n*_qb_ = ⌈log_2_ *D*(*M*, *N*_*q*_)⌉-qubit register, with any remaining basis states treated as unused padding. After mapping, the Hamiltonian takes the Pauli form

36 $${Ĥ}_{\mathrm{vib}}\to {Ĥ}_{q}=\underset{k}{\sum }c_{k}P_{k}$$

where *P*_*k*_ are tensor products of single-qubit Pauli operators. The mapped Hamiltonian can then be partitioned into mutually commuting blocks of Pauli operators,

37 $${Ĥ}_{q}=\underset{\nu }{\sum }{Ĥ}_{\nu }$$

so that a real-time propagator can be approximated using a product formula, such as the first-order Suzuki-Trotter decomposition

38 $${Û}_{\mathrm{Trot}}\left(t\right)={[\prod_{\nu }\exp\left(-i{Ĥ}_{\nu }t/r\right)]}^{r}+O\left(t^{2}/r\right)$$

with *r* Trotter steps. For the initial state of the evolution, we have used a normalized superposition of the vacuum and all first single-mode excitations. The resulting circuits can be used either to generate samples from states relevant to the low-energy vibrational spectrum or to estimate a time-domain correlation function directly.

At each sampled evolution time, the simulated qubit register is sampled in the computational basis. Each simulator shot produces an *n*_qb_-bit outcome $b=\left(b_{1}...,b_{n_{\mathrm{qb}}}\right)$ , hereafter referred to as a sampled computational-basis bit string. No quantum hardware measurements were performed. Under the compact configuration-index encoding employed here, each outcome assigned to one of the *D*(*M*, *N*_*q*_) physical configurations is decoded through the inverse index map as n=D(b) . The remaining $2^{n_{\mathrm{qb}}}-D\left(M,N_{q}\right)$ outcomes correspond to unused padding states and are discarded. The quantum-sampled subspace is then

39 $$S_{Q}=\left\{n|n=D\left(b\right),b\in \mathrm{samples},c\left(b\right)\geq c_{\min}\right\}$$

where *c*(**b**) is the observed count of bit string **b** and *c*_min_ is an optional count threshold. In practice, the sampled space can be formed as the union of configurations obtained from multiple state preparations, time steps, or Krylov-like states. Once $S_{Q}$ is defined, no additional quantum measurements are required to evaluate the projected Hamiltonian matrix elements: they are computed classically by ViBra from the force field and modal integrals.

The quantum-facing components used in this proof-of-concept are implemented outside the main ViBra codebase. In particular, the routines for modal-to-qubit mapping, Pauli-operator construction, and Trotterized time evolution follow the implementation provided in refs 45 and 46. These external routines are used only to generate the qubit Hamiltonian, fragmented evolution operators, quantum circuits, and simulated samples. The subsequent construction of the projected vibrational Hamiltonian, diagonalization of the sampled configuration space, and evaluation of transition intensities were performed by ViBra.

### 6.1 Workflow Validation for H_2_O

As a proof-of-concept demonstration, we applied this workflow to the water molecule. Water is a convenient initial test system: its three vibrational modes keep the full, truncated VCI problem exactly tractable while still incorporating nontrivial anharmonic couplings and infrared-active fundamentals. We performed the calculation for several values of truncation of the configuration space *N*_q_, varying it from 2 to 12, with the full VCI(12) calculation serving as our reference. All ViBra calculations in this section were performed using NEXPAN = 15, CVGSCF = 6. We used the same semiquartic force field data (wB97X-D4/aug-cc-pVTZ) as in the validation section (steps 3 and 4).

All quantum-facing calculations in this study were performed using a simulator; no quantum hardware was used. For each value of *N*_*q*_, we prepared a different quantum circuit, and we evolved them with 4 Trotter steps separated by a dimensionless time δ = 0.1, or a physical time step of 0.531 ps, and each circuit was sampled 1000 times. The number of qubits, as well as other relevant information on the circuits, is provided in Table 5.

**Table 5:** Gate Counts for the Bosonic SKQD Circuits

*N*_*q*_	Qubits	1 qubit gates	2 qubit gates
2	4	1421	494
3	5	5887	2142
4	6	23902	8894
5	6	23895	8894
6	7	96274	36222
7	7	96283	36222
8	8	386446	146174
9	8	386414	146174
10	9	1548396	587262
11	9	1548438	587262
12	9	1548446	587262

The sampled bit strings were decoded into vibrational configurations, and the union of the configurations over all time steps defines the quantum-generated seed space $S_{q}\left(N_{q}\right)$ . The results reported below correspond to \plot_water_qpu_bench\bosonic_selected_states_water_ *N*_q_ _quanta in the database.^41^

The sampled configurations were supplied to ViBra in list-reference mode. With a MAXSCI 0 list, ViBra constructs and diagonalizes the Hamiltonian directly in $S_{Q}$ . With a MAXSCI *m* list, where *m* = 1, 2, or 3, the sampled list is instead used as the reference for an optional classical enlargement. External configurations are ranked for each targeted root using the Epstein–Nesbet second-order perturbative importance measure described in Section 2.5. The highest-ranked configurations are added to the quantum seed, duplicates are removed, and the Hamiltonian is variationally rediagonalized in the resulting space,

$$S^{\left(m\right)}=S_{Q}\cup S_{\mathrm{EN}}^{\left(m\right)}$$

Table 6 reports the total number of states obtained in each case.

**Table 6:** Dimensions of the Quantum-Sampled Seed Spaces and of the Spaces Obtained after Optional EN-PT2-Guided Enlargement by ViBra^a^

*N*_*q*_	5	6	7	8	9	10	11	12
VCI	56	84	120	165	220	286	364	455
Quantum seed, MAXSCI = 0	51	65	82	74	74	61	62	53
ViBra-enlarged, MAXSCI = 1	84	98	132	112	116	98	97	88
ViBra-enlarged, MAXSCI = 2	104	119	155	131	143	121	118	113
ViBra-enlarged, MAXSCI = 3	117	131	172	147	160	132	134	123

a The row with MAXSCI = 0 corresponds to the direct use of the externally sampled seed. The remaining rows are quantum-seeded spaces that have been enlarged by ViBra and variationally rediagonalized. The VCI row gives the maximum number of states for a given *N_q_*, which is $\left(\begin{array}{l} N_{q}+3\\ 3 \end{array}\right)$ , where 3 is the number of modes for water. Note that the enlarged spaces exceed their respective counts because the enlargement was performed, for all *N_q_* (in the qubit mapping), using NQUANT = 12 in ViBra.

Thus, the quantum-facing routine generates the initial seed configurations, while ViBra can either analyze that seed directly or enlarge it through a classical EN-PT2-guided selection. The EN-PT2 quantities are used only to rank additional configurations; they are not added as perturbative corrections to the reported energies, which are eigenvalues obtained by variational rediagonalization of the final projected Hamiltonian.

For *N*_*q*_ = 2–4, the sampling procedure retained every configuration in the corresponding VCI space. Those calculations, therefore, do not test configuration filtering and are omitted from the following discussion. At *N*_*q*_ = 5, 51 of the 56 available configurations were retained, and we consequently discuss the results from *N*_*q*_ = 5 onward.

Table 6 shows that the absolute size of the sampled seed is nonmonotonic with *N*_q_: the directly sampled lists contain between 51 and 82 configurations. Relative to the available space, however, the retained fraction decreases from 51/56 (91.1%) at *N*_q_ = 5 to 53/455 (11.6%) at *N*_q_ = 12. Increasing the sampling cutoff, therefore, does not, under the present sampling protocol, guarantee a larger seed or a more complete representation of a target eigenstate. The identity and connectivity of the sampled configurations are at least as important as their numbers.

To assess the physical quality of the resulting active spaces, we considered the ground state together with the nine states whose dominant reference configurations span the fundamentals, overtones, and combination bands through two quanta. Figure 5 reports the signed deviation in total energy for each of these states as a function of active-space size. For a given reference configuration, we identify the corresponding root as the eigenvector to which that configuration contributes the largest wave function coefficient.

**Figure 5:**
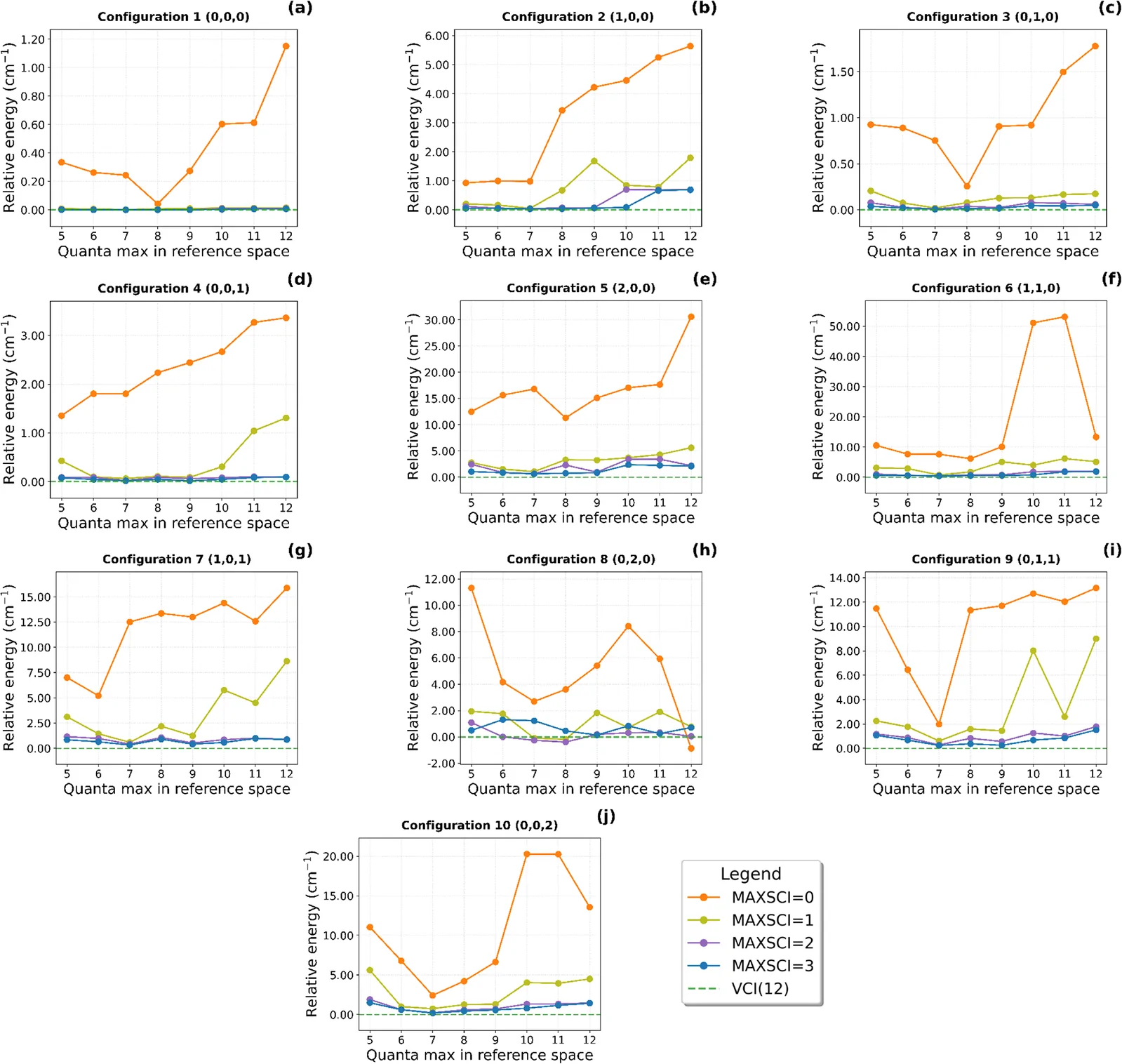
Signed energy deviation, for the water molecule as a function of the maximum total quanta *N_q_* = 5–12 used to generate the quantum-sampled seed. Panel (a) shows the ground state; panels (b)–(j) show the nine states dominated by the indicated configurations. MAXSCI = 0 denotes direct variational diagonalization of the sampled seed, while MAXSCI = 1–3 denotes EN-PT2-guided enlargement of that seed by ViBra followed by variational rediagonalization. EN-PT2 is used only for configuration ranking; no perturbative correction is added to the reported energies.

Direct diagonalization of the quantum seed reproduces the zero-point energy to approximately 1 cm^–1^ across the cutoff range and generally performs well for the fundamentals, with deviations of about 5 cm^–1^. Larger and more variable deviations occur for the two-quantum manifold (combinations and overtones). In particular, the direct-space diagonalization deviations (i.e., MAXSCI = 0) reach approximately 50–60 cm^–1^ for the configurations shown in Figure 5f.

The agreement with the VCI benchmarks improves remarkably after EN-PT2-guided configuration-space enlargement by ViBra, at the cost of larger projected Hamiltonians and correspondingly longer calculations. Nevertheless, here, even in the worst case, the MAXSCI = 3 list (172 of 455 states) comprises only 38% of the full VCI(12) space, a substantial reduction. Already at MAXSCI = 1, errors that previously exceeded 50 cm^–1^ for combinations and overtones drop below 5–7 cm^–1^ for all ten states considered. Moving from MAXSCI = 2 to 3 tightens this further, to roughly 1 cm^–1^, showing an excellent agreement with the VCI(12) reference.

However, two caveats apply to the assignment scheme. First, although the largest coefficient typically exceeds approximately 0.95, the corresponding eigenvector generally retains non-negligible contributions from other configurations; therefore, the assignment reflects the dominant zeroth-order character rather than a pure state. Second, and more importantly, tracking a state by its dominant coefficient does not guarantee that its energy will decrease monotonically as the active space is enlarged. Because new configurations entering the expanded space can generate additional roots lower in energy than the state of interest, the ordinal positions of every higher root shift accordingly. As a result, comparing the energy assigned to a given configuration label across two calculations is not equivalent to comparing the same eigenvalue of the CI matrix; the underlying variational bound applies to the ordinal spectrum of eigenvalues, not to states tracked by the configuration label. Some of the deviations reported in Figure 5 therefore reflect this behavior.

Despite these caveats, we adopt this configuration-based tracking scheme because it offers a practically accessible way to monitor convergence for states with a clear spectroscopic character. An alternative based on wave function overlap between successive active spaces would avoid the reordering artifact in principle, but it requires storing and comparing full coefficient vectors across calculations of different dimensions, which is considerably more costly and less transparent to report than simply following the dominant configuration. For most states considered here, this simpler approach works well: the dominant coefficient stays high and stable across all active-space sizes tested, and the corresponding energy decreases monotonically toward its converged value as the space grows, even when the ordinal position of the root itself shifts.

Figure 5 illustrates both caveats directly. We focus on this configuration because it shows the clearest case of nonmonotonic energy convergence among those we tracked and because the full calculation allows us to confirm its physical origin directly. The data discussed below correspond to *N*_q_ = 12.

At this reference point, the energy assigned to (0,2,0) is 12086.4 cm^–1^ at MAXSCI = 0 (53 reference configurations), rises to 12087.9 cm^–1^ at MAXSCI = 3 (123 configurations, after EN-PT2 selection), and settles at 12087.2 cm^–1^ in the full VCI(12) calculation (455 configurations). The energy, therefore, does not decrease monotonically toward its converged value: it first drops below the converged VCI(12) value at MAXSCI = 0, then overshoots by roughly 1.5 cm^–1^ at MAXSCI = 3, which sits at 0.7 cm^–1^ above the VCI(12) result.

The origin of this behavior becomes clear upon inspecting the (0,2,0) root’s mixing partners at each stage, and Figure 6 supports the discussion below. Across all active-space sizes considered (MAXSCI = 0 to 3 and the full VCI(12)), the dominant secondary contribution is consistently the (0,1,2) configuration, with a coefficient in the range 0.140–0.150. The next-largest contribution, however, changes character with active-space size: at MAXSCI = 0 it comes from (0,0,2) (c = 0.053); at MAXSCI = 3 it is replaced by (5,0,0) (c = 0.122); and in the converged VCI(12) calculation it is replaced again, by (2,1,0) (c = 0.099). Notably, (2,1,0) is absent from the MAXSCI = 0 reference list and only enters the active space once MAXSCI ≥ 1.

**Figure 6:**
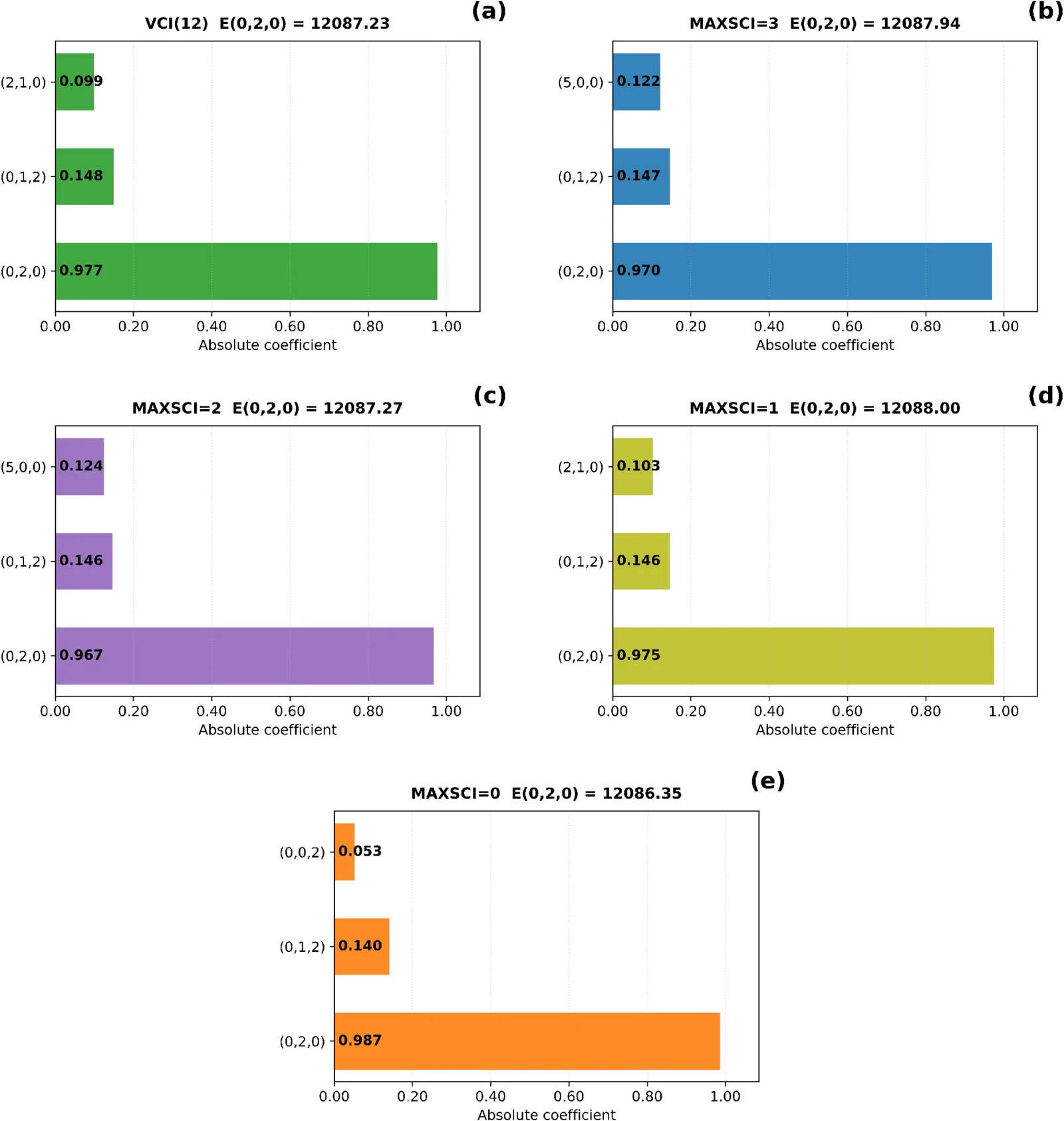
Absolute dominant configuration coefficients for the (0, 2, 0) state at a sampling cutoff of *N_q_* = 12. Panel (a) shows the full VCI(12) reference; panels (b–d) show the ViBra-enlarged spaces with MAXSCI = 1 to 3, respectively. Panel (e) shows direct diagonalization of the quantum-sampled seed with MAXSCI = 0.

This handoff points to (5,0,0) as the configuration responsible for the observed pattern, and tracking its energy directly explains why. At MAXSCI = 0, the root dominated by (5,0,0) (c = 0.988, essentially pure) lies at 12317.0 cm^–1^, roughly 230 cm^–1^ above (0,2,0): too far to interact appreciably. The energy of the (5,0,0) configuration decreases upon MAXSCI increments, and it becomes remarkably close to the (0,2,0) energy at MAXSCI = 2 and 3. At MAXSCI = 3, the (5,0,0)-dominated root drops sharply to 12066.5 cm^–1^, bringing it to about 21 cm^–1^ lower than (0,2,0); also, the (5,0,0) primary coefficient has already fallen to c = 0.896, showing that the state is no longer well described by a single configuration even at this intermediate active-space size. In the converged VCI(12) calculation, this near-degeneracy is fully resolved into a peak splitting: (5,0,0) no longer dominates a single root at all, but instead splits its character almost evenly between two roots, at 11517.5 cm^–1^ (c = 0.607) and 12565.0 cm^–1^ (c = 0.605), both of which also receive a substantial contribution from the (6,0,0) configuration, which only enters the active space once MAXSCI ≥ 1.

Note that (5,0,0) and (6,0,0) are both high-quanta configurations of water’s lowest-frequency mode, suggesting that properly describing this configuration requires including even higher-lying excited configurations as well. However, although the quantum-seed configurations were generated up to a maximum of *N*_*q*_ = 12 total quanta, only 11 of the 53 MAXSCI = 0 reference configurations carry six or more quanta. The (6,0,0) configuration itself is absent from that initial list and, like (2,1,0), only enters the active space once MAXSCI ≥ 1. On the other hand, only 42 configurations have total quanta of 5 or less, meaning that the low quanta lying states may be also under represented (due to the few configurations in total). This justifies why *N*_*q*_ = 12 exhibits some of the largest deviation for other configurations, particularly for the fundamental transitions.

Back to the (0,2,0) overtone, the small, nonmonotonic energy shift seen among MAXSCI = 0, MAXSCI = 3, and the converged VCI(12) result thus coincides precisely with the point at which its resonance partner sweeps into close energetic proximity, before the active space is large enough to fully resolve the resulting splitting of the (5,0,0) configuration. This highlights the importance of a well-constructed reference list. Because EN-PT2 selection operates relative to the fixed set of MAXSCI = 0 reference states, a configuration whose main interaction is with a state outside that list may only enter the active space indirectly—through its coupling to other reference states—rather than being targeted directly. Both (2,1,0) and (6,0,0) illustrate this: neither is present in the original 53-state list, yet both are already part of the active space by MAXSCI = 3. Their relevance to (0,2,0), however, differs in character. (2,1,0) mixes directly with (0,2,0): it is the configuration that eventually replaces (5,0,0) as the next-most-significant mixing partner of (0,2,0), after (0,1,2), in the converged VCI(12) calculation (c ≈ 0.10). (6,0,0) does not mix with (0,2,0) directly; instead, it mixes strongly with (5,0,0), which is itself the configuration responsible for the nonmonotonic convergence of (0,2,0) discussed above. (6,0,0) therefore needs to be well described not because it interacts with (0,2,0) directly, but because it shapes the (5,0,0) root that contributes to the (0,2,0) configuration. One refinement, not implemented or tested here but expected to be beneficial, would be to iterate the procedure: run a low-MAXSCI calculation, promote the resulting enlarged configuration set to serve as the new reference list, and repeat the EN-PT2 selection from there. Repeating this process would progressively approach the full VCI space, at the cost of the additional iterations and the larger subspaces they require diagonalizing.

Moreover, this example illustrates a broader point: while selected VCI can yield deceptively favorable energies at intermediate active-space sizes, it can also introduce spurious state mixing or apparent resonances that are artifacts of an incomplete space and, conversely, may under-represent genuine peak splittings where they should occur. We therefore emphasize the importance of inspecting wave function composition, peak intensities, and splittings alongside total energies when assessing convergence, rather than relying on energy deviations alone.

These results establish the intended role of ViBra in a hybrid quantum–classical vibrational workflow. The simulated quantum-facing routine supplies an initial set of vibrational configurations, $S_{Q}$ . With MAXSCI 0 list, ViBra constructs and diagonalizes the projected Hamiltonian directly in this externally sampled space. With MAXSCI > 0 in list-reference mode, the same sampled set is used as the reference for ViBra’s EN-PT2-guided configuration selection, producing a quantum-seeded but classically enlarged subspace. In both cases, the reported energies and eigenvectors are obtained through variational rediagonalization in the final configuration space.

The present H_2_O calculations are intended for workflow and interface validation. They show that quantum-generated seed configurations can be decoded, supplied to ViBra, and analyzed directly or systematically enlarged through its selected-VCI machinery. All quantum-facing calculations were performed using a simulator, and no execution on quantum hardware was undertaken. Accordingly, these results do not constitute a benchmark of SQD or SKQD, a study of shot complexity or scaling, or a claim of quantum advantage. Future work should examine configuration-recovery strategies,^30,47–49^ alternative SQD/SKQD sampling protocols, circuit and fragmentation optimization,^35^ alternative initial states, hardware execution, shot requirements, scaling with molecular size and spectral congestion, and the conditions under which a quantum advantage might eventually become possible.

## 7 Conclusions

The ViBra package delivers a complete, efficient, and user-friendly toolchain for anharmonic vibrational spectroscopy at the quartic force field level. By integrating configuration selection via S-VCI with symmetry exploitation via SA-VCI, it extends the reach of accurate VCI calculations to larger molecular systems without sacrificing physical rigor. The inclusion of second-order dipole derivatives guarantees reliable intensities across the full vibrational spectrum, while the accompanying GUI and viewer make the results immediately interpretable and directly comparable with experiment. The numerical correctness of ViBra was established through a four-tier benchmark. The VSCF and full VCI modules were validated against Crystal23 for water, achieving agreement within 0.001 cm^–1^ and 0.01 cm^–1^, respectively. The SA-VCI and S-VCI modules were validated against ViBra’s own full VCI calculations across a test set of five molecular point groups; SA-VCI reproduces full VCI to numerical precision, while S-VCI converges variationally to sub-5 cm^–1^ accuracy at substantially reduced cost. Taken together, these benchmarks confirm that ViBra is numerically correct and physically faithful.

The selected-VCI infrastructure in ViBra also provides a natural interface to quantum-centric algorithms for anharmonic vibrational spectroscopy. In such workflows, a quantum-facing routine supplies samples that define a compact seed space, while ViBra either analyzes that space directly or enlarges it through an EN-PT2-guided configuration selection before constructing and diagonalizing the projected Hamiltonian and evaluating transition observables. The H_2_O proof-of-concept validates this two-stage interface by combining qubit mapping, Hamiltonian fragmentation, and simulated time-evolution sampling with ViBra configuration-space refinement and projected diagonalization. This simulator-based demonstration does not constitute a hardware benchmark or imply quantum advantage, but it serves as motivation for ViBra and its capabilities as well as for the development of future quantum-centric workflows focused on vibrational problems.

In terms of implementation, there are several natural directions in which the current framework can be extended. Although the current focus is on quartic force fields, the code architecture is general, and higher-order expansions of the potential energy surface can be added straightforwardly. Similarly, the method can in principle be pushed toward still larger systems, although such applications have not yet been tested. On the algorithmic side, the current SA-VCI implementation is restricted to Abelian point groups with one-dimensional irreducible representations, and extending it to non-Abelian groups with degenerate irreps is planned for a future release.

Moreover, a key bottleneck in VCI calculations is the dramatic growth of the Hamiltonian matrix dimension with the number of modes and excitation quanta, which rapidly makes memory a limiting factor. Because the Hamiltonian matrix becomes increasingly sparse as the vibrational space grows, iterative diagonalizers that exploit this structure could reduce the memory footprint substantially. In fact, we have already implemented an iterative diagonalizer tailored to the sparsity pattern generated by our pair-list approach, and this is available for users. Additionally, we explored full dense matrix diagonalization via the DSYEVR and DSYEVD subroutines from Intel-LAPACK MKL, which we also retain as options owing to the outstanding performance of these routines. Further adaptation and refinement of our on-the-fly diagonalizer remain a natural next step.

## Supplementary Material





## Data Availability

The numerical data necessary to reproduce the validation tests, benchmark calculations, tables, and figures reported in this work are provided in a separate Zenodo data archive 10.5281/zenodo.22255923.^41^ The archive includes the quartic force-field inputs, ViBra input files, reference spectra, selected VCI and symmetry-adapted VCI benchmark outputs, timing data, selected configuration lists, and processed numerical data used to generate the figures and tables. Data associated with the exploratory H_2_O quantum-vibrational proof-of-concept calculation, including sampled vibrational configuration spaces and spectra generated from the external mapping, fragmentation, and time-evolution routines, are also included. Source code and analysis scripts are distributed separately. **Code availability:** The ViBra source code, documentation, and example input files are available at ref 50 (https://github.com/raphafe96/ViBra). The external proof-of-concept routines used for the quantum vibrational spectroscopy workflow, including the modal-to-qubit mapping, Pauli-operator construction, Hamiltonian fragmentation, Trotterized time-evolution, and simulator-based sampling scripts, are available at refs 45 and 46. The calculations reported in Section 6 were performed using the code versions identified by the repository release tags or commit hashes reported in the corresponding references.

## Notes

A comprehensive manual of ViBra, the detailed derivation of all equations, detailed descriptions of every Fortran module and Python class, a complete input-keyword reference, and an explanation of the output file formats (PDF)
(ZIP)
