# Supplementary material for: ViBra: Configuration Interaction for Anharmonic Vibrational Spectroscopy and Quantum-Sampled Configuration Spaces
Source: J Chem Theory Comput. 2026 Sep 28;22(21):11498–518. doi: 10.1021/acs.jctc.6c01564 (PMC13757753; doi:10.1021/acs.jctc.6c01564)
Supplement: Supplementary file 1 [file ct6c01564_si_001.pdf]

Raphael F. Ligorio[†], Marco A. Barroca[†], Alan Duriez[†], Mathias B. Steiner[‡]

[†] *Centro Brasileiro de Pesquisas Físicas (CBPF), 22290-180, Rio de Janeiro, RJ, Brasil*
[‡] *Instituto Kunumi, 30130-138, Belo Horizonte, MG, Brasil*

E-mail: raphafe96@cbpf.br ; mathias@kunumi.com

Supplementary Information

ViBra: Configuration Interaction for Anharmonic Vibrational Spectroscopy and Quantum-Sampled Configuration Spaces

This Supplementary Information comprises the program manual, including reference equations and example input and output files.

Code and Data Availability

Code repository: https://github.com/raphafe96/vibra

Data repository: https://doi.org/10.5281/zenodo.22255923

$\langle \Psi | V_i$Bra

Vibrational Structure Theory:

Vibrational Self Consistent Field (VSCF)
Vibrational Configuration Interaction (VCI)

Full, Selected and Symmetry Adapated VCI Methods @ the VSCF reference

Comprehensive Documentation of the
Physics, Models, and Implementation

Quartic Force Field · VSCF · VCI · Selected CI · Sym. Adap. CI · IR Intensities · GUI

Alpha V1.0

**Computational Engine:**	Fortran 90/95 with OpenMP and LAPACK
**Graphical Interface:**	Python 3 with CustomTkinter and Matplotlib
**Input:**	ORCA VPT2 output (`.vpt2`)

Centro Brasileiro de Pesquisas Físicas (CBPF)

September 2, 2026

Abstract This document describes the physics, formulations, and implementation of the ⟨Ψ| V_i**Bra** code for anharmonic vibrational spectroscopy. The Fortran 90/95 engine implements VSCF, full VCI, Selected VCI (Epstein–Nesbet PT2 screening) and Symmetry Adapted VCI on a quartic force field with OpenMP and BLAS/LAPACK. A Python 3 GUI manages input and execution; a Matplotlib-based viewer provides temperature-dependent spectra with experimental overlay and 3Dmol.js normal-mode animation. Input is exclusively from ORCA VPT2 files. HO and VSCF intensities use first-order dipole derivatives; VCI/SCI adds second-order dipole derivatives for overtone and combination band accuracy.

Contents

1 Introduction and Theoretical Background **S5**

1.1 Overview of $\langle\Psi|$ V_iBra . . . S5
1.2 The Vibrational Schrödinger Equation . . . S5
1.3 The Harmonic Approximation . . . S6
1.4 Hierarchy of Methods . . . S6

2 The Potential Energy Surface **S7**

2.1 Quartic Force Field Expansion . . . S7
2.2 Degeneracy Factors . . . S7
2.3 Dipole Derivatives: ORCA Format and Transformations . . . S7
2.3.1 First Derivatives (Cartesian → Normal) . . . S7
2.3.2 Second Derivatives (Mixed → Normal) . . . S8

3 Harmonic Oscillator Basis and Matrix Elements **S9**

4 Vibrational Self-Consistent Field (VSCF) **S10**

4.1 Motivation . . . S10
4.2 The VSCF Ansatz . . . S10
4.3 Separation of the Potential . . . S10
4.4 Derivation of the VSCF Equations . . . S11
4.5 The Effective One-Mode Hamiltonian . . . S11
4.6 Construction of the Mean-Field Coefficients $X_i^{(p)}$ . . . S11
4.7 Matrix Representation and Diagonalisation . . . S13
4.8 The Correlation Potential V_c . . . S13
4.9 Self-Consistent Iteration . . . S13
4.10 VSCF for Excited States . . . S14
4.11 VSCF Intensities (First-Order Dipole Only) . . . S14

5 Vibrational Configuration Interaction (VCI) **S16**

5.1 Motivation . . . S16
5.2 Choice of One-Mode Basis: VSCF Modals vs. Harmonic Oscillator . . . S16
5.3 The VCI Expansion: Using Ground-State VSCF Modals . . . S16
5.4 Relation to the Harmonic Oscillator Basis . . . S17
5.5 Precomputed Modal Integral Table . . . S17
5.6 Vectorised Potentials and Unique-Mode Metadata . . . S18
5.7 Sparse Pair List . . . S18
5.8 Inverted Index for Potential Terms . . . S18
5.9 Prefix–Suffix Overlap Products . . . S18
5.10 Hamiltonian Element Structure . . . S19
5.11 VCI Transition Dipoles (First + Second Order) . . . S19
5.12 Diagonalisation . . . S20

6 Selected Vibrational CI (SCI) **S21**

6.1 Motivation . . . S21
6.2 CISD Reference . . . S21
6.3 Epstein–Nesbet PT2 Screening . . . S21
6.4 Top-N Selection and Union . . . S21

7 Symmetry-Adapted Vibrational CI **S23**

7.1 Motivation . . . S23

7.2 Representation Theory Background . . . S23
7.2.1 Irreducible Representations and Character Tables . . . S23
7.2.2 Great Orthogonality Theorem and Reduction Formula . . . S23
7.2.3 Direct Products . . . S24
7.3 Assignment of Normal Modes to Irreducible Representations . . . S25
7.3.1 The $3N_{\text{at}}$-Dimensional Representation of Atomic Displacements . . . S25
7.3.2 Structure of the $3N_{\text{at}} \times 3N_{\text{at}}$ Representation Matrix . . . S26
7.3.3 Character of the $3N_{\text{at}}$-Dimensional Representation . . . S27
7.3.4 Character of a Single Normal Mode: the Full Permutation-Rotation Formula S27
7.3.5 Verification: Why the Formula Yields ± 1 . . . S28
7.3.6 Simplification for Unmoved Atoms . . . S28
7.3.7 Reference Frame Requirement . . . S29
7.3.8 Atom Permutation Table and the Projection Cutoff . . . S29
7.3.9 Irrep Identification . . . S30
7.3.10 Complete Example: Water in C_{2v} . . . S30
7.4 Symmetry of VCI Configurations . . . S31
7.4.1 Parity of One-Mode States . . . S31
7.4.2 Symmetry of a Multi-Mode Configuration . . . S31
7.5 Block-Diagonal Structure of the VCI Hamiltonian . . . S31
7.5.1 Vanishing of Cross-Symmetry Matrix Elements . . . S31
7.5.2 Block Structure and Diagonalisation . . . S32
7.6 Transition Dipoles and Infrared Intensities . . . S32
7.6.1 Symmetry Selection Rule . . . S32
7.6.2 Global Normalisation . . . S32
7.7 Diagnostic Output . . . S32
7.7.1 Principal Axis Frame . . . S33
7.7.2 Orientation Detection . . . S33
7.7.3 Atom Permutation Table . . . S33
7.7.4 Normal Mode Displacement Diagnostic . . . S33
7.7.5 Mode Assignment Summary . . . S34
7.8 Consequences of an Incorrect Group Assignment . . . S34
7.8.1 Case 1: The Assigned Group Is the True Group . . . S34
7.8.2 Case 2: The Assigned Group Contains Operations That Are Not Symmetry Operations of the Molecule . . . S34
7.8.3 Case 3: The Assigned Group Is a Subgroup of the True Group . . . S35
7.8.4 C_1: The Unconditionally Safe Choice . . . S36
7.8.5 Summary . . . S36
7.8.6 Diagnosis of an Incorrect Assignment . . . S36

8 Output File Formats S38
8.1 `vscf.out` — Complete Calculation Log . . . S38
8.1.1 VSCF Ground-State Block . . . S38
8.1.2 VSCF Excited-State Blocks . . . S38
8.1.3 VCI Configuration List . . . S38
8.1.4 SCI Selection Report . . . S39
8.1.5 VCI/SCI Eigenstate Table . . . S39
8.1.6 Timing . . . S39
8.2 `intensities.txt` . . . S39
8.3 `normal_mode.txt` . . . S40

9 Auxiliary Fortran Modules S41

10 Input Variables **S42**

11 Main Program Workflow **S43**

12 Graphical User Interface **S44**
12.1 Spectral Viewer and Normal-Mode Animator S44
12.2 Spectral Display S44
12.3 JCAMP-DX Overlay S44
12.3.1 Asymmetric Least Squares (ALS) Baseline Correction S44
12.3.2 Formulation S45
12.3.3 Parameters S45
12.4 VCI Transition Details S45
12.5 3D Normal-Mode Animation S45
12.6 Data Export S45

13 Computational Optimisation Strategies **S46**
13.1 Overlap Cache: Eliminating Redundant Basis Summations S46
13.2 Vectorised Potential Storage and Nonzero Flags S46
13.3 The Inverted Index: Why It Helps VCI but Not VSCF S47
13.3.1 VSCF: every term contributes to every mode S47
13.3.2 VCI: most terms are irrelevant for a given pair S47
13.4 Merged $\bar{V}_c$ and Mean-Field Passes S48
13.5 Sparse Pair List for VCI S49
13.6 Prefix–Suffix Overlap Products S49
13.7 OpenMP Parallelism S50
13.8 BLAS and LAPACK Usage S50
13.9 SCI as Computational Reduction S50
13.10 Symmetry-Adapted VCI as a Cost-Reduction Strategy S51
13.11 Memory Considerations S51

14 Summary and Outlook **S52**

1 Introduction and Theoretical Background

1.1 Overview of ⟨Ψ| V_iBra

⟨Ψ| V_i**Bra** is a software package for computing anharmonic vibrational energies, wavefunctions, and infrared absorption spectra of polyatomic molecules. It has two components:

1. A **computational engine** in **Fortran 90/95** implementing the Vibrational Self-Consistent Field (VSCF), Vibrational Configuration Interaction (VCI), and Selected Vibrational Configuration Interaction (SCI) methods, linked to LAPACK/BLAS and parallelised with OpenMP.

2. A **graphical user interface** and **spectral viewer** in **Python 3**, using CustomTkinter for the interface, Matplotlib for plotting, and 3Dmol.js for 3D normal-mode animation in a web browser.

Input Requirement

⟨Ψ| V_i**Bra** requires the harmonic Hessian, cubic and quartic force constants, and first and second dipole moment derivatives, all provided *exclusively* in the **ORCA VPT2 output format** (`.vpt2`). No other electronic structure output is supported.

The Fortran engine writes three output files consumed by the visualisation layer:

- `vscf.out` — complete log: VSCF convergence, modal coefficients, VCI/SCI eigenstates, wavefunction composition.
- `intensities.txt` — transition frequencies and normalised IR intensities for HO, VSCF, and VCI.
- `normal_mode.txt` — molecular geometry and displacement vectors for all $3N_{\text{at}}$ modes.

Dipole Approximation Levels

HO and **VSCF** use only **first-order dipole derivatives** (linear dipole surface). Only **VCI** and **SCI** include **second-order dipole derivatives** $\partial^2\mu_\alpha/\partial Q_i \partial Q_j$, which are critical for overtone and combination band intensities and for the correct redistribution of intensity through Fermi resonances.

1.2 The Vibrational Schrödinger Equation

Within the Born–Oppenheimer approximation, nuclear vibrations are governed by:

$$\hat{H}_{\text{vib}}\,\Psi(\mathbf{Q}) = E\,\Psi(\mathbf{Q}) \tag{1}$$

where $\mathbf{Q} = (Q_1, \ldots, Q_M)$ are mass-weighted normal coordinates. For a nonlinear molecule with N_{at} atoms, $M = 3N_{\text{at}} - 6$; for linear, $M = 3N_{\text{at}} - 5$.

Normal coordinates are defined by diagonalising the mass-weighted Hessian:

$$F_{ij}^{\text{mw}} = \frac{F_{ij}}{\sqrt{m_a\, m_b}}, \qquad a = \lceil i/3 \rceil,\ b = \lceil j/3 \rceil \tag{2}$$

where F_{ij} is the Cartesian Hessian and m_a is the mass of atom a. The eigenvalues give ω_i^2; the eigenvectors define the Cartesian-to-normal-coordinate transformation.

The full Watson Hamiltonian [1] includes rovibrational coupling, Coriolis terms, and a mass-dependent "extra potential." ⟨Ψ| V_i**Bra** neglects all of these (valid for semi-rigid molecules at $J = 0$), giving:

$$\hat{H}_{\text{vib}} = -\frac{1}{2}\sum_{i=1}^{M}\frac{\partial^2}{\partial Q_i^2} + V(\mathbf{Q}) \tag{3}$$

in atomic units. The kinetic energy is diagonal in normal coordinates; all coupling enters through the potential.

1.3 The Harmonic Approximation

At the harmonic level, $V^{(2)} = \frac{1}{2}\sum_i \omega_i^2 Q_i^2$, and the Hamiltonian separates into independent oscillators with eigenvalues $\varepsilon_{n_i}^{(0)} = \omega_i(n_i + \frac{1}{2})$ and Hartree product wavefunctions $\Psi_{\mathbf{n}}^{(0)} = \prod_i \phi_{n_i}(Q_i)$.

Limitations: anharmonic frequency shifts are neglected; overtones and combination bands have zero intensity in the double-harmonic approximation; Fermi resonances cannot be described; all mode–mode coupling is absent.

1.4 Hierarchy of Methods

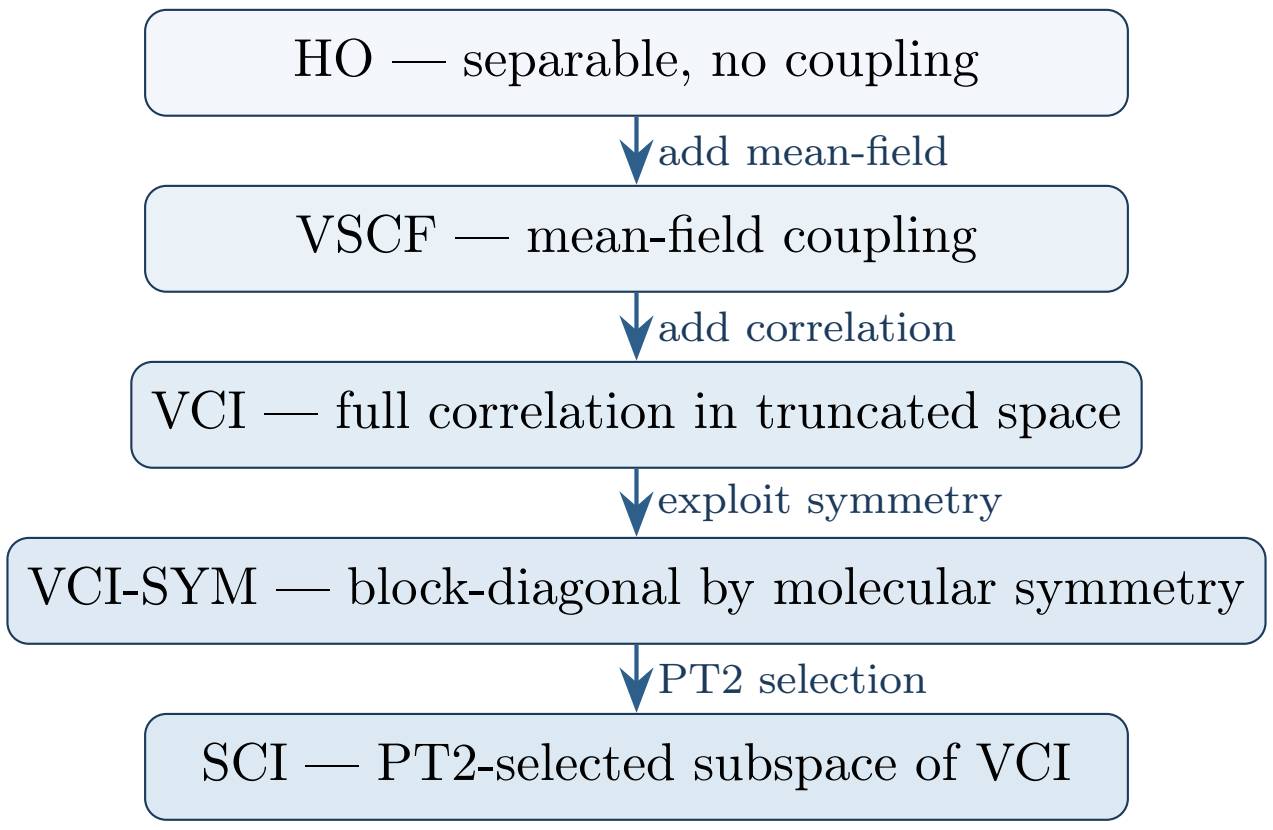

2 The Potential Energy Surface

2.1 Quartic Force Field Expansion

The PES is expanded about equilibrium at fourth order:

$$V(\mathbf{Q}) = \frac{1}{2}\sum_i \omega_i^2 Q_i^2 + \frac{1}{6}\sum_{ijk} \phi_{ijk}\, Q_i Q_j Q_k + \frac{1}{24}\sum_{ijkl} \phi_{ijkl}\, Q_i Q_j Q_k Q_l \tag{4}$$

where ϕ_{ijk} and ϕ_{ijkl} are the cubic and quartic force constants. These tensors are totally symmetric under index permutation. Only the upper triangle is stored:

$$N_3 = \binom{M+2}{3}, \qquad N_4 = \binom{M+3}{4} \tag{5}$$

The cubic terms describe three-mode coupling: diagonal ($i = j = k$) terms are the leading anharmonic correction to a single mode, while terms with distinct indices represent genuine multi-mode interactions. Quartic terms follow analogously with up to four-body coupling.

2.2 Degeneracy Factors

Each stored term carries its permutational weight $g = n!/\prod_s(n_s!)$:

Table S1: Degeneracy factors $g = n!/\prod_s(n_s!)$ for the stored upper-triangular force constants.

**Cubic pattern** ($i \le j \le k$)	**Example**	g
All same ($i = j = k$)	ϕ_{111}	1
Two equal + one	ϕ_{112}, ϕ_{122}	3
All different	ϕ_{123}	6

**Quartic pattern** ($i \le j \le k \le l$)	**Example**	g
All same ($i = j = k = l$)	ϕ_{1111}	1
Triple + single	ϕ_{1112}	4
Two doubles	ϕ_{1122}	6
Double + two singles	ϕ_{1123}	12
All different	ϕ_{1234}	24

The subroutine `apply_degeneracy_factors` first symmetrises the tensor (propagating nonzero values to all index permutations), then zeroes the full array and refills only the upper triangle with the weighted values.

2.3 Dipole Derivatives: ORCA Format and Transformations

ORCA provides dipole derivatives in two different representations, requiring different transformations to reach the fully normal-coordinate form needed by ⟨Ψ| V_iBra.

2.3.1 First Derivatives (Cartesian → Normal)

The first dipole derivatives are provided by ORCA entirely in Cartesian coordinates:

$$\left(\frac{\partial \mu_\alpha}{\partial x_k}\right)_0, \qquad \alpha \in \{x, y, z\}, \quad k = 1, \ldots, 3N_{\text{at}} \tag{6}$$

Since Cartesian displacements relate to normal coordinates via $\partial x_k/\partial Q_i = L_{ki}/\sqrt{m_a}$ (where L_{ki} is the mass-weighted Hessian eigenvector and $a = \lceil k/3 \rceil$), the full transformation is:

$$\boxed{\frac{\partial \mu_\alpha}{\partial Q_i} = \sum_{k=1}^{3N_\text{at}} \frac{\partial \mu_\alpha}{\partial x_k} \frac{L_{ki}}{\sqrt{m_a}}} \tag{7}$$

This is a matrix–matrix product of the Cartesian dipole derivative matrix ($3N_\text{at} \times 3$) with the mass-weighted eigenvector matrix, yielding a ($3N_\text{at} \times 3$) result from which only the M vibrational rows are retained (after skipping the translational/rotational modes: the first 6 for nonlinear, 5 for linear molecules).

2.3.2 Second Derivatives (Mixed → Normal)

The second dipole derivatives are provided by ORCA in a *mixed* representation: the first index is already a vibrational normal mode Q_i, but the second index remains a Cartesian coordinate x_k:

$$\frac{\partial^2 \mu_\alpha}{\partial Q_i\, \partial x_k} \tag{8}$$

Only the second (Cartesian) index needs to be transformed. Applying the same relation $\partial x_k/\partial Q_j = L_{kj}/\sqrt{m_a}$:

$$\boxed{\frac{\partial^2 \mu_\alpha}{\partial Q_i\, \partial Q_j} = \sum_{k=1}^{3N_\text{at}} \frac{\partial^2 \mu_\alpha}{\partial Q_i\, \partial x_k} \frac{L_{kj}}{\sqrt{m_a}}} \tag{9}$$

The ORCA-provided array has dimensions $M \times 3N_\text{at} \times 3$ (mode i, Cartesian k, dipole component α). After the transformation, the result is $M \times M \times 3$ (mode i, mode j, component α), again restricted to vibrational modes only.

Factor of $1/2$ convention

In the main program, the transformed second derivatives are divided by 2 before being passed to the VCI routine. This absorbs the $1/2$ from the Taylor expansion $\hat{\mu}_\alpha \approx \mu_\alpha^{(0)} + \sum_i \mu_\alpha^{(i)} Q_i + \frac{1}{2} \sum_{ij} \mu_\alpha^{(ij)} Q_i Q_j$, so that the VCI dipole routine multiplies directly by the stored value without an additional prefactor.

3 Harmonic Oscillator Basis and Matrix Elements

The 1D harmonic oscillator with frequency ω has eigenfunctions $\phi_n(Q)$ and ladder operators satisfying $Q = (\hat{a}^\dagger + \hat{a})/\sqrt{2\omega}$. The `compute_integrals` module computes $\langle m|Q^p|n\rangle$ analytically for $p = 0, \ldots, 4$ and the kinetic energy for $p = 5$. Note that the power fork kinetic energy is NOT 5, but the index 5 was used for looping.

$p = 0$ **(overlap):** $\langle m|n\rangle = \delta_{mn}$

$p = 1$ **(position):**

$$\langle m|Q|n\rangle = \sqrt{\frac{n+1}{2\omega}}\,\delta_{m,n+1} + \sqrt{\frac{n}{2\omega}}\,\delta_{m,n-1} \tag{10}$$

$p = 2$**:**

$$\langle m|Q^2|n\rangle = \frac{n+\frac{1}{2}}{\omega}\,\delta_{mn} + \frac{\sqrt{(n+1)(n+2)}}{2\omega}\,\delta_{m,n+2} + \frac{\sqrt{n(n-1)}}{2\omega}\,\delta_{m,n-2} \tag{11}$$

$p = 3$**:**

$$\begin{aligned}\langle m|Q^3|n\rangle = {}& \frac{3(n+1)\sqrt{n+1}}{2\omega\sqrt{2\omega}}\,\delta_{m,n+1} + \frac{3n\sqrt{n}}{2\omega\sqrt{2\omega}}\,\delta_{m,n-1} \\ &+ \frac{\sqrt{(n+1)(n+2)(n+3)}}{2\omega\sqrt{2\omega}}\,\delta_{m,n+3} + \frac{\sqrt{n(n-1)(n-2)}}{2\omega\sqrt{2\omega}}\,\delta_{m,n-3}\end{aligned} \tag{12}$$

$p = 4$**:**

$$\begin{aligned}\langle m|Q^4|n\rangle = {}& \frac{6n(n+1)+3}{4\omega^2}\,\delta_{mn} + \frac{(2n+3)\sqrt{(n+1)(n+2)}}{2\omega^2}\,\delta_{m,n+2} \\ &+ \frac{(2n-1)\sqrt{n(n-1)}}{2\omega^2}\,\delta_{m,n-2} \\ &+ \frac{\sqrt{(n+1)(n+2)(n+3)(n+4)}}{4\omega^2}\,\delta_{m,n+4} \\ &+ \frac{\sqrt{n(n-1)(n-2)(n-3)}}{4\omega^2}\,\delta_{m,n-4}\end{aligned} \tag{13}$$

$p = 5$ **(kinetic energy):** $\hat{T}_i = -\frac{1}{2}\,\partial^2/\partial Q_i^2$:

$$\langle m|\hat{T}_i|n\rangle = \frac{\omega(n+\frac{1}{2})}{2}\,\delta_{mn} - \frac{\omega\sqrt{(n+1)(n+2)}}{4}\,\delta_{m,n+2} - \frac{\omega\sqrt{n(n-1)}}{4}\,\delta_{m,n-2} \tag{14}$$

Selection rule: $\langle m|Q^p|n\rangle \neq 0$ only when $|m-n| \leq p$. For a quartic force field, two VCI configurations can couple only if they differ in at most 4 modes.

4 Vibrational Self-Consistent Field (VSCF)

4.1 Motivation

The exact vibrational wavefunction $\Psi(Q_1, \ldots, Q_M)$ depends on all M coordinates simultaneously. Representing it on a direct product grid requires $\sim N_b^M$ points, which grows exponentially with M and becomes intractable for more than a handful of modes. Even expanding in a CI basis (Section 5) is costly because the number of configurations grows combinatorially.

The VSCF approach, introduced independently by Bowman [2] and Gerber and Ratner [3], addresses this by approximating the full M-dimensional problem as M coupled *one-dimensional* problems. Each mode "sees" the other modes only through their average (mean-field) effect—exactly the vibrational analogue of the Hartree–Fock method in electronic structure theory. The computational cost is reduced from exponential in M to linear: M diagonalisations of small $N_{\text{exp}} \times N_{\text{exp}}$ matrices per iteration, where N_{exp} is the number of basis functions per mode.

4.2 The VSCF Ansatz

The VSCF wavefunction is a Hartree product of one-mode functions called *modals*:

$$\boxed{\Psi_{\mathbf{n}}(\mathbf{Q}) = \prod_{i=1}^{M} \varphi_{n_i}^{(i)}(Q_i)} \tag{15}$$

where $\mathbf{n} = (n_1, n_2, \ldots, n_M)$ specifies the quantum state of each mode. Each modal is expanded in the harmonic oscillator (HO) basis:

$$\varphi_{n_i}^{(i)}(Q_i) = \sum_{\mu=1}^{N_{\text{exp}}} C_{\mu,n_i}^{(i)} \, \phi_{\mu-1}(Q_i) \tag{16}$$

The expansion coefficients $C_{\mu,n_i}^{(i)}$ are the variational parameters to be optimised. The number of basis functions per mode N_{exp} is set by the input parameter `NEXPAN`; it determines the flexibility of the modals and hence the quality of the mean-field approximation.

4.3 Separation of the Potential

It is useful to partition the full potential into *diagonal* (single-mode) and *coupling* (multi-mode) parts:

$$V(\mathbf{Q}) = \sum_{i=1}^{M} V_i^{\text{diag}}(Q_i) + V_c(Q_1, \ldots, Q_M) \tag{17}$$

The diagonal potential for mode i collects *every* term that depends on Q_i alone—harmonic *and* anharmonic:

$$V_i^{\text{diag}}(Q_i) = \tfrac{1}{2}\omega_i^2 \, Q_i^2 + \tilde{\phi}_{iii} \, Q_i^3 + \tilde{\phi}_{iiii} \, Q_i^4 \tag{18}$$

The coupling potential V_c contains all terms in which **at least two distinct mode indices** appear:

$$V_c(\mathbf{Q}) = \sum_{i<j\leq k} \tilde{\phi}_{ijk} \, Q_i \, Q_j \, Q_k \; + \sum_{i<j\leq k\leq l} \tilde{\phi}_{ijkl} \, Q_i \, Q_j \, Q_k \, Q_l \tag{19}$$

where the sums run over the upper-triangular unique index sets. The Taylor expansion and symmetry prefactors are absorbed into $\tilde{\phi}_{\ldots}$ as defined in Section 2. The key point is that purely diagonal anharmonic terms such as $\tilde{\phi}_{iii} \, Q_i^3$ and $\tilde{\phi}_{iiii} \, Q_i^4$ are *not* part of V_c; they belong to $V_i^{\text{diag}}(Q_i)$ and are treated exactly within the one-mode problem.

4.4 Derivation of the VSCF Equations

The VSCF energy functional is the expectation value of the full Hamiltonian over the Hartree product:

$$E_{\mathbf{n}} = \langle \Psi_{\mathbf{n}} | \hat{H}_{\text{vib}} | \Psi_{\mathbf{n}} \rangle \tag{20}$$

We seek the modals that make $E_{\mathbf{n}}$ stationary with respect to variations in each $\varphi_{n_i}^{(i)}$, subject to the normalisation constraint $\langle \varphi_{n_i}^{(i)} | \varphi_{n_i}^{(i)} \rangle = 1$. Introducing a Lagrange multiplier $\varepsilon_{n_i}^{(i)}$ for each mode and setting the functional derivative to zero:

$$\frac{\delta}{\delta \varphi_{n_i}^{(i)*}} \left[E_{\mathbf{n}} - \varepsilon_{n_i}^{(i)} \left(\langle \varphi_{n_i}^{(i)} | \varphi_{n_i}^{(i)} \rangle - 1 \right) \right] = 0 \tag{21}$$

yields the M coupled eigenvalue problems:

$$\boxed{\hat{h}_i^{\text{eff}} \; \varphi_{n_i}^{(i)}(Q_i) = \varepsilon_{n_i}^{(i)} \; \varphi_{n_i}^{(i)}(Q_i), \qquad i = 1, \ldots, M} \tag{22}$$

The Lagrange multiplier $\varepsilon_{n_i}^{(i)}$ is the *modal energy* of mode i in state n_i—the energy eigenvalue of the effective one-mode problem. Equation (22) states that each modal is an eigenfunction of its own effective Hamiltonian, which depends on all other modals through the mean-field potential.

4.5 The Effective One-Mode Hamiltonian

The effective Hamiltonian for mode i collects the kinetic energy of mode i, the full diagonal potential of mode i (harmonic *and* anharmonic), and the mean-field average of the coupling potential over all other modes:

$$\hat{h}_i^{\text{eff}} = \underbrace{-\frac{1}{2}\frac{\partial^2}{\partial Q_i^2}}_{\hat{T}_i} + \underbrace{V_i^{\text{diag}}(Q_i)}_{\text{diagonal (all orders)}} + \underbrace{\langle V_c \rangle_{j \neq i}}_{\text{mean-field coupling}} \tag{23}$$

The mean-field coupling potential is obtained by integrating the coupling potential V_c over the current modals of all modes except i:

$$\langle V_c \rangle_{j \neq i} = \left\langle \prod_{j \neq i} \varphi_{n_j}^{(j)} \middle| V_c(\mathbf{Q}) \middle| \prod_{j \neq i} \varphi_{n_j}^{(j)} \right\rangle \tag{24}$$

Because the coupling potential is a polynomial in the normal coordinates (up to fourth order in the quartic force field), and the integration is performed analytically over the modals of modes $j \neq i$, the resulting mean-field coupling potential for mode i is itself a polynomial in Q_i. Combining it with the diagonal potential, the full effective Hamiltonian takes the form:

$$\boxed{\hat{h}_i^{\text{eff}} = \hat{T}_i + \sum_{p=0}^{4} X_i^{(p)} \, Q_i^p} \tag{25}$$

The coefficients $X_i^{(p)}$ are real numbers that depend on the force constants and on the current modals of all other modes (and hence change at each VSCF iteration). They receive contributions from both the diagonal potential and the mean-field coupling potential, as described below.

4.6 Construction of the Mean-Field Coefficients $X_i^{(p)}$

How $X_i^{(p)}$ is built from force constants

Each term in the potential contributes to $X_i^{(p)}$ according to how many powers of Q_i it contains. Call this multiplicity q: the term contributes Q_i^q as the operator acting on mode i, while the remaining Q_j factors (for modes $j \neq i$) are averaged over the current modals to produce a scalar.

Diagonal contributions (no averaging needed):

- The harmonic term $\frac{1}{2}\omega_i^2\, Q_i^2$ contributes directly to $X_i^{(2)}$.
- The diagonal cubic $\tilde{\phi}_{iii}\, Q_i^3$ contributes directly to $X_i^{(3)}$.
- The diagonal quartic $\tilde{\phi}_{iiii}\, Q_i^4$ contributes directly to $X_i^{(4)}$.

Coupling contributions (modes $j \neq i$ are averaged):

Cubic example: Consider $\tilde{\phi}_{113}\, Q_1^2 Q_3$ with working mode $i = 1$. Mode 1 appears with multiplicity $q = 2$ (two powers of Q_1), and mode 3 appears with multiplicity 1. The Q_1^2 part becomes the operator; the Q_3 part is averaged:

$$\Delta X_1^{(2)} \mathrel{+}= \tilde{\phi}_{113}\, \langle \varphi_{n_3}^{(3)} | Q_3 | \varphi_{n_3}^{(3)} \rangle \tag{26}$$

Quartic example: Consider $\tilde{\phi}_{1223}\, Q_1 Q_2^2 Q_3$ with working mode $i = 1$. Mode 1 appears once ($q = 1$); modes 2 and 3 are averaged:

$$\Delta X_1^{(1)} \mathrel{+}= \tilde{\phi}_{1223}\, \langle \varphi_{n_2}^{(2)} | Q_2^2 | \varphi_{n_2}^{(2)} \rangle\, \langle \varphi_{n_3}^{(3)} | Q_3 | \varphi_{n_3}^{(3)} \rangle \tag{27}$$

Quartic, two powers on working mode: Consider $\tilde{\phi}_{1123}\, Q_1^2 Q_2 Q_3$ with $i = 1$ ($q = 2$):

$$\Delta X_1^{(2)} \mathrel{+}= \tilde{\phi}_{1123}\, \langle \varphi_{n_2}^{(2)} | Q_2 | \varphi_{n_2}^{(2)} \rangle\, \langle \varphi_{n_3}^{(3)} | Q_3 | \varphi_{n_3}^{(3)} \rangle \tag{28}$$

Coupling terms where mode i does not appear ($q = 0$): some coupling terms in V_c involve only modes $j \neq i$. When averaged over *all* modes $j \neq i$, these produce a pure scalar that shifts $X_i^{(0)}$:

$$\Delta X_i^{(0)} \mathrel{+}= \tilde{\phi}_{jk\cdots} \prod_{s \in \{j,k,\ldots\}} \langle \varphi_{n_s}^{(s)} | Q_s^{c_s} | \varphi_{n_s}^{(s)} \rangle \tag{29}$$

These same terms also contribute to the correlation potential V_c discussed in Section 4.8.

In general, each expectation value $\langle \varphi_{n_s}^{(s)} | Q_s^{c_s} | \varphi_{n_s}^{(s)} \rangle$ is computed from the current modal coefficients $C_{\mu,n_s}^{(s)}$ and the precomputed primitive integrals in `store_integrals`:

$$\langle \varphi_{n_s}^{(s)} | Q_s^{c_s} | \varphi_{n_s}^{(s)} \rangle = \sum_{\mu=1}^{N_{\exp}} \sum_{\nu=1}^{N_{\exp}} C_{\mu,n_s}^{(s)}\, C_{\nu,n_s}^{(s)}\, \langle \phi_{\mu-1} | Q_s^{c_s} | \phi_{\nu-1} \rangle \tag{30}$$

4.7 Matrix Representation and Diagonalisation

Substituting the modal expansion $\varphi_{n_i}^{(i)} = \sum_\mu C_{\mu,n_i}^{(i)} \phi_{\mu-1}$ into Eq. (22) and projecting onto $\langle\phi_{\nu-1}|$ gives the matrix eigenvalue problem:

$$\boxed{\sum_{\mu=1}^{N_{\mathrm{exp}}} H_{\nu\mu}^{(i)}\, C_{\mu,n_i}^{(i)} = \varepsilon_{n_i}^{(i)}\, C_{\nu,n_i}^{(i)}} \tag{31}$$

where the matrix elements of the effective Hamiltonian are:

$$H_{\nu\mu}^{(i)} = \langle\phi_{\nu-1}|\hat{T}_i|\phi_{\mu-1}\rangle + \sum_{p=0}^{4} X_i^{(p)}\, \langle\phi_{\nu-1}|Q_i^p|\phi_{\mu-1}\rangle \tag{32}$$

Each term in this sum is a product of the coefficient $X_i^{(p)}$ (a scalar computed from the force constants and the current modals of all other modes) and a primitive HO matrix element $\langle\phi_{\nu-1}|Q_i^p|\phi_{\mu-1}\rangle$ (precomputed analytically and stored in `store_integrals`). The matrix $\mathbf{H}^{(i)}$ is real symmetric and of size $N_{\mathrm{exp}} \times N_{\mathrm{exp}}$; it is diagonalised by LAPACK's `DSYEVD`.

Diagonalisation yields N_{exp} eigenvalues and eigenvectors. The n_i-th eigenvalue is the modal energy $\varepsilon_{n_i}^{(i)}$, and the corresponding eigenvector gives the updated expansion coefficients $C_{\mu,n_i}^{(i)}$.

4.8 The Correlation Potential V_c

The fully averaged coupling potential $\bar{V}_c$ is the expectation value of the coupling potential V_c (Eq. 19) where *every* mode is integrated out—no mode is left as an operator:

$$\bar{V}_c = \left\langle \prod_{i=1}^{M} \varphi_{n_i}^{(i)} \middle| V_c(\mathbf{Q}) \middle| \prod_{i=1}^{M} \varphi_{n_i}^{(i)} \right\rangle \tag{33}$$

This quantity appears because the effective Hamiltonian for mode i includes the mean-field average of *all* coupling terms—even those that couple only modes $j, k \neq i$ among themselves. When we sum the modal energies $\sum_i \varepsilon_{n_i}^{(i)}$, each coupling term is counted multiple times (once in each mode's effective Hamiltonian that includes it in the mean field), whereas it should appear only once in the total energy. The corrected VSCF energy is therefore:

$$\boxed{E_{\mathrm{VSCF}} = \sum_{i=1}^{M} \varepsilon_{n_i}^{(i)} - (M-1)\,\bar{V}_c} \tag{34}$$

To see why the factor is $(M-1)$: each coupling term in V_c appears in the effective Hamiltonian of *every* mode (either as an operator piece when that mode is involved, or as a scalar shift $X_i^{(0)}$ when it is not). Summing over all M modes therefore counts $\bar{V}_c$ a total of M times. Subtracting $(M-1)\bar{V}_c$ leaves $\bar{V}_c$ counted exactly once.

4.9 Self-Consistent Iteration

Since the effective Hamiltonian $\hat{h}_i^{\mathrm{eff}}$ for mode i depends on the modals of all modes $j \neq i$ (through the coefficients $X_i^{(p)}$), the VSCF equations (22) must be solved iteratively.

Algorithm 1 VSCF self-consistent field iteration.

Initialise coefficients $C^{(i)}_{\mu,n_i} \leftarrow$ small uniform values for all modes
repeat
  $E_{\text{old}} \leftarrow E_{\text{VSCF}}$
  for each mode $i = 1, \dots, M$ **do**
    Compute $X^{(p)}_i$ $(p = 0, \dots, 4)$ from diagonal potential and mean-field coupling using current modals of all modes $j \neq i$
    Build $H^{(i)}_{\nu\mu}$ and diagonalise (`DSYEVD`)
    Update $C^{(i)}_{\mu,n_i}$ from the n_i-th eigenvector
  end for
  Compute $\bar{V}_c$ from the fully averaged coupling potential
  $E_{\text{VSCF}} \leftarrow \sum_i \varepsilon^{(i)}_{n_i} - (M-1)\bar{V}_c$
until $|E_{\text{VSCF}} - E_{\text{old}}| < 10^{-\texttt{CVGSCF}}$ cm^{-1}

The convergence threshold is controlled by the input parameter `CVGSCF`: convergence is declared when the energy change between successive iterations falls below $10^{-\texttt{CVGSCF}}$ cm^{-1}.

The main program first converges the ground state $\mathbf{n} = (0, \dots, 0)$, storing the full modal coefficient matrix `full_coef(mode, state, basis)` for all modes and all N_{exp} eigenstates. This matrix defines the VSCF modal basis used subsequently by the VCI and SCI calculations. Then each fundamental excitation $\mathbf{n} = \mathbf{e}_i$ is independently converged with its own set of modals.

4.10 VSCF for Excited States

For each fundamental excitation of mode i (i.e. $\mathbf{n} = (0, \dots, 1_i, \dots, 0)$), the VSCF iteration is restarted from scratch with fresh initial coefficients. During the iteration, the effective Hamiltonian for mode i is built with $n_i = 1$ (the first excited state), and the modal energy extracted is $\varepsilon^{(i)}_1$. The modals of all other modes $j \neq i$ are optimised for $n_j = 0$.

The VSCF transition energy for mode i is:

$$\Delta E^{\text{VSCF}}_i = E_{\text{VSCF}}(\mathbf{e}_i) - E_{\text{VSCF}}(\mathbf{0}) \tag{35}$$

This is printed alongside the harmonic frequency ω_i for direct comparison of the anharmonic shift.

4.11 VSCF Intensities (First-Order Dipole Only)

VSCF transition dipole with state-specific modals

The transition dipole for a fundamental $\mathbf{0} \to \mathbf{e}_i$ is evaluated using VSCF modals optimised separately for the ground and the excited vibrational states. The ground-state modals are denoted $\varphi^{(j)}_0$; the excited-state modals are denoted $\varphi^{(j)}_{n_j}$. To avoid confusion, the program performs independent VSCF calculations for the ground state and for the given excited state. In these two VSCF routines, the eigenvector retained for each mode corresponds to the ground vibrational level for the ground-state calculation, and to the target excited level for the excited-state calculation. This means that, in the end, the modals are optimised in different mean fields. These sets of modals, from the different VSCF calculations, are *not* assumed to be orthogonal, and their mutual overlaps are computed explicitly. The transition dipole is then given by

$$\langle \Psi_{\mathbf{0}} | \hat{\mu}_\alpha | \Psi_{\mathbf{e}_i} \rangle = \sum_{j=1}^{M} \frac{\partial \mu_\alpha}{\partial Q_j} \langle \varphi^{(j)}_0 | Q_j | \varphi^{(j)}_{n_j} \rangle \prod_{k \neq j} \langle \varphi^{(k)}_0 | \varphi^{(k)}_{n_k} \rangle. \tag{36}$$

The IR intensity is obtained from

$$I_i^{\mathrm{VSCF}} \propto \sum_{\alpha=x,y,z} \left|\langle \Psi_{\mathbf{0}} | \hat{\mu}_\alpha | \Psi_{\mathbf{e}_i} \rangle\right|^2 . \tag{37}$$

Note that the permanent dipole contribution $\mu_{0,\alpha}\langle \Psi_{\mathbf{0}} | \Psi_{\mathbf{e}_i} \rangle$ is not included. For exact, mutually orthogonal vibrational eigenstates this term vanishes identically; here, however, $\Psi_{\mathbf{0}}$ and $\Psi_{\mathbf{e}_i}$ are built from independently optimised, non-orthogonal modal sets, so its omission is an approximation rather than a rigorous cancellation. For this reason, and because the scheme above is evaluated only for fundamental transitions, the resulting I_i^{VSCF} should be regarded as a practical estimate of the IR intensity rather than an exact transition dipole moment, even though it is typically found to be reasonably accurate.

5 Vibrational Configuration Interaction (VCI)

5.1 Motivation

VSCF captures the *average* effect of coupling but misses *correlation*—the correlated response of multiple modes. VCI recovers this by expanding the wavefunction as a linear combination of many Hartree products (configurations), analogous to the transition from Hartree–Fock to CI in electronic structure theory.

5.2 Choice of One-Mode Basis: VSCF Modals vs. Harmonic Oscillator

In vibrational CI calculations, one must choose a one-mode basis in which to expand the multi-mode wavefunction. Two common choices exist:

Harmonic oscillator (HO) basis: Many VCI codes use the primitive harmonic oscillator eigenfunctions $\phi_n(Q_i)$ directly as the one-mode basis. In this approach, the CI configurations are simply products of HO functions: $|\Phi_{\mathbf{n}}^{\text{HO}}\rangle = \prod_i \phi_{n_i}(Q_i)$. This choice is computationally straightforward because all matrix elements are known analytically (Section 3), and no preliminary VSCF calculation is required.

VSCF modal basis (used by ⟨Ψ| V_iBra): As described below, ⟨Ψ| V_iBra first solves the VSCF equations to obtain optimised one-mode functions $\varphi_v^{(i)}(Q_i)$ (modals) that incorporate the mean-field effect of mode–mode coupling. These modals then serve as the one-mode basis for the VCI expansion.

Trade-off: HO basis vs. VSCF modal basis

The HO basis converges slowly with respect to the maximum quanta N_q. Because the HO functions take no coupling into account, many HO configurations (including those with high quanta) are needed to represent anharmonic and coupled vibrational states accurately. Consequently, HO-based VCI often requires $N_q = 6$–10 or even higher to achieve convergence, leading to extremely large configuration spaces.

The VSCF modal basis, by contrast, already contains mean-field coupling information. Each modal $\varphi_v^{(i)}$ is adapted to the effective potential experienced by mode i in the presence of all other modes. As a result, the VCI expansion converges much more rapidly with respect to N_q: typically $N_q = 4$ suffices for accurate fundamental and overtone frequencies. The trade-off is the additional computational cost of the VSCF ground-state calculation (which is cheap, scaling linearly with M) and the precomputation of the modal integral table.

In summary:

	HO basis	VSCF modal basis
Convergence rate	Slow	Faster
Typical N_q needed	6–10	4–5
Configuration space size	Very large	Smaller
Precomputation cost	None	VSCF + integral table (fast)

5.3 The VCI Expansion: Using Ground-State VSCF Modals

A critical design choice in ⟨Ψ| V_iBra is that the one-mode functions (modals) used to build the VCI configurations are taken exclusively from the **converged VSCF ground-state calculation** ($\mathbf{n} = \mathbf{0}$), not from state-specific VSCF runs for excited states. This means:

- For each mode i, we have a set of orthonormal modals $\{\varphi_v^{(i)}\}_{v=0}^{N_{\text{exp}}-1}$ obtained by diagonalising the ground-state effective Hamiltonian $\hat{h}_i^{\text{eff}}$ (Section 4).
- These modals are the eigenvectors of the same one-mode Hamiltonian, so they satisfy $\langle\varphi_v^{(i)}|\varphi_{v'}^{(i)}\rangle = \delta_{vv'}$.
- The modal with quantum number $v = 0$ is the ground state of mode i; $v = 1$ is the first excited state of that mode, etc.
- All VCI configurations are built as products of these *same* ground-state modals.

The VCI wavefunction is expanded as:

$$\boxed{|\Psi_I\rangle = \sum_{\mathbf{n}\in\mathcal{S}} c_{\mathbf{n}}^{(I)}\,|\Phi_{\mathbf{n}}\rangle, \qquad |\Phi_{\mathbf{n}}\rangle = \prod_{i=1}^{M} |\varphi_{n_i}^{(i)}\rangle} \tag{38}$$

Each VSCF modal $|\varphi_{n_i}^{(i)}\rangle$ is itself expanded in the primitive harmonic oscillator basis $\{|\phi_\mu\rangle\}$ with coefficients obtained from the ground-state VSCF calculation:

$$|\varphi_{n_i}^{(i)}\rangle = \sum_{\mu=1}^{N_{\text{exp}}} C_{\mu,n_i}^{(i)}\,|\phi_{\mu-1}\rangle \tag{39}$$

Therefore, a VCI configuration $|\Phi_{\mathbf{n}}\rangle$ can be written explicitly as a linear combination of products of harmonic oscillator functions:

$$|\Phi_{\mathbf{n}}\rangle = \prod_{i=1}^{M}\left(\sum_{\mu_i=1}^{N_{\text{exp}}} C_{\mu_i,n_i}^{(i)}\,|\phi_{\mu_i-1}\rangle\right) = \sum_{\boldsymbol{\mu}}\left(\prod_{i=1}^{M} C_{\mu_i,n_i}^{(i)}\right)\prod_{i=1}^{M}|\phi_{\mu_i-1}\rangle \tag{40}$$

where the sum runs over all $\boldsymbol{\mu} = (\mu_1,\ldots,\mu_M)$ with each $\mu_i = 1,\ldots,N_{\text{exp}}$.

Why use ground-state modals for all configurations? Using a single, fixed set of modals for the entire VCI expansion ensures that the basis functions $\{|\Phi_{\mathbf{n}}\rangle\}$ are mutually orthogonal. This is essential for a standard CI formulation: if different configurations used different state-specific modals, they would not be orthogonal, greatly complicating the Hamiltonian matrix elements and the interpretation of the wavefunction. The ground-state VSCF modals provide a balanced, orthonormal one-mode basis that is equally appropriate for describing ground and excited vibrational states within the CI framework.

5.4 Relation to the Harmonic Oscillator Basis

In the alternative approach where the primitive harmonic oscillator functions themselves are used directly as the one-mode basis (rather than VSCF modals), the CI configurations would simply be:

$$|\Phi_{\mathbf{n}}^{\text{HO}}\rangle = \prod_{i=1}^{M}|\phi_{n_i}\rangle \tag{41}$$

without any summation over expansion coefficients. This is the simplest possible choice: each configuration is a single product of HO functions, and no VSCF precomputation is required.

However, $\langle\Psi|$ V_iBra does not adopt this simpler HO-based CI approach. Instead, it works directly with the VSCF modal basis.

5.5 Precomputed Modal Integral Table

A lookup table stores all needed one-mode integrals:

$$\mathcal{I}_i(v, v'; p) = \langle \varphi_v^{(i)} | Q_i^p | \varphi_{v'}^{(i)} \rangle = \sum_{\mu,\nu} C_{\mu,v}^{(i)} C_{\nu,v'}^{(i)} \langle \phi_{\mu-1} | Q_i^p | \phi_{\nu-1} \rangle \tag{42}$$

Dimensions: $M \times (N_q + 1)^2 \times 6$. Computed once; each lookup is $O(1)$. The table is symmetric: $\mathcal{I}_i(v, v'; p) = \mathcal{I}_i(v', v; p)$.
This avoids the $O(N_{\text{exp}}^2)$ double sum that would otherwise be required for *every* integral evaluation during Hamiltonian construction.

5.6 Vectorised Potentials and Unique-Mode Metadata

The cubic and quartic tensors are stored as one-dimensional arrays indexed by the upper-triangular multi-index. For each term, precomputed metadata stores: the unique mode indices, the power (multiplicity) of each, the number of distinct modes, and a nonzero flag for skipping vanishing terms. This decomposition is performed by the `count_unique_elements` subroutine.

5.7 Sparse Pair List

Two configurations $\mathbf{m}$ and $\mathbf{n}$ can have a nonzero Hamiltonian element only if:

$$n_{\text{diff}}(\mathbf{m}, \mathbf{n}) \equiv |\{i : m_i \neq n_i\}| \leq 4 \tag{43}$$

The code precomputes the complete list of interacting pairs in a two-pass approach: a counting pass to determine the array size, followed by a filling pass. For each pair, the indices of the differing modes are recorded for use by the inverted index.

5.8 Inverted Index for Potential Terms

For off-diagonal pairs ($n_{\text{diff}} \geq 1$), only potential terms involving *all* differing modes can contribute. An inverted index maps each mode to the list of cubic and quartic terms that involve it. When processing a pair where modes $d_1, d_2, \ldots$ differ, the code iterates only over terms from the inverted index of d_1, then verifies that all remaining differing modes also appear in the term. This reduces the number of terms to evaluate dramatically compared to a full scan.

5.9 Prefix–Suffix Overlap Products

The product of one-mode overlaps excluding mode k,

$$S_{\text{all}\setminus k} = \prod_{j \neq k} \mathcal{I}_j(m_j, n_j; 0) \tag{44}$$

is needed for every one-body contribution. Naïvely computing this for each mode costs $O(M)$ per mode and $O(M^2)$ total. ⟨Ψ| V_iBra uses prefix and suffix products to compute all M values in a single $O(M)$ pass:

$$\text{prefix}(0) = 1, \qquad \text{prefix}(k) = \prod_{i=1}^{k} S_i \tag{45}$$

$$\text{suffix}(M+1) = 1, \qquad \text{suffix}(k) = \prod_{i=k}^{M} S_i \tag{46}$$

$$S_{\text{all}\setminus k} = \text{prefix}(k-1) \times \text{suffix}(k+1) \tag{47}$$

where $S_i = \mathcal{I}_i(m_i, n_i; 0)$.

For multi-mode terms (e.g. cubic with unique modes $\{i, j, k\}$), the product excluding all three is computed as $S_{\text{all}}/(S_i \cdot S_j \cdot S_k)$. When any factor vanishes, a fallback computes the product explicitly, skipping the zero modes.

5.10 Hamiltonian Element Structure

The subroutine `compute_H_element` computes a single $H_{\mathbf{mn}}$ as the sum of three contributions:

One-body terms ($n_{\text{diff}} \leq 1$): For each mode i, the diagonal anharmonic corrections are:

$$H^{(1)} = \sum_{i=1}^{M} \left[\mathcal{I}_i(m_i, n_i; 5) + \tilde{\phi}_{iiii}\,\mathcal{I}_i(m_i, n_i; 4) + \tilde{\phi}_{iii}\,\mathcal{I}_i(m_i, n_i; 3) + \tfrac{\omega_i}{2}\,\mathcal{I}_i(m_i, n_i; 2)\right] S_{\text{all}\setminus i} \tag{48}$$

Cubic coupling terms ($n_{\text{diff}} \leq 3$): For each nonzero cubic term with unique modes $\{s_r\}$ and respective powers $\{c_r\}$:

$$H^{(3,\text{term})} = \tilde{\phi}_{\text{term}} \prod_r \mathcal{I}_{s_r}(m_{s_r}, n_{s_r};\, c_r)\; S_{\text{all}\setminus\{s_r\}} \tag{49}$$

Quartic coupling terms ($n_{\text{diff}} \leq 4$): Analogous with up to four unique modes.

For diagonal pairs ($n_{\text{diff}} = 0$), all nonzero terms must be scanned. For off-diagonal pairs, the inverted index restricts the scan to terms involving the first differing mode, then filters for the remaining ones.

5.11 VCI Transition Dipoles (First + Second Order)

Second-order dipole in VCI

The VCI dipole matrix element between configurations $\mathbf{m}$ and $\mathbf{n}$ includes three types of contributions:

(a) Linear dipole, one-body ($n_{\text{diff}} \leq 1$):

$$D_\alpha^{(a)} = \sum_i \frac{\partial \mu_\alpha}{\partial Q_i}\, \mathcal{I}_i(m_i, n_i; 1)\; S_{\text{all}\setminus i} \tag{50}$$

(b) Diagonal second derivative, one-body ($n_{\text{diff}} \leq 1$):

$$D_\alpha^{(b)} = \sum_i \frac{\partial^2 \mu_\alpha}{\partial Q_i^2}\, \mathcal{I}_i(m_i, n_i; 2)\; S_{\text{all}\setminus i} \tag{51}$$

(c) Off-diagonal second derivative, two-body ($n_{\text{diff}} \leq 2$, $i \neq j$):

$$D_\alpha^{(c)} = \sum_{i<j} 2\, \frac{\partial^2 \mu_\alpha}{\partial Q_i \partial Q_j}\, \mathcal{I}_i(m_i, n_i; 1)\, \mathcal{I}_j(m_j, n_j; 1)\; S_{\text{all}\setminus\{i,j\}} \tag{52}$$

The factor of 2 accounts for the symmetric storage of the second-derivative tensor. HO and VSCF include only term (a).

The transformation to eigenstates is performed with BLAS: first `DGEMV` for $\mathbf{t}_\alpha = \boldsymbol{\mu}_\alpha \cdot \mathbf{c}_0$, then `DDOT` for $\langle \Psi_I | \hat{\mu}_\alpha | \Psi_0 \rangle = \mathbf{c}_I^T \cdot \mathbf{t}_\alpha$.

5.12 Diagonalisation

Two LAPACK routines are wrapped:

- `DSYEVD`: divide-and-conquer, computes all eigenvalues. Used when $N_{\text{states}} = |\mathcal{S}|$.
- `DSYEVR`: relatively robust representations, computes only the lowest N_{states} eigenvalues. Significantly faster for partial spectra.

6 Selected Vibrational CI (SCI)

6.1 Motivation

Full VCI becomes prohibitive for large configuration spaces. Most configurations contribute negligibly to the low-lying states of interest. SCI, inspired by CIPSI and heat-bath CI from electronic structure theory, starts from a compact reference, uses perturbation theory to screen external configurations, and adds only the most important ones.

6.2 CISD Reference

The default reference space comprises all configurations with $\sum_i n_i \leq 2$ (ground state, singles, doubles). Its dimension is $1 + 2M + \binom{M}{2}$, meaning it is linear in M for single excitations and quadratic for doubles. The Hamiltonian in the CISD space is built and diagonalised exactly, yielding reference energies $E_I^{(0)}$ and eigenvectors $|\Psi_I^{(0)}\rangle = \sum_{\mathbf{n}\in\mathrm{CISD}} c_{\mathbf{n}}^{(I)} |\Phi_{\mathbf{n}}\rangle$. Therefore, further discussion in this session will be referent to this reference space. Different reference spaces (CIS, CISDT, CISDTQ) or user-provided state lists can be specified via the `MAXSCI` keyword.

6.3 Epstein–Nesbet PT2 Screening

For each external configuration $|\Phi_\alpha\rangle$ and CISD eigenstate I:

$$e_\alpha^{(I)} = \frac{\left|\langle\Phi_\alpha|\hat{H}|\Psi_I^{\mathrm{ref}}\rangle\right|^2}{E_I^{\mathrm{ref}} - H_{\alpha\alpha}} \tag{53}$$

where the coupling is $\langle\Phi_\alpha|\hat{H}|\Psi_I^{\mathrm{ref}}\rangle = \sum_{\mathbf{n}\in\mathrm{ref}} c_{\mathbf{n}}^{(I)} H_{\alpha\mathbf{n}}$.

The EN-PT2 zeroth-order energy is the diagonal Hamiltonian element $H_{\alpha\alpha}$, which is more natural for the vibrational problem than orbital-energy-based denominators. Configurations with large $|e_\alpha^{(I)}|$ are the most important.

6.4 Top-N Selection and Union

For each CISD eigenstate, the N_{sel} external configurations with the largest $|e_\alpha^{(I)}|$ are selected via a maintained sorted list with insertion. The final active space is the union across all states:

$$\mathcal{S}_{\mathrm{active}} = \mathcal{S}_{\mathrm{CISD}} \cup \bigcup_I \mathcal{S}_{\mathrm{sel}}^{(I)} \tag{54}$$

The full Hamiltonian is then built and diagonalised for this active space using the same `compute_H_element` kernel and all the same optimisations as full VCI. The `MAXSCI` variable is the N_{sel} maximum per VCISD state. Note that one external state can contribute to many VCISD states, so the final number of states can vary.

Activating Selected CI. SCI is activated automatically when `MAXSCI` $>$ 0 is set in `input_vscf.txt` and no molecular symmetry is specified (i.e. `PGROUP` is absent or set to `C1`). If symmetry is active (any group other than `C1`), the code defaults to the symmetry-adapted full VCI regardless of `MAXSCI`.

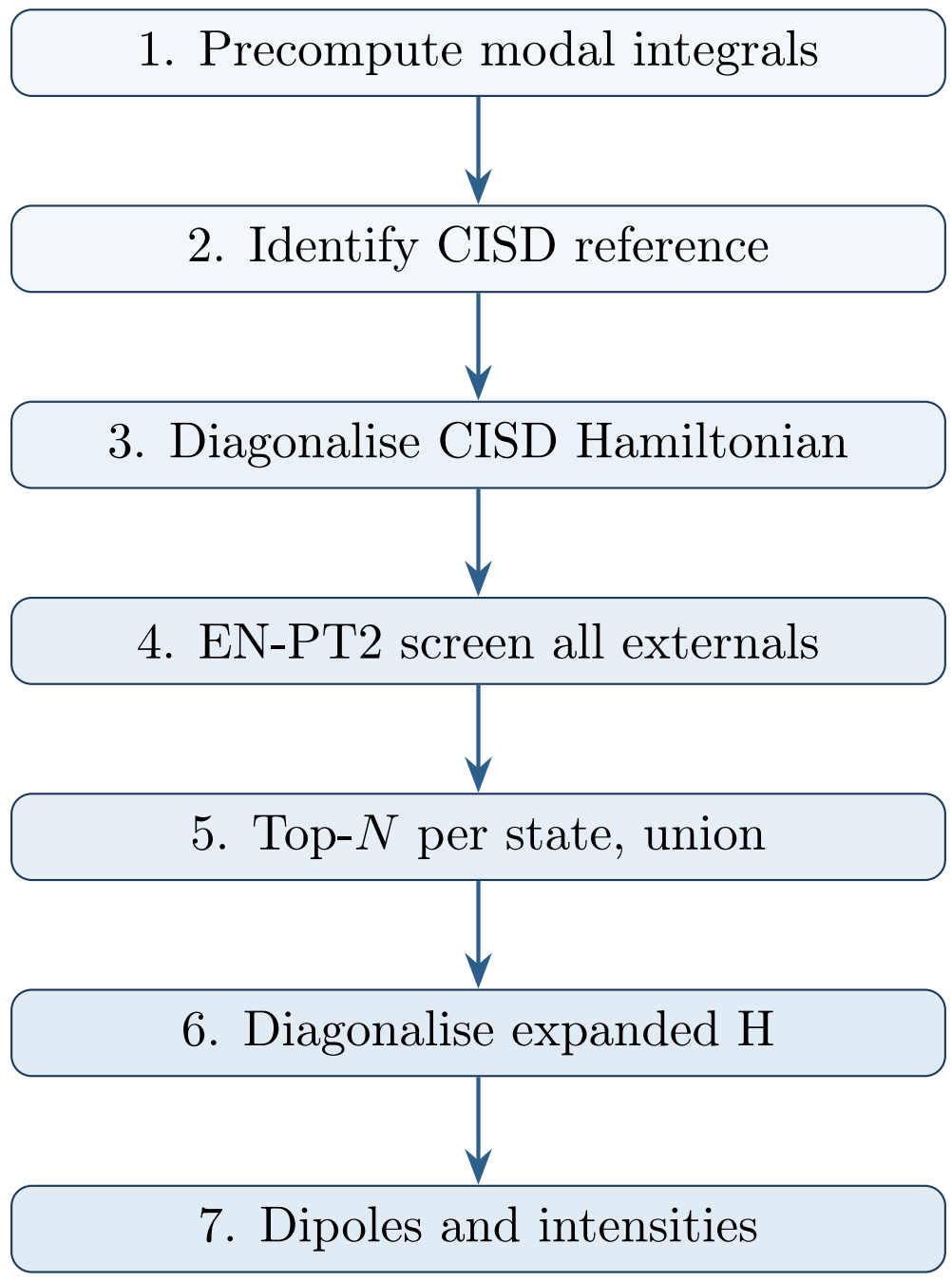

Figure S1: SCI workflow. The loop from step 4 to step 8 repeats for at most `MAXSCI` iterations.

7 Symmetry-Adapted Vibrational CI

7.1 Motivation

The vibrational Hamiltonian $\hat{H}_{\text{vib}}$ is invariant under every symmetry operation of the molecular point group $\mathcal{G}$: it commutes with all group elements, $[\hat{H}_{\text{vib}}, \hat{R}] = 0$ for all $\hat{R} \in \mathcal{G}$. As a direct consequence, its matrix representation in any symmetry-adapted basis is *block-diagonal*: matrix elements connecting basis functions that transform according to *different* irreducible representations (irreps) are identically zero. Exploiting this structure in the VCI calculation yields three direct benefits:

- **Reduced diagonalisation cost.** If the configuration space $\mathcal{S}$ decomposes into symmetry blocks of sizes $\{d_\Gamma\}$, the diagonalisation cost is reduced from $\mathcal{O}(|\mathcal{S}|^3)$ to $\sum_\Gamma \mathcal{O}(d_\Gamma^3)$. For a molecule with $n_\Gamma = 4$ equally populated irreps this represents a factor of $n_\Gamma^2 = 16$ reduction.
- **Exact selection rules.** Matrix elements between configurations of different symmetry are identically zero by group theory, not merely small. They need never be computed.
- **Symmetry labels on eigenstates.** Every VCI eigenstate $|\Psi_I\rangle$ carries an unambiguous irrep label Γ_I, enabling application of infrared selection rules and direct comparison with experimental band assignments.

Restriction to Abelian groups with exclusively 1D irreps

The symmetry-adapted VCI is implemented only for point groups in which *every* irreducible representation is one-dimensional. For a finite group this is equivalent to the group being Abelian (all elements commute). The supported groups are:

$$C_1, \quad C_s, \quad C_i, \quad C_2, \quad C_{2h}, \quad C_{2v}, \quad D_2, \quad D_{2h}. \tag{55}$$

Groups containing two-dimensional (E) or higher irreps (e.g. C_{3v}, T_d, O_h) are **not directly supported**. The code contains no implementation of C_3, S_4, or any other symmetry elements beyond E, C_2, σ, and i, and it cannot construct the matrices or character tables of non-Abelian groups. If the true molecular point group is not in the supported set, the user may attempt to use the largest Abelian subgroup that is supported (for example, C_s for a C_{3v} molecule), but should verify the results carefully against a C_1 reference calculation (see Section 7.8). Alternatively, setting C_1 always produces correct (unsymmetrised) results.

7.2 Representation Theory Background

7.2.1 Irreducible Representations and Character Tables

A *representation* Γ of a group $\mathcal{G}$ assigns to each element $\hat{R} \in \mathcal{G}$ a matrix $D^\Gamma(\hat{R})$ preserving group multiplication: $D^\Gamma(\hat{R}_1\hat{R}_2) = D^\Gamma(\hat{R}_1)D^\Gamma(\hat{R}_2)$. An irreducible representation (irrep) cannot be further block-diagonalised. For the eight supported Abelian groups all irreps are one-dimensional: $D^\Gamma(\hat{R}) = \chi(\Gamma, \hat{R}) \in \{\pm 1\}$. The *character table* collects these values as an $n_\Gamma \times |\mathcal{G}|$ matrix $\chi(\Gamma, \hat{R})$.

7.2.2 Great Orthogonality Theorem and Reduction Formula

The irreps satisfy:

$$\frac{1}{|\mathcal{G}|} \sum_{\hat{R} \in \mathcal{G}} \chi(\Gamma_i, \hat{R})\, \chi(\Gamma_j, \hat{R}) = \delta_{ij} \tag{56}$$

A reducible representation with characters $\chi(\hat{R})$ contains irrep Γ_i with multiplicity:

$$\boxed{n_i = \frac{1}{|\mathcal{G}|} \sum_{\hat{R}\in\mathcal{G}} \chi(\hat{R})\, \chi(\Gamma_i, \hat{R})} \tag{57}$$

This is the key tool for decomposing reducible representations into irreps and is applied both to the $3N_{\text{at}}$-dimensional Cartesian representation and to individual normal modes.

7.2.3 Direct Products

The direct product of two 1D irreps is computed element-wise:

$$\chi(\Gamma_i \otimes \Gamma_j, \hat{R}) = \chi(\Gamma_i, \hat{R}) \cdot \chi(\Gamma_j, \hat{R}), \qquad \forall\, \hat{R} \in \mathcal{G} \tag{58}$$

Since all characters are ± 1, the result is again ± 1 and always coincides with exactly one irrep: $\Gamma_i \otimes \Gamma_j = \Gamma_k$. The $n_\Gamma \times n_\Gamma$ integer table $k = f(i, j)$ is precomputed once and used throughout the symmetry-adapted VCI.

Table S2 gives the complete character tables for all eight supported groups.

Table S2: Character tables for the eight supported Abelian groups. Irrep index 1 is the totally symmetric species in every group. All character values are $+1$ or -1.

C_1	E
A	1

C_s	E	σ_h
A'	1	1
A''	1	−1

C_i	E	i
A_g	1	1
A_u	1	−1

C_2	E	C_2
A	1	1
B	1	−1

C_{2h}	E	C_2	i	σ_h
A_g	1	1	1	1
B_g	1	−1	1	−1
A_u	1	1	−1	−1
B_u	1	−1	−1	1

C_{2v}	E	C_2	σ_v	σ'_v
A_1	1	1	1	1
A_2	1	1	−1	−1
B_1	1	−1	1	−1
B_2	1	−1	−1	1

D_2	E	$C_2(z)$	$C_2(y)$	$C_2(x)$
A	1	1	1	1
B_1	1	1	−1	−1
B_2	1	−1	1	−1
B_3	1	−1	−1	1

D_{2h}	E	$C_2(z)$	$C_2(y)$	$C_2(x)$	i	σ_h	σ_v	σ'_v
A_g	1	1	1	1	1	1	1	1
B_{1g}	1	1	−1	−1	1	1	−1	−1
B_{2g}	1	−1	1	−1	1	−1	1	−1
B_{3g}	1	−1	−1	1	1	−1	−1	1
A_u	1	1	1	1	−1	−1	−1	−1
B_{1u}	1	1	−1	−1	−1	−1	1	1
B_{2u}	1	−1	1	−1	−1	1	−1	1
B_{3u}	1	−1	−1	1	−1	1	1	−1

As an example, Table S3 shows the direct-product table for C_{2v}.

Table S3: Direct-product table for C_{2v}.

$\otimes$	A_1	A_2	B_1	B_2
A_1	A_1	A_2	B_1	B_2
A_2	A_2	A_1	B_2	B_1
B_1	B_1	B_2	A_1	A_2
B_2	B_2	B_1	A_2	A_1

Principal axis orientation and character table interpretation

The character tables in Section S2 are written with conventional axis conventions (e.g., for C_{2v}, the C_2 axis is usually taken as the z-axis, and the two mirror planes as $\sigma_v(xz)$ and $\sigma_v'(yz)$). However, the program automatically determines the principal axis frame (PAF) by diagonalising the inertia tensor, and the orientation of the molecule in this frame depends on the numerical eigenvectors returned by LAPACK's `dsyev`. This means that the principal axis identified as a, b, or c (with $I_a \leq I_b \leq I_c$) may not align with the conventional z-axis assumed in the character table.

Consequently, the irrep labels assigned to normal modes and to VCI configurations are correct *relative to the program's internal orientation*, but the user must interpret them with care. For example, a mode labelled B_1 in a C_{2v} calculation by the program may correspond to the conventional B_2 species if the program's axis assignment differs from the standard convention. The diagnostic output printed to `stdout` and `debug_sym.txt` during the symmetry initialisation phase reports the chosen orientation for each symmetry element (e.g., "C2=ax 3 sv normals: ax 1 ax 2"), which can be used to map the program's labels to conventional species.

The user should:

- Inspect the orientation detection output to understand which principal axis direction corresponds to each symmetry element.
- Compare the program's irrep assignments to expectations based on the known vibrational modes of the molecule.
- When in doubt, run a C_1 calculation to verify that the symmetry-adapted eigenvalues match the unsymmetrised results, regardless of the labelling convention.

The irrep labels themselves are consistent within the calculation and correctly block-diagonalise the Hamiltonian, even if they do not follow the most common textbook convention for axis labelling.

7.3 Assignment of Normal Modes to Irreducible Representations

7.3.1 The $3N_{\mathrm{at}}$-Dimensional Representation of Atomic Displacements

A molecular geometry with N_{at} atoms defines a $3N_{\mathrm{at}}$-dimensional vector space of Cartesian displacements. Every symmetry operation $\hat{R} \in \mathcal{G}$ acts on this space as a $3N_{\mathrm{at}} \times 3N_{\mathrm{at}}$ orthogonal matrix $\mathbf{D}(\hat{R})$ combining two simultaneous actions:

1. A **permutation** of atoms: atom a is mapped to atom $\hat{R}(a)$.
2. A **rotation** (or rotation-reflection) of each displacement vector by the 3×3 matrix $\mathbf{R}$ associated with the operation.

The set $\{\mathbf{D}(\hat{R})\}$ forms a $3N_{\mathrm{at}}$-dimensional *reducible* representation Γ_{3N} of $\mathcal{G}$.

7.3.2 Structure of the $3N_{\text{at}} \times 3N_{\text{at}}$ Representation Matrix

The full representation matrix $\mathbf{D}(\hat{R})$ acts on the $3N_{\text{at}}$-dimensional displacement vector

$$\mathbf{q} = \left(x_1, y_1, z_1,\ x_2, y_2, z_2,\ \ldots,\ x_{N_{\text{at}}}, y_{N_{\text{at}}}, z_{N_{\text{at}}}\right)^T \tag{59}$$

and is built from $N_{\text{at}} \times N_{\text{at}}$ blocks of size 3×3. The block in row b, column a is:

$$\left[\mathbf{D}(\hat{R})\right]_{ba} = \begin{cases} \mathbf{R} & \text{if } \hat{R}(a) = b \\ \mathbf{0}_{3\times 3} & \text{otherwise} \end{cases} \tag{60}$$

where $\mathbf{R}$ is the 3×3 matrix of the point group operation (rotation, reflection, or inversion) and $\hat{R}(a)$ denotes the atom to which atom a is mapped under the operation. Each row and each column of the block matrix contains exactly one nonzero 3×3 block.

Example: $\sigma_v(xz)$ for water (C_{2v}). Label the atoms as $a = 1$ (O), $a = 2$ (H_L), $a = 3$ (H_R). The reflection $\sigma_v(xz)$, with normal along y, has 3×3 matrix

$$\mathbf{R}_{\sigma_v} = \begin{pmatrix} 1 & 0 & 0 \\ 0 & -1 & 0 \\ 0 & 0 & 1 \end{pmatrix} \tag{61}$$

and permutes atoms as $\hat{R}(1) = 1$, $\hat{R}(2) = 3$, $\hat{R}(3) = 2$. The 9×9 representation matrix is:

$$\mathbf{D}(\sigma_v) = \begin{pmatrix} \mathbf{R}_{\sigma_v} & \mathbf{0} & \mathbf{0} \\ \mathbf{0} & \mathbf{0} & \mathbf{R}_{\sigma_v} \\ \mathbf{0} & \mathbf{R}_{\sigma_v} & \mathbf{0} \end{pmatrix} = \left(\begin{array}{ccc|ccc|ccc} 1 & 0 & 0 & 0 & 0 & 0 & 0 & 0 & 0 \\ 0 & -1 & 0 & 0 & 0 & 0 & 0 & 0 & 0 \\ 0 & 0 & 1 & 0 & 0 & 0 & 0 & 0 & 0 \\ \hline 0 & 0 & 0 & 0 & 0 & 0 & 1 & 0 & 0 \\ 0 & 0 & 0 & 0 & 0 & 0 & 0 & -1 & 0 \\ 0 & 0 & 0 & 0 & 0 & 0 & 0 & 0 & 1 \\ \hline 0 & 0 & 0 & 1 & 0 & 0 & 0 & 0 & 0 \\ 0 & 0 & 0 & 0 & -1 & 0 & 0 & 0 & 0 \\ 0 & 0 & 0 & 0 & 0 & 1 & 0 & 0 & 0 \end{array}\right) \tag{62}$$

The block structure is visible: the $(1, 1)$ diagonal block is $\mathbf{R}_{\sigma_v}$ (O maps to itself), the $(2, 3)$ and $(3, 2)$ off-diagonal blocks are $\mathbf{R}_{\sigma_v}$ ($\text{H}_\text{L} \leftrightarrow \text{H}_\text{R}$), and all other blocks are zero.

Example: $C_2(z)$ for water. The C_2 rotation about z has $\mathbf{R}_{C_2} = \text{diag}(-1, -1, +1)$ and the same atom permutation: $\hat{R}(1) = 1$, $\hat{R}(2) = 3$, $\hat{R}(3) = 2$.

$$\mathbf{D}(C_2) = \left(\begin{array}{ccc|ccc|ccc} -1 & 0 & 0 & 0 & 0 & 0 & 0 & 0 & 0 \\ 0 & -1 & 0 & 0 & 0 & 0 & 0 & 0 & 0 \\ 0 & 0 & 1 & 0 & 0 & 0 & 0 & 0 & 0 \\ \hline 0 & 0 & 0 & 0 & 0 & 0 & -1 & 0 & 0 \\ 0 & 0 & 0 & 0 & 0 & 0 & 0 & -1 & 0 \\ 0 & 0 & 0 & 0 & 0 & 0 & 0 & 0 & 1 \\ \hline 0 & 0 & 0 & -1 & 0 & 0 & 0 & 0 & 0 \\ 0 & 0 & 0 & 0 & -1 & 0 & 0 & 0 & 0 \\ 0 & 0 & 0 & 0 & 0 & 1 & 0 & 0 & 0 \end{array}\right) \tag{63}$$

The permutation pattern (which blocks are nonzero) is identical to σ_v; only the 3×3 rotation matrix differs.

7.3.3 Character of the $3N_{\text{at}}$-Dimensional Representation

The character (trace) of $\mathbf{D}(\hat{R})$ receives contributions only from the diagonal 3×3 blocks, which exist only when $\hat{R}(a) = a$:

$$\chi^{3N}(\hat{R}) = \mathrm{Tr}\big[\mathbf{D}(\hat{R})\big] = \sum_{\substack{a=1\\ \hat{R}(a)=a}}^{N_{\text{at}}} \mathrm{Tr}(\mathbf{R}) = N_{\text{unmoved}}(\hat{R}) \cdot \mathrm{Tr}(\mathbf{R}) \tag{64}$$

Operation	$\mathrm{Tr}(\mathbf{R})$	N_{unmoved} (typical)
E	$+3$	all atoms
C_2	-1	atoms on the axis
σ	$+1$	atoms in the plane
i	-3	atom at origin only

For water: $\chi^{3N}(E) = 3\times3 = 9$, $\chi^{3N}(C_2) = 1\times(-1) = -1$ (only O unmoved), $\chi^{3N}(\sigma_v) = 3\times1 = 3$, $\chi^{3N}(\sigma_v') = 1\times1 = 1$.

Decomposing Γ_{3N} via Eq. (57) and subtracting translations and rotations:

$$\Gamma_{\text{vib}} = \Gamma_{3N} - \Gamma_{\text{trans}} - \Gamma_{\text{rot}} \tag{65}$$

gives the symmetry species and multiplicities of the $3N_{\text{at}} - 6$ vibrational modes of a nonlinear molecule.

7.3.4 Character of a Single Normal Mode: the Full Permutation-Rotation Formula

The reduction formula gives the total count of each symmetry species but does not assign a species to each individual mode. For mode k with displacement vector $\mathbf{L}^{(k)} = (L^{(k)}_{x,1}, L^{(k)}_{y,1}, L^{(k)}_{z,1}, \ldots, L^{(k)}_{z,N_{\text{at}}})^T$ (a $3N_{\text{at}}$-dimensional column vector), the character of the one-dimensional representation spanned by this mode is:

$$\chi^{(k)}(\hat{R}) = \frac{(\mathbf{L}^{(k)})^T\,\mathbf{D}(\hat{R})\,\mathbf{L}^{(k)}}{\|\mathbf{L}^{(k)}\|^2} \tag{66}$$

Expanding the matrix–vector product. The action of $\mathbf{D}(\hat{R})$ on $\mathbf{L}^{(k)}$ follows from the block structure Eq. (60). The 3×1 sub-vector of $\mathbf{D}(\hat{R})\,\mathbf{L}^{(k)}$ at atom position b is:

$$\big[\mathbf{D}(\hat{R})\,\mathbf{L}^{(k)}\big]_b = \sum_{a=1}^{N_{\text{at}}} [\mathbf{D}(\hat{R})]_{ba}\,\mathbf{l}^{(k)}_a = \mathbf{R}\,\mathbf{l}^{(k)}_{\hat{R}^{-1}(b)} \tag{67}$$

where $\mathbf{l}^{(k)}_a = (L^{(k)}_{x,a}, L^{(k)}_{y,a}, L^{(k)}_{z,a})^T$ is the three-component displacement of atom a in mode k, and $\hat{R}^{-1}(b)$ is the unique atom that maps *to* position b under the operation $\hat{R}$. Substituting into the quadratic form:

$$\begin{aligned}(\mathbf{L}^{(k)})^T\,\mathbf{D}(\hat{R})\,\mathbf{L}^{(k)} &= \sum_{b=1}^{N_{\text{at}}} (\mathbf{l}^{(k)}_b)^T\,\mathbf{R}\,\mathbf{l}^{(k)}_{\hat{R}^{-1}(b)} \\ &= \sum_{a=1}^{N_{\text{at}}} (\mathbf{l}^{(k)}_{\hat{R}(a)})^T\,\mathbf{R}\,\mathbf{l}^{(k)}_a \end{aligned} \tag{68}$$

where the second line substitutes $a = \hat{R}^{-1}(b)$. The character is therefore:

$$\boxed{\chi^{(k)}(\hat{R}) = \frac{1}{\|\mathbf{L}^{(k)}\|^2} \sum_{a=1}^{N_{\text{at}}} \sum_{\alpha,\beta} L^{(k)}_{\alpha,\hat{R}(a)}\,R_{\alpha\beta}\,L^{(k)}_{\beta,a}} \tag{69}$$

where the sum runs over **all** atoms and $\|\mathbf{L}^{(k)}\|^2 = \sum_{a=1}^{N_\text{at}} \sum_\alpha (L^{(k)}_{\alpha,a})^2$ is the full squared norm.

All atoms contribute to the character

Equation (69) sums over *all* N_at atoms, not only those that map to themselves. The atom permutation table $\hat{R}(a)$ determines which displacement vectors are paired in each term: atom a's displacement is rotated by $\mathbf{R}$ and then dotted with the displacement of atom $\hat{R}(a)$. Only when an atom maps to itself ($\hat{R}(a) = a$) does the term reduce to $(\mathbf{l}_a^{(k)})^T \mathbf{R}\, \mathbf{l}_a^{(k)}$, the "diagonal" contribution. Off-diagonal terms (atoms permuted to different atoms) can contribute positively or negatively, and are essential for obtaining the correct character ± 1 when the mode displacement is concentrated on atoms that are permuted by the operation.

7.3.5 Verification: Why the Formula Yields ±1

For a mode belonging to irrep Γ_k, the equivariance condition requires:

$$\sum_\beta R_{\alpha\beta}\, L^{(k)}_{\beta,a} = \chi(\Gamma_k, \hat{R})\, L^{(k)}_{\alpha,\hat{R}(a)}, \qquad \forall\, a,\, \alpha \tag{70}$$

Substituting into the quadratic form Eq. (68):

$$\begin{aligned}\sum_{a=1}^{N_\text{at}} (\mathbf{l}^{(k)}_{\hat{R}(a)})^T\, \mathbf{R}\, \mathbf{l}^{(k)}_a &= \sum_a \sum_\alpha L^{(k)}_{\alpha,\hat{R}(a)} \underbrace{\sum_\beta R_{\alpha\beta}\, L^{(k)}_{\beta,a}}_{=\chi(\Gamma_k,\hat{R})\, L^{(k)}_{\alpha,\hat{R}(a)}} \\ &= \chi(\Gamma_k, \hat{R}) \sum_a \sum_\alpha \left(L^{(k)}_{\alpha,\hat{R}(a)}\right)^2 = \chi(\Gamma_k, \hat{R})\, \|\mathbf{L}^{(k)}\|^2\end{aligned} \tag{71}$$

confirming $\chi^{(k)}(\hat{R}) = \chi(\Gamma_k, \hat{R}) \in \{+1, -1\}$. The final step uses the fact that $\hat{R}$ is a permutation, so $\sum_a f(\hat{R}(a)) = \sum_a f(a)$.

7.3.6 Simplification for Unmoved Atoms

For atoms with $\hat{R}(a) = a$, the term in the sum Eq. (69) is $(\mathbf{l}_a^{(k)})^T \mathbf{R}\, \mathbf{l}_a^{(k)}$, which is purely local to atom a. For atoms with $\hat{R}(a) \neq a$, the term involves the cross-pairing $(\mathbf{l}^{(k)}_{\hat{R}(a)})^T \mathbf{R}\, \mathbf{l}_a^{(k)}$. In many textbook treatments, only the diagonal contributions from unmoved atoms are retained:

$$\chi^{(k)}_\text{diag}(\hat{R}) = \frac{1}{\displaystyle\sum_{\substack{a=1\\ \hat{R}(a)=a}}^{N_\text{at}} |\mathbf{l}_a^{(k)}|^2} \sum_{\substack{a=1\\ \hat{R}(a)=a}}^{N_\text{at}} (\mathbf{l}_a^{(k)})^T\, \mathbf{R}\, \mathbf{l}_a^{(k)} \tag{72}$$

This "diagonal-only" formula is correct when the unmoved atoms carry all (or the dominant fraction) of the displacement amplitude. However, it **fails** when the mode displacement is concentrated on atoms that are permuted by the operation, leaving the unmoved atoms with negligible displacement. In such cases the numerator and denominator in Eq. (72) are both near zero, and their ratio takes an arbitrary value unrelated to the true character.

Failure of the diagonal-only formula

Consider a C_{2v} molecule where the σ_v reflection maps $H_1 \leftrightarrow H_2$ and leaves O unmoved. For an asymmetric stretch mode where the displacement is almost entirely on the two hydrogen

atoms, the oxygen displacement $\mathbf{l}_O$ is numerically negligible ($|\mathbf{l}_O|^2 \sim 10^{-12}$). The diagonal-only formula computes $\chi \approx 0/0 \approx 0.88$ (or any other arbitrary value), while the full formula Eq. (69) correctly yields $\chi = -1$ because the cross-terms from the $H_1 \leftrightarrow H_2$ permutation contribute the dominant signal.

7.3.7 Reference Frame Requirement

Equation (69) requires the geometry, displacement vectors, and symmetry operation matrices to be expressed in the *same* frame. The symmetry operations in Table S2 are defined in the *principal axis frame* (PAF):

- Origin at the centre of mass.
- Axes a, b, c along the principal axes of inertia (ordered by increasing moment).

Both the geometry and displacement vectors from `normal_mode.txt` are rotated into the PAF via:

Step 1: Centre of mass. $\mathbf{r}_a^{(0)} = \mathbf{r}_a - \sum_b m_b \mathbf{r}_b / \sum_b m_b$

Step 2: Inertia tensor. $I_{pq} = \sum_a m_a(\delta_{pq}|\mathbf{r}_a^{(0)}|^2 - r_{a,p}^{(0)} r_{a,q}^{(0)})$, diagonalised by `dsyev` (LAPACK) to give rotation matrix $\mathbf{P}$ whose columns are the principal axes.

Step 3: Rotation. $r_{\alpha,a}^{\mathrm{PAF}} = \sum_\beta P_{\beta\alpha}\, r_{\beta,a}^{(0)}$, $\quad L_{\alpha,a}^{(k),\mathrm{PAF}} = \sum_\beta P_{\beta\alpha}\, L_{\beta,a}^{(k)}$.

7.3.8 Atom Permutation Table and the Projection Cutoff

The full character formula Eq. (69) requires knowledge of the atom permutation $\hat{R}(a)$ for every operation $\hat{R}$ and every atom a. This is determined numerically: the PAF position of atom a is transformed by $\mathbf{R}$, and the image atom $\hat{R}(a) = b$ is found as the atom of the same element whose PAF position is closest to $\mathbf{R}\,\mathbf{r}_a^{\mathrm{PAF}}$. The match is accepted if the distance falls below the user-specified threshold δ_{proj}:

$$\left\|\mathbf{R}\,\mathbf{r}_a^{\mathrm{PAF}} - \mathbf{r}_b^{\mathrm{PAF}}\right\| < \delta_{\mathrm{proj}}, \qquad b = \hat{R}(a) \tag{73}$$

The value of δ_{proj} is set by the `PROJCT` keyword in `input_vscf.txt` and defaults to 0.05 Å when the keyword is absent.

Physical meaning and recommended values of `PROJCT`

The cutoff δ_{proj} quantifies how far an atom's image may deviate from an actual atomic position before the permutation mapping is declared invalid. For an ideal molecular geometry fully consistent with the assigned point group, every atom has an exact image and the distance is machine zero.
In practice, geometries from electronic structure calculations contain small numerical noise (sub-mÅ deviations from exact symmetry), and the coordinates are stored with finite precision. A cutoff in the range 0.02–0.10 Å is appropriate:

- Values **too small** ($\delta \lesssim 0.01$ Å): atoms that have valid images may fail the distance test due to rounding in the coordinate file, producing missing permutation entries and incorrect characters.
- Values **too large** ($\delta \gtrsim 0.5$ Å): atoms that genuinely have no image under the operation (indicating an incorrect group assignment) may be incorrectly matched, masking the error.

The default 0.05 Å is a conservative choice that works reliably for rigid organic and inorganic

molecules. For molecules with very long bonds (metal complexes, weakly bound clusters) a slightly larger value may be necessary; for highly symmetric near-ideal geometries the default is always safe.

7.3.9 Irrep Identification

The irrep of mode k is identified by minimum L^1 distance between the computed character vector and each row of the character table:

$$\Gamma_k = \arg\min_{\Gamma} \sum_{\hat{R}\in\mathcal{G}} \left|\chi^{(k)}(\hat{R}) - \chi(\Gamma, \hat{R})\right| \tag{74}$$

For a properly computed normal mode the minimum is ≈ 0 and all other rows have distance ≥ 2, giving an unambiguous assignment.

7.3.10 Complete Example: Water in C_{2v}

Water (H_2O), $N_{\text{at}} = 3$, $M = 3$ vibrational modes. PAF: O at origin, C_2 along z, molecule in xz-plane.

Step 1: Γ_{3N} characters.

	E	C_2	$\sigma_v(xz)$	$\sigma'_v(yz)$
N_{unmoved}	3	1	3	1
$\text{Tr}(\mathbf{R})$	+3	−1	+1	+1
χ^{3N}	9	−1	3	1

Step 2: Reduction.

$$n_{A_1} = \tfrac{1}{4}(9 - 1 + 3 + 1) = 3 \tag{75}$$
$$n_{A_2} = \tfrac{1}{4}(9 - 1 - 3 - 1) = 1 \tag{76}$$
$$n_{B_1} = \tfrac{1}{4}(9 + 1 + 3 - 1) = 3 \tag{77}$$
$$n_{B_2} = \tfrac{1}{4}(9 + 1 - 3 + 1) = 2 \tag{78}$$

$\Gamma_{3N} = 3A_1 + A_2 + 3B_1 + 2B_2$.

Step 3: Remove trans/rot. $T_z \in A_1$, $T_x \in B_1$, $T_y \in B_2$, $R_z \in A_2$, $R_x \in B_1$, $R_y \in B_2$.

$$\Gamma_{\text{vib}} = 2A_1 + B_1 \tag{79}$$

Step 4: Individual mode characters using the full formula. Consider the asymmetric stretch ν_3 under $\sigma_v(xz)$. O is unmoved: $\hat{R}(1) = 1$. The two hydrogens are swapped: $\hat{R}(2) = 3$, $\hat{R}(3) = 2$.

The displacement vectors (schematic, in the xz-plane) are: $\mathbf{l}_\text{O} \approx \mathbf{0}$, $\mathbf{l}_{\text{H}_\text{L}} = (d_x, 0, d_z)$, $\mathbf{l}_{\text{H}_\text{R}} = (-d_x, 0, -d_z)$ (anti-phase motion).

The full formula Eq. (69) gives three terms:

$$\text{O (self):} \quad (\mathbf{l}_\text{O})^T\, \mathbf{R}_{\sigma_v}\, \mathbf{l}_\text{O} \approx 0 \tag{80}$$
$$\text{H}_\text{L} \to \text{H}_\text{R}: \quad (\mathbf{l}_{\text{H}_\text{R}})^T\, \mathbf{R}_{\sigma_v}\, \mathbf{l}_{\text{H}_\text{L}} = (-d_x, 0, -d_z)^T(d_x, 0, d_z) = -d_x^2 - d_z^2 \tag{81}$$
$$\text{H}_\text{R} \to \text{H}_\text{L}: \quad (\mathbf{l}_{\text{H}_\text{L}})^T\, \mathbf{R}_{\sigma_v}\, \mathbf{l}_{\text{H}_\text{R}} = (d_x, 0, d_z)^T(-d_x, 0, -d_z) = -d_x^2 - d_z^2 \tag{82}$$

Numerator $= 0 + (-d_x^2 - d_z^2) + (-d_x^2 - d_z^2) = -2(d_x^2 + d_z^2)$. Denominator $\approx 2(d_x^2 + d_z^2)$. Therefore $\chi^{(\nu_3)}(\sigma_v) = -1$, as expected for B_1.

Note that the diagonal-only formula would have computed $\chi \approx 0/0$ (since the oxygen contribution is negligible), giving an incorrect result.

The complete character table for all three modes:

Mode	E	C_2	σ_v	σ'_v	Γ_k
ν_1 (sym. stretch)	+1	+1	+1	+1	A_1
ν_2 (bend)	+1	+1	+1	+1	A_1
ν_3 (asym. stretch)	+1	−1	−1	+1	B_2

Each vector matches exactly one row, confirming $\Gamma_{\text{vib}} = 2A_1 + B_2$.

7.4 Symmetry of VCI Configurations

7.4.1 Parity of One-Mode States

The harmonic oscillator eigenfunctions satisfy $\phi_n(-Q) = (-1)^n \phi_n(Q)$. The VSCF modals retain this parity structure because the diagonal effective potential is even in Q_i at a stationary point. A symmetry operation $\hat{R}$ acts on Q_i as multiplication by $\chi(\Gamma_i, \hat{R}) = \pm 1$:

$$\hat{R}\,\varphi_{n_i}^{(i)}(Q_i) = \left[\chi(\Gamma_i, \hat{R})\right]^{n_i} \varphi_{n_i}^{(i)}(Q_i) \tag{83}$$

Therefore:

$$\Gamma\left(\varphi_{n_i}^{(i)}\right) = \begin{cases} \Gamma_1 & n_i \text{ even} \\ \Gamma_i & n_i \text{ odd} \end{cases} \tag{84}$$

7.4.2 Symmetry of a Multi-Mode Configuration

The irrep of a Hartree product is the direct product of the contributing one-mode irreps:

$$\Gamma(\Phi_{\mathbf{n}}) = \bigotimes_{i=1}^{M} \Gamma\left(\varphi_{n_i}^{(i)}\right) = \bigotimes_{\substack{i=1 \\ n_i \text{ odd}}}^{M} \Gamma_i \tag{85}$$

where modes with even quanta contribute Γ_1 and are omitted. The product is evaluated sequentially using the precomputed integer table.

Example (C_{2v}, water): $\Gamma_{\nu_1} = A_1$, $\Gamma_{\nu_2} = A_1$, $\Gamma_{\nu_3} = B_2$.

$\mathbf{n}$	Odd-quanta modes	Direct product	$\Gamma(\Phi_{\mathbf{n}})$
$(0, 0, 0)$	—	A_1	A_1
$(1, 0, 0)$	ν_1	A_1	A_1
$(0, 0, 1)$	ν_3	B_2	B_2
$(1, 0, 1)$	ν_1, ν_3	$A_1 \otimes B_2$	B_2
$(0, 0, 2)$	—	A_1	A_1
$(1, 1, 1)$	all	$A_1 \otimes A_1 \otimes B_2$	B_2

7.5 Block-Diagonal Structure of the VCI Hamiltonian

7.5.1 Vanishing of Cross-Symmetry Matrix Elements

For $\Gamma_{\mathbf{m}} \neq \Gamma_{\mathbf{n}}$:

$$\boxed{H_{\mathbf{mn}} = 0} \tag{86}$$

because $\hat{H}_{\text{vib}}$ transforms as Γ_1 and the integrand transforms as $\Gamma_{\mathbf{m}} \otimes \Gamma_{\mathbf{n}} \neq \Gamma_1$. This is exact and holds for every term in the QFF.

7.5.2 Block Structure and Diagonalisation

The configuration space partitions into n_Γ blocks:

$$\mathcal{B}_\Gamma = \{|\Phi_\mathbf{n}\rangle \in \mathcal{S} : \Gamma(\Phi_\mathbf{n}) = \Gamma\} \tag{87}$$

yielding a block-diagonal Hamiltonian. Each block $\mathbf{H}^\Gamma$ of size $|\mathcal{B}_\Gamma| \times |\mathcal{B}_\Gamma|$ is diagonalised independently:

$$\mathbf{H}^\Gamma \mathbf{c}_I^\Gamma = E_I^\Gamma \mathbf{c}_I^\Gamma \tag{88}$$

All eigenvalues from all blocks are merged and sorted in ascending energy order. Block-local eigenvector indices are converted to global configuration indices via the maintained block-to-global mapping, ensuring all printed state assignments refer to the original global configuration numbering.

7.6 Transition Dipoles and Infrared Intensities

7.6.1 Symmetry Selection Rule

The dipole component $\hat{\mu}_\alpha$ transforms as T_α. The transition dipole $\mu_{\alpha,0I} = \langle\Psi_0|\hat{\mu}_\alpha|\Psi_I\rangle$ is nonzero only if:

$$\Gamma_0 \otimes \Gamma_{T_\alpha} \otimes \Gamma_I \supseteq \Gamma_1 \implies \Gamma_I = \Gamma_{T_\alpha} \tag{89}$$

for transitions from the ground state ($\Gamma_0 = \Gamma_1$).

Table S4: IR-active irreps for the eight supported groups.

Group	IR-active irreps	Translation species
C_1	A	$T_x, T_y, T_z \in A$
C_s	A', A''	$T_x, T_y \in A'$; $T_z \in A''$
C_i	A_u	$T_x, T_y, T_z \in A_u$
C_2	A, B	$T_z \in A$; $T_x, T_y \in B$
C_{2h}	A_u, B_u	$T_z \in A_u$; $T_x, T_y \in B_u$
C_{2v}	A_1, B_1, B_2	$T_z \in A_1$; $T_x \in B_1$; $T_y \in B_2$
D_2	B_1, B_2, B_3	$T_z \in B_1$; $T_y \in B_2$; $T_x \in B_3$
D_{2h}	B_{1u}, B_{2u}, B_{3u}	$T_z \in B_{1u}$; $T_y \in B_{2u}$; $T_x \in B_{3u}$

Selection rules are necessary, not sufficient

A symmetry-allowed transition may still have negligible numerical intensity. A symmetry-forbidden transition has exactly zero intensity regardless of the force field.

7.6.2 Global Normalisation

The dipole operator connects states of different symmetry, so the full $|\mathcal{S}| \times |\mathcal{S}|$ dipole matrix is computed over all pairs with $n_\text{diff} \leq 2$. Both wavefunctions are represented over the full configuration space. Intensities are normalised globally across all blocks:

$$I_{0I}^\text{norm} = \frac{100\,\tilde{I}_{0I}}{\sqrt{\sum_J \tilde{I}_{0J}^2}}, \qquad \tilde{I}_{0I} = \sum_\alpha |\mu_{\alpha,0I}|^2 \tag{90}$$

7.7 Diagnostic Output

The symmetry module produces extensive diagnostic information that is printed to the screen (`stdout`) and to `debug_sym.txt` during the symmetry initialisation phase. This output is intentionally *not* written to the main output file (`vscf.out`) to avoid polluting the production output

with large volumes of per-mode, per-operation data. The diagnostic output serves as the primary tool for verifying that the symmetry assignment is correct and should be inspected whenever the symmetry-adapted VCI is used for the first time on a new molecule or point group.

The following information is printed:

7.7.1 Principal Axis Frame

The principal moments of inertia $I_a \leq I_b \leq I_c$ (in amu Å^2), the 3×3 rotation matrix $\mathbf{P}$ (whose columns are the PA directions expressed in the lab frame), and the PAF coordinates of all atoms. The user should verify that the atomic positions in the PAF are consistent with the expected molecular orientation.

7.7.2 Orientation Detection

For groups with multiple possible axis assignments (C_{2v}, C_{2h}, C_2, C_s), the code tries all candidate orientations and reports the number of atoms correctly mapped for each trial. Example output for a C_{2v} molecule:

```
C2v trial C2=ax 1  sv_n=ax 2  sv_n=ax 3  mapped(C2,sv1,sv2)=  3  7  7  total=   17
C2v trial C2=ax 2  sv_n=ax 1  sv_n=ax 3  mapped(C2,sv1,sv2)=  7  3  7  total=   17
C2v trial C2=ax 3  sv_n=ax 1  sv_n=ax 2  mapped(C2,sv1,sv2)=  7  7  3  total=   17
C2v chosen: C2=ax 3  sv normals: ax 1 ax 2
```

The "chosen" line indicates which PA direction was selected for each symmetry element.

7.7.3 Atom Permutation Table

For each symmetry operation, the code prints:

- The 3×3 operation matrix.
- For each atom: its transformed PAF position, the image atom index, the distance to the image, and a status flag (`SELF` if the atom maps to itself, `->perm` if it maps to a different atom, `*** NO IMAGE ***` if no match was found within δ_{proj}).

A summary permutation table is also printed showing the complete mapping $\hat{R}(a)$ for all atoms and all operations. Any `NO IMAGE` entries indicate that the assigned point group is inconsistent with the molecular geometry and the cutoff δ_{proj}.

7.7.4 Normal Mode Displacement Diagnostic

For every mode (including the six translational/rotational modes), the code prints:

- Lab-frame and PAF displacements for each atom.
- The total squared norm $\|\mathbf{L}^{(k)}\|^2$.
- For each symmetry operation:
  - The operation matrix.
  - For each atom: the original displacement $\mathbf{l}_a^{(k)}$, the rotated displacement $\mathbf{R}\,\mathbf{l}_a^{(k)}$, the contribution $(\mathbf{l}_{\hat{R}(a)}^{(k)})^T \mathbf{R}\,\mathbf{l}_a^{(k)}$ to the numerator, and a flag indicating `SELF` or `PERM`. For permuted atoms, the displacement of the image atom $\mathbf{l}_{\hat{R}(a)}^{(k)}$ is also shown.
  - The accumulated numerator, denominator, and resulting character $\chi^{(k)}(\hat{R})$.

  - A comparison of $\chi^{(k)}(\hat{R})$ against each row of the character table, showing the absolute difference.

This per-atom breakdown allows the user to identify exactly which atoms are contributing to the character and to diagnose problems such as:

- Atoms with no valid image (indicating wrong group or insufficient cutoff).
- Characters deviating significantly from ± 1 (indicating that the operation is not a true symmetry of the molecule).
- Unexpected irrep assignments traceable to specific atomic contributions.

7.7.5 Mode Assignment Summary

For each mode, the assigned irrep, the character vector $[\chi^{(k)}(\hat{R}_1), \ldots, \chi^{(k)}(\hat{R}_{|\mathcal{G}|})]$, and the best-match distance to the character table are printed. Characters that deviate significantly from ± 1 trigger a warning flag. Any mode with a character value in the range $0.1 < |\chi| < 0.9$ triggers a printed warning recommending that the user verify the group assignment or revert to C_1.

7.8 Consequences of an Incorrect Group Assignment

The user specifies the point group directly via the `PGROUP` keyword in `input_vscf.txt`. ⟨Ψ| V_i**Bra** has no mechanism to verify whether the chosen group is consistent with the molecular geometry or the force field. The key principle is that every operation of the assigned group must be a *true* symmetry operation of the molecule, and the code must correctly identify the orientation of each symmetry element in the principal axis frame. When both conditions are met the character computation Eq. (69) produces exact ± 1 values, the block structure is physically meaningful, and the eigenvalues are correct. When either condition is violated the results may be wrong.

Several scenarios can arise, depending on the relationship between the assigned group and the true molecular point group.

7.8.1 Case 1: The Assigned Group Is the True Group

This is the ideal case. All operations are genuine symmetry operations. The orientation detection (which tries all candidate principal axis assignments and selects the one that maps the most atoms) succeeds because the geometry is exactly compatible with the assigned group. The characters are ± 1, the irrep assignments are unambiguous, and the block-diagonal VCI produces correct eigenvalues with full symmetry labels.

7.8.2 Case 2: The Assigned Group Contains Operations That Are Not Symmetry Operations of the Molecule

This occurs when the assigned group is "larger" than the true group (i.e. the true group is a proper subgroup of the assigned group), or when the assigned group is entirely unrelated to the true group. In either case, some operations of the assigned group do not leave the molecule invariant.

The consequences are:

- The atom permutation table may contain incorrect mappings (atoms matched to wrong partners that happen to fall within δ_{proj}) or missing mappings (`NO IMAGE` entries).
- The character computation Eq. (69) produces values that are not ± 1 but take arbitrary values in $[-1, 1]$.

- The minimum-distance irrep assignment Eq. (74) selects some irrep, but this assignment is physically meaningless.
- The block structure has no physical justification: Hamiltonian matrix elements that should be nonzero (because the spurious operation is not a true symmetry) are incorrectly forced to zero by the block assignment.
- Fermi resonances and other couplings between states incorrectly placed in different blocks are missing. The eigenvalues are **wrong**, and the error can be arbitrarily large.

Since ⟨Ψ| V_iBra implements only E, C_2, σ, and i operations, it has no access to higher-order symmetry elements such as C_3, C_4, S_4, or C_6. A molecule whose true group involves such operations (e.g. C_{3v}, T_d, D_{3h}, O_h) cannot be assigned its true group. Assigning any of the eight supported groups that contains operations absent from the true group falls into this case.

7.8.3 Case 3: The Assigned Group Is a Subgroup of the True Group

If the assigned group $\mathcal{G}_{\text{assigned}}$ is a subgroup of the true group $\mathcal{G}_{\text{true}}$, then *in principle* every operation of $\mathcal{G}_{\text{assigned}}$ is a genuine symmetry operation of the molecule. The characters should be exactly ± 1, no physically nonzero coupling is forced to zero, and the eigenvalues should be correct. The only theoretical cost is that the symmetry exploitation is *incomplete*: the block structure is coarser than what the true group would provide, because distinct irreps of the true group may merge into the same irrep of the subgroup.

However, in practice this case is **not guaranteed to work correctly** due to the automatic orientation detection machinery. The code identifies symmetry element orientations by computing the principal axis frame and then trying all candidate axis assignments, selecting the one that maps the maximum number of atoms. For a molecule whose true symmetry is higher than the assigned group, the principal axis frame may not align with the symmetry elements of the subgroup in the way the code expects. For example:

- A C_{3v} molecule (e.g. NH_3) assigned to C_s: the code must identify one of the three equivalent mirror planes. The principal axis frame of a symmetric top places the C_3 axis along one principal direction, but the two perpendicular axes are degenerate and their orientation is determined by the numerical eigenvector routine (`dsyev`), which may or may not align one of them with a mirror plane.
- A D_{3h} molecule assigned to C_{2v}: the molecule has three C_2 axes, but the principal axis frame may not place any of them along a principal axis direction.

If the orientation detection fails to identify the correct symmetry elements, the atom permutation table will be incorrect and the characters will not be ± 1, even though valid subgroup operations exist.

Subgroup assignments require verification

Assigning a supported Abelian subgroup of the true molecular point group *can* produce correct results but is **not guaranteed** to do so. The automatic orientation detection may fail for molecules with symmetry higher than the assigned group, particularly when degenerate principal moments of inertia leave the principal axis frame under-determined.

The user **must** verify any subgroup assignment by comparing the eigenvalues against a C_1 reference calculation. If the eigenvalues agree within numerical precision (typically < 0.1 cm^{-1}), the subgroup assignment is working correctly and provides partial symmetry labels. If they disagree, the assignment has failed and C_1 should be used.

7.8.4 C_1: The Unconditionally Safe Choice

Setting $\mathcal{G}_{\text{assigned}} = C_1$ disables all symmetry blocking. The single trivial operation E is always a valid symmetry operation, the single irrep A is assigned to every mode and every configuration, and the full unsymmetrised VCI Hamiltonian is diagonalised as a single block. The eigenvalues and intensities are always correct. The only costs are the loss of symmetry labels on the eigenstates and the absence of the computational speedup from block diagonalisation.

C_1 should always be used as the reference against which any non-trivial group assignment is validated.

7.8.5 Summary

Table S5: Summary of consequences of different group assignments. "Correct" means identical to the exact (unsymmetrised) VCI Hamiltonian eigenvalues.

**Assignment**	**Energies**	**Intensities**	**Labels**
True group (if supported)	Correct	Correct	Exact
Supported subgroup of true group	Correct if orientation detection succeeds (**verify vs.** C_1)	Correct if orientation detection succeeds	Partial (coarser blocks)
C_1	Correct (always)	Correct (always)	None
Group containing operations not in true group	**Wrong:** missing couplings between incorrectly separated blocks	**Wrong**	**Meaningless**

7.8.6 Diagnosis of an Incorrect Assignment

Because ⟨Ψ| V_iBra cannot verify the group assignment automatically, the user must check the output for diagnostic signs of an incorrect assignment:

- **Non-integer characters in diagnostic output.** The mode character values $\chi^{(k)}(\hat{R})$ are printed during initialisation (to `stdout`) and and in summary form (to `debug_sym.txt`). Values significantly different from ± 1 indicate that the assigned operation is not a true symmetry operation of the molecule, or that the orientation detection has failed. In the production output, any character with $0.1 < |\chi| < 0.9$ triggers an explicit warning. Adjusting `PROJCT` will not resolve the issue if the root cause is a wrong group assignment or a failed orientation detection rather than a numerical threshold problem.
- **`NO IMAGE` entries in the permutation table.** These indicate that one or more atoms have no valid partner under the assigned operation, which is a definitive sign that the operation is not a symmetry of the molecule (or that the orientation is wrong).
- **Unphysical block sizes.** For a molecule with true symmetry $\mathcal{G}$, each vibrational irrep appears with a multiplicity determined by the reduction formula. Wildly unequal block sizes (e.g. one block containing nearly all configurations, others nearly empty) suggest that the character computation is not producing ± 1 values, which in turn causes almost all configurations to be assigned the same "lowest-distance" irrep.
- **Energies inconsistent with C_1 results.** This is the most reliable diagnostic. The eigenvalues from the symmetry-adapted VCI must equal those from the standard (unsymmetrised) VCI

run with C_1. Any discrepancy beyond numerical noise ($\gtrsim 0.1$ cm^{-1}) indicates that nonzero Hamiltonian elements have been incorrectly forced to zero by a wrong block structure. **The user should always perform this comparison when using a non-trivial group assignment for the first time.**

- **Violation of known selection rules.** If a mode that is known (from the electronic structure calculation) to be IR-forbidden appears with nonzero intensity, or vice versa, the group assignment is likely incorrect.

Recommended workflow for symmetry assignment

1. Run the calculation with C_1 to obtain reference eigenvalues - Use maybe less quanta.
2. Run with the desired point group.
3. Inspect the `stdout` and `debug_sym.txt` diagnostic output: check that all characters are close to ± 1 and that no `NO IMAGE` warnings appear.
4. Compare the eigenvalues from steps 1 and 2. Agreement within ~ 0.1 cm^{-1} confirms that the symmetry assignment is correct.
5. If the eigenvalues disagree, revert to C_1 or try a lower-symmetry subgroup.

8 Output File Formats

8.1 vscf.out — Complete Calculation Log

This master output file vscf.out contains every stage of the calculation in sequential order.

8.1.1 VSCF Ground-State Block

The ground-state block records the converged VSCF modal coefficient matrix for every mode. Each matrix has columns indexed by quantum number $n_i = 0, 1, \ldots$ and rows by basis function index $\mu = 1, \ldots, N_{\text{exp}}$. These matrices define the transformation from primitive harmonic oscillator functions to the VSCF-optimised modals and are stored internally as full_coef(mode, state, basis).

```
----------------------------------------
WORKING ON STATE     0     0     0
----------------------------------------
Coefficient matrix for mode:          1
 ---
         0          1          2          3          4
   0.99821   -0.04182    0.00312    0.00025   -0.00003
   0.04180    0.99893   -0.00156    0.00089    0.00012
  -0.00326    0.00143    0.99987   -0.02145    0.00078
   0.00024   -0.00087    0.02144    0.99976   -0.01234
   0.00003    0.00011   -0.00076    0.01233    0.99992

Coefficient matrix for mode:          2
 ---
         0          1          2          3          4
   0.99756   -0.05234    0.00456    0.00034   -0.00004
   0.05231    0.99821   -0.00234    0.00112    0.00015
  -0.00468    0.00221    0.99978   -0.01876    0.00067
   0.00033   -0.00109    0.01875    0.99982   -0.01056
   0.00004    0.00014   -0.00065    0.01055    0.99994

Coefficient matrix for mode:          3
 ---
(similar block)

STATE ENERGY (cm-1):      4660.3717
TRANSITION ENERGY (cm-1):     0.0000
```

Listing 1: Ground-state VSCF block (3 modes, 5 basis).

These coefficient matrices are printed *only* for the ground state (the flag write_on_out is set to 1 only for $\mathbf{n} = \mathbf{0}$). They are used by the VCI/SCI stage to construct the modal integral lookup table.

8.1.2 VSCF Excited-State Blocks

For each fundamental excitation $\mathbf{n} = \mathbf{e}_i$, the file shows the iteration-by-iteration convergence:

```
----------------------------------------
WORKING ON STATE     1     0     0
----------------------------------------
ENERGY(cm-1)         DELTA E
      5832.452100      5832.452100
      5831.189300        -1.262800
      5830.754600        -0.434700
      5830.742200        -0.012400
      5830.742100        -0.000100
>>>>>>>> CONVERGENCE REACHED
STATE ENERGY (cm-1):      5830.7421
TRANSITION ENERGY (cm-1):  1170.3704
 ---
HARMONIC ENERGY  1:  1627.4500
```

Listing 2: VSCF fundamental of mode 1.

Each convergence line shows the current total VSCF energy and its change from the previous iteration. The harmonic energy is printed for direct comparison with the anharmonic VSCF transition energy.

8.1.3 VCI Configuration List

```
state      1: (0,0,0,0,0,0)
state      2: (1,0,0,0,0,0)
state      3: (0,1,0,0,0,0)
...
state      7: (0,0,0,0,0,1)
state      8: (2,0,0,0,0,0)
state      9: (1,1,0,0,0,0)
...
 States generated:          792
```

Listing 3: Configuration list (6-mode excerpt).

8.1.4 SCI Selection Report

```
CISD reference:       91 configs out of       792
--- CISD energies (cm-1, relative to ZPE):
     1       4660.3717           0.0000
     2       5830.7189        1170.3472
     ...
 -->  147 new configs selected (union across 10 states)
Final active space:   238 configurations
  (CISD: 91 + selected: 147)
```

Listing 4: SCI report.

8.1.5 VCI/SCI Eigenstate Table

```
=============================================
  FINAL VCI ENERGIES
=============================================
Final active space size:      238
-----------------------------------------------
  E (cm-1)    E - ZPE      || Coeff A    Coeff B    Coeff C  St A   St B   St C
-----------------------------------------------
  4660.3717     0.0000     || 0.998271  0.041820  -0.024610    1    44    24
  5830.7422  1170.3705     || 0.990614  -0.078542  0.059132    2    56     7
  ...
-----------------------------------------------
```

Listing 5: Eigenstate table.

"State" indices refer to the configuration list.

8.1.6 Timing

```
H build CPU time:        12.34 seconds
H build elapsed time:     3.21 seconds
SCI CPU time:            18.92 seconds
Total elapsed time:      10.77 seconds
```

Listing 6: Timing.

8.2 `intensities.txt`

```
HO
6
  1627.450000       25.123456
  3785.230000       42.567890
  ...
VSCF
6
  1598.120000       23.456789
  ...
VCI
15
  1170.370500       12.345678
  ...
```

Listing 7: `intensities.txt` format.

Each block: method label, count N, then N lines of (frequency cm^{-1}, normalised intensity %). Normalisation: $100 \times I_i / \sqrt{\sum_j I_j^2}$.

Dipole order per block

HO and VSCF: first-order dipole only. VCI: first + second order.

8.3 normal_mode.txt

```
3
      O       0.000000    0.000000    0.117300
      H       0.000000    0.757200   -0.469200
      H       0.000000   -0.757200   -0.469200
Mode   1
  -0.001234
   0.000000
   0.045678
   ...
Mode   7
1627.450000
   0.012345
  -0.067890
   ...
```

Listing 8: `normal_mode.txt` (water).

Line 1: atom count. Then coordinates (Å). For each of the $3N_{\mathrm{at}}$ modes: header, frequency (cm^{-1}), $3N_{\mathrm{at}}$ displacement components. First 5 or 6 modes are translational/rotational.

9 Auxiliary Fortran Modules

Module inventory

`main.f90`
Entry point and orchestration layer for the execution workflow.

`read_input.f90`
Keyword-based input parser with validation of user-specified options.

`read_orca.f90`
Parser for ORCA `.vpt2` output files. This module performs Hessian diagonalization, handles dipole transformations, and writes `normal_mode.txt`.

`get_combination.f90`
Configuration-enumeration routines, including recursive stars-and-bars combinatorial counting, unique-element decomposition, and degeneracy-factor evaluation.

`integrals.f90`
Analytical harmonic-oscillator matrix elements used in the construction of the vibrational Hamiltonian.

`one_mode_operation.f90`
VSCF mean-field routines.

`vci.f90`
Central VCI kernel. This module contains the shared `compute_H_element` routine and supports full VCI, selected VCI with EN-PT2 screening, symmetry-adapted VCI, and dipole-property calculations.

`jacobi.f90`
LAPACK-based eigensolver wrappers, including `DSYEVD` and `DSYEVR`, with automatic workspace queries and eigenvector normalization.

`symmetry.f90`
Symmetry-handling routines, including point-group character tables, symmetry operations, direct-product tables, normal-mode irrep assignment by principal-axis-frame projection, and block-diagonal VCI dispatch.

10 Input Variables

Table S6: Principal input keywords of ⟨Ψ| V_iBra.

Keyword	Type	Description
NMODES	int	Number of vibrational modes (M).
NEXPAN	int	HO basis size per mode (N_{exp}).
FILECT	str	Path to ORCA `.vpt2` file.
CTEMOD	str	Format (`orca_vpt2`).
NQUANT	int	Maximum total quanta (N_q); ≤ 0 disables VCI.
NSTATE	int	Number of eigenstates (≤ 0 = all).
CVGSCF	int	VSCF convergence exponent (10^{-C} cm^{-1}).
THREAD	int	Number of OpenMP threads.
PGROUP	str	Point group (C1, Cs, Ci, C2, C2h, C2v, D2, D2h).
PROJCT	real	Projection cutoff for symmetry detection (Å).
MAXSCI	int	N_{sel} for S-VCI. Default: 100.
		Usage: `MAXSCI [N] [mode] [ref]`
		`N > 0, mode = auto`: iterative selection with CI reference (`ref` = `s`, `d`, `t`, `q`).
		`N > 0, mode = list`: iterative selection with list reference, no `ref` needed.
		`N = 0`: full VCI (no S-VCI), no `mode` and no `ref` needed.
		`N = 0, mode = list`: full VCI using user-provided state list, no `ref` needed.

Example `input_vscf.txt` with all keywords:

```
! ============================================================
!  VCI@VSCF input file
! ============================================================

NMODES   9
NEXPAN   10
FILECT   molecule.vpt2
CTEMOD   orca_vpt2
NQUANT   4
NSTATE   500
CVGSCF   6
THREAD   4
PGROUP   C2h
PROJCT   0.05
MAXSCI   200 auto d
```

11 Main Program Workflow

1. **Initialisation:** read input (including `PGROUP`, `PROJCT`, `MAXSCI`); parse ORCA file; apply degeneracy factors; convert to atomic units; precompute HO integrals.
2. **VSCF:** converge ground state (store modals); converge each fundamental; write HO and VSCF blocks to output files.
3. **Symmetry initialisation:** if `PGROUP` ≠ `C1` and `normal_mode.txt` is present, write `point_group.txt` from `PGROUP`, call `init_symmetry` with the projection cutoff `PROJCT`, assign irreps to all modes.
4. **VCI/SCI dispatch:**
   - If symmetry is active ⇒ symmetry-adapted VCI.
   - Else if `MAXSCI` > 0 ⇒ Selected VCI with at most `MAXSCI` iterations.
   - Else ⇒ standard full VCI.

   Build, diagonalise, compute dipoles; write VCI block.

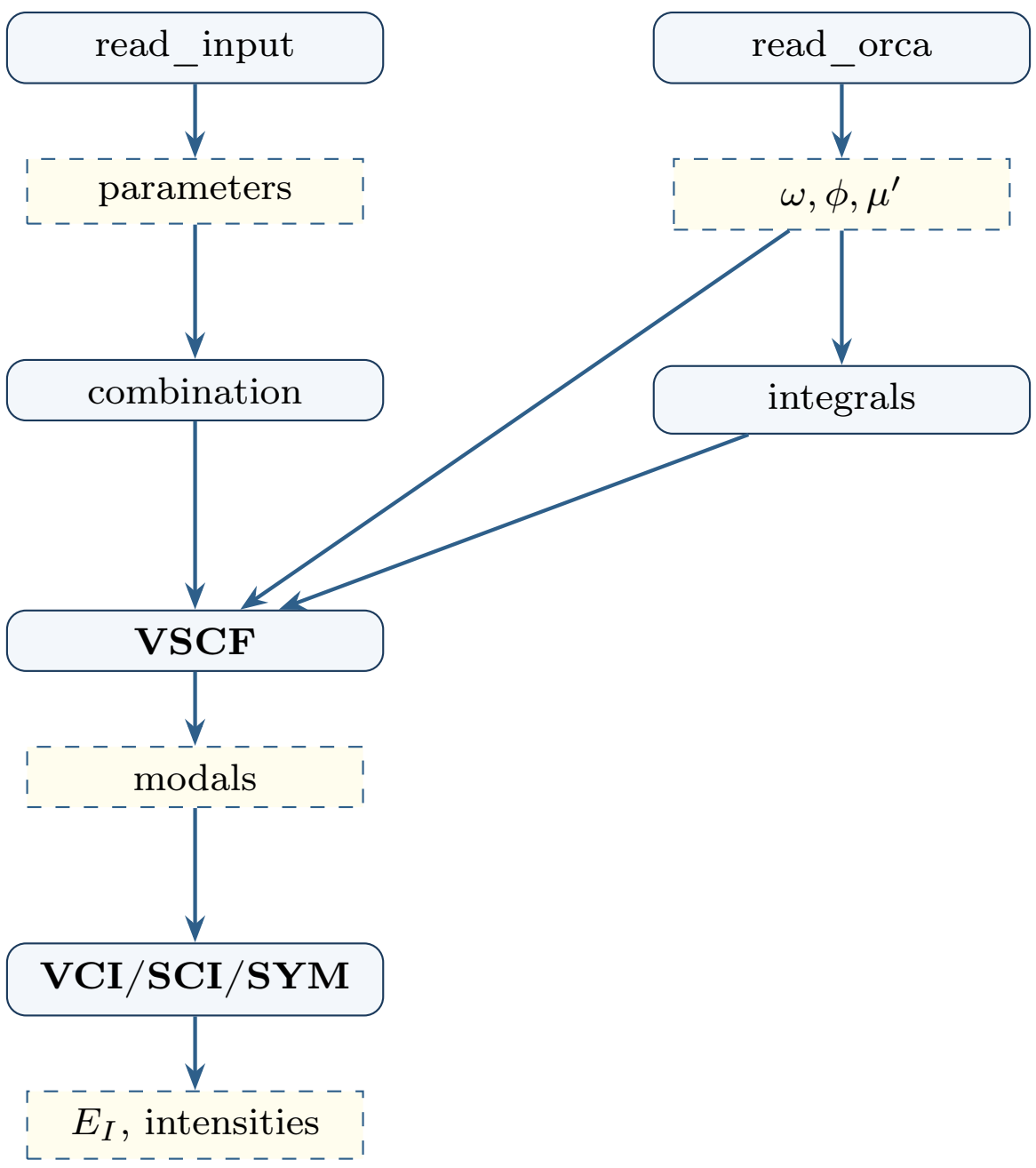

Figure S2: Data flow in the Fortran engine. The VCI dispatch (**VCI/SCI/SYM**) is controlled by `PGROUP` and `MAXSCI`.

12 Graphical User Interface

The GUI (`VSCFVCIApp` class, Python 3, CustomTkinter) provides:

1. **File selection**: browse dialog sets the working directory to the `.vpt2` file's location.
2. **Input creator**: modal dialog with all keywords, tooltips, and validation.
3. **Job execution**: copies executable and libraries to the working directory, runs as a subprocess in a background thread, streams stdout to the output panel in real time, cleans up copied files on completion or cancellation.
4. **Stop**: terminates a running process.
5. **Output panel**: scrollable text with save and clear.
6. **Visualisation launch**: starts the spectral viewer executable.

12.1 Spectral Viewer and Normal-Mode Animator

The viewer (`MainApplication` class, Python 3) reads the three output files and provides interactive spectral analysis and 3D animation.

12.2 Spectral Display

Each transition at frequency ν_k with intensity I_k is broadened with a Gaussian:

$$S(\nu) = \sum_k I_k \exp\!\Big(-\frac{(\nu-\nu_k)^2}{2\sigma^2}\Big), \qquad \sigma = \frac{\text{FWHM}}{2\sqrt{2\ln 2}} \tag{91}$$

For $T > 0$, VCI intensities are Boltzmann-weighted:

$$p_k = \frac{e^{-hc\nu_k/k_BT}}{Z}, \qquad I_k^{\text{eff}} = I_k\,(p_0 - p_k)/100 \tag{92}$$

with $hc/k_B = 1.438777$ cm·K.

Controls: temperature (0–950 K, stepper + direct entry), FWHM (stepper + direct entry), intensity cut threshold, X-range sliders, per-method visibility for spectra and peak labels, VCI population display.

12.3 JCAMP-DX Overlay

Experimental spectra in JCAMP-DX format (`.jdx`/`.dx`) can be loaded and overlaid. Transmittance is automatically converted to absorbance, and the spectrum is L2-normalised.

12.3.1 Asymmetric Least Squares (ALS) Baseline Correction

Experimental IR spectra often exhibit a sloping baseline from instrumental drift, scattering, or sample artefacts. ⟨Ψ| V_iBra implements the ALS method to estimate and subtract this baseline before comparison with computed spectra.

12.3.2 Formulation

Given a spectrum $\mathbf{y} \in \mathbb{R}^N$, ALS estimates a smooth baseline $\mathbf{z}$ by minimising:

$$Q(\mathbf{z}) = \sum_{i=1}^{N} w_i(y_i - z_i)^2 + \lambda \|\mathbf{D}\mathbf{z}\|^2 \tag{93}$$

where $\mathbf{D}$ is the $(N-2) \times N$ second-difference matrix (so $\|\mathbf{D}\mathbf{z}\|^2$ penalises curvature), $\lambda > 0$ controls smoothness, and the weights w_i are updated iteratively:

$$w_i = \begin{cases} p & \text{if } y_i > z_i \quad \text{(above: likely a peak)} \\ 1-p & \text{otherwise} \quad \text{(below: likely background)} \end{cases} \tag{94}$$

with $0 < p \ll 1$. Setting $\partial Q/\partial \mathbf{z} = 0$ gives the linear system solved at each iteration:

$$\left(\mathbf{W} + \lambda\, \mathbf{D}^T\mathbf{D}\right) \mathbf{z} = \mathbf{W}\, \mathbf{y} \tag{95}$$

where $\mathbf{W} = \mathrm{diag}(w_i)$. The coefficient matrix is symmetric positive definite and pentadiagonal, solved in $O(N)$ by SciPy's sparse solver.

12.3.3 Parameters

- λ (default 10^6): large → flat baseline; small → follows curvature.
- p (default 0.001): small → aggressive correction (baseline hugs the bottom); large → conservative.
- 10 iterations (fixed); convergence is typically complete.

After subtraction ($y_i^{\text{corr}} = y_i - z_i$), negative values are clipped to zero and the result is renormalised to a 0–100 scale. The correction is optional (toggled by a checkbox) and the original data is preserved for immediate reversal. Both λ and p are adjustable via interactive steppers with real-time redisplay.

12.4 VCI Transition Details

Clicking a VCI peak label displays: frequency, intensity, the three largest CI coefficients with their quantum number assignments (e.g. "$v_1 + 2v_3$"), and buttons to launch 3D animations of each excited mode. This information is parsed from `vscf.out`.

12.5 3D Normal-Mode Animation

Using `normal_mode.txt`, the viewer generates sinusoidal displacement trajectories (60 frames, configurable amplitude), saves them as JSON, generates an HTML file with embedded 3Dmol.js viewer (play/pause, frame slider, speed, rendering style, adjustable radii), and opens it in the system browser.

12.6 Data Export

The save function exports a text file with all currently displayed spectral data and a 300 dpi PNG of the plot.

13 Computational Optimisation Strategies

This section describes the optimisation techniques employed in ⟨Ψ| V_iBra to make the Hamiltonian construction and property calculation tractable for molecules with many modes. We pay particular attention to why certain strategies benefit one part of the calculation but not another, since the mean-field (VSCF) and configuration-interaction (VCI) steps have fundamentally different computational structures.

13.1 Overlap Cache: Eliminating Redundant Basis Summations

In the VSCF mean-field equations (Section 4) the effective potential for mode i contains scalar overlaps of the form

$$S_j^{(p)} = \sum_{\mu,\nu=0}^{N_{\rm exp}-1} C_{j,\mu}\, C_{j,\nu}\, \mathcal{I}_j(\mu,\nu;\,p)\,, \qquad p = 0,\ldots,4\,, \tag{96}$$

where $C_{j,\mu}$ are the current modal coefficients for mode j and $\mathcal{I}_j(\mu,\nu;\,p)$ are the primitive harmonic oscillator matrix elements (Section 3). Without caching, this double sum must be evaluated for every potential term, every working mode, and every SCF iteration. The resulting cost per iteration is

$$\mathcal{O}\big(M \times T \times P_{\rm max} \times N_{\rm exp}^2\big)\,, \tag{97}$$

where T is the total number of nonzero potential terms ($T = T_3 + T_4$, cubic plus quartic) and $P_{\rm max}$ is the maximum power when evaluating the integrals.

By precomputing Eq. (96) once at the beginning of each SCF iteration, the cost of filling the cache is only

$$\mathcal{O}\big(M \times 5 \times N_{\rm exp}^2\big) \quad \text{per iteration}\,, \tag{98}$$

and every subsequent potential-term evaluation reduces to a single array lookup $S_j^{(p)}$ instead of an $N_{\rm exp}^2$ double sum.

13.2 Vectorised Potential Storage and Nonzero Flags

The cubic and quartic force constants are stored in upper-triangular form ϕ_{ijk} $(i \le j \le k)$ and ϕ_{ijkl} $(i \le j \le k \le l)$ as one-dimensional vectors of length

$$T_3 = \binom{M+2}{3}, \qquad T_4 = \binom{M+3}{4}. \tag{99}$$

For each entry, the following metadata is precomputed once during initialisation:

- **Unique mode indices**: the distinct mode labels appearing in the term.
- **Multiplicities**: the power of each unique mode (e.g. for $\phi_{1,1,2}$ the unique modes are $\{1, 2\}$ with multiplicities $\{2, 1\}$).
- **Nonzero flag**: set to true if $|\phi| > 10^{-26}$, false otherwise.

Many force constants are zero or negligibly small. The nonzero flags allow these to be skipped with a single integer comparison, avoiding the cost of loading the potential value and associated index arrays from memory. This vectorised representation is computed once before both the VSCF and VCI stages and is shared between them.

13.3 The Inverted Index: Why It Helps VCI but Not VSCF

The inverted index maps each mode m to the list of potential terms in which it participates. Understanding why this data structure is essential for VCI but irrelevant for VSCF requires examining how potential terms contribute in each formalism.

13.3.1 VSCF: every term contributes to every mode

In VSCF the effective one-mode Hamiltonian for mode i (Eq. 25) collects contributions from all potential terms, distributed across the polynomial coefficients $X_i^{(0)}, \ldots, X_i^{(4)}$. Consider a cubic term $\tilde{\phi}_{a,b,c}$ with $a \leq b \leq c$. For a given working mode i there are two cases:

1. **Mode i appears in the term** ($i \in \{a, b, c\}$): the modes other than i are contracted into scalar overlaps using the current coefficients, and the remaining power on mode i determines the slot $X_i^{(q)}$ that receives the contribution ($q > 0$).

2. **Mode i does not appear in the term** ($i \notin \{a, b, c\}$): all three modes are contracted as scalars, and the result is a constant energy shift accumulated into $X_i^{(0)}$, which multiplies the overlap integral $\mathcal{I}_i(\mu, \nu; 0)$ in the Hamiltonian matrix.

Crucially, *both* cases produce a nonzero contribution. Every potential term affects every mode—either as an operator ($q > 0$) or as a constant shift ($q = 0$). The inverted index lists only terms where mode i appears (case 1), so using it would miss all the $q = 0$ contributions from case 2.

One might attempt to split the calculation: use the inverted index for $q > 0$ terms, and handle $q = 0$ separately. However, the $q = 0$ pass already requires scanning all T terms for each working mode (to identify the terms where mode i is absent), recovering the same $\mathcal{O}(M \times T)$ cost as the direct loop. The inverted-index pass for $q > 0$ then becomes *additional* work on top of this, making the total cost strictly greater than the single-pass approach.

⟨Ψ| V_iBra therefore uses a direct sequential loop for VSCF: for each potential term, the fully contracted scalar and the per-mode contributions are computed simultaneously in a single pass. Combined with the overlap cache (Section 13.1), this makes the VSCF step extremely efficient—typically completing in milliseconds per iteration.

13.3.2 VCI: most terms are irrelevant for a given pair

In VCI a Hamiltonian matrix element between two multi-mode configurations $|\boldsymbol{v}_\alpha\rangle$ and $|\boldsymbol{v}_\beta\rangle$ involves a sum over potential terms. Each term contributes a product of one-mode integrals that factorises over all M modes. To see precisely where most terms vanish, consider a quartic term $\tilde{\phi}_{ijkl}$ with four *distinct* modes i, j, k, l (all different). Since each mode appears exactly once, each carries power $p = 1$. The contribution to $H_{\alpha\beta}$ is

$$\tilde{\phi}_{ijkl}\, \mathcal{I}_i(v_{\alpha,i},\, v_{\beta,i};\, 1)\, \mathcal{I}_j(v_{\alpha,j},\, v_{\beta,j};\, 1)\, \mathcal{I}_k(v_{\alpha,k},\, v_{\beta,k};\, 1)\, \mathcal{I}_l(v_{\alpha,l},\, v_{\beta,l};\, 1) \prod_{m \notin \{i,j,k,l\}} \mathcal{I}_m(v_{\alpha,m},\, v_{\beta,m};\, 0) \tag{100}$$

The product runs over all *spectator* modes—those not involved in the potential term. Spectator modes always enter with power $p = 0$ because, by definition, the potential term does not contain the coordinate Q_m for any spectator mode m.

More generally, when indices repeat (e.g. $\tilde{\phi}_{iijk}$ with unique modes $\{i, j, k\}$ and multiplicities $\{2, 1, 1\}$), the powers on the active modes reflect the multiplicity: mode i carries $p_i = 2$ while modes j and k carry $p = 1$. The spectator modes still enter with $p = 0$.

For a spectator mode m where the two configurations have the *same* quantum number ($v_{\alpha,m} = v_{\beta,m} = v$), the factor is $\mathcal{I}_m(v, v; 0)$, which in the VSCF modal basis is simply the norm of the v-th

modal. Since the VSCF modals are normalised within each mode's eigenvalue problem, this equals unity:

$$\mathcal{I}_m(v, v;\, 0) = \langle \tilde{\varphi}_m^{(v)} | \tilde{\varphi}_m^{(v)} \rangle = 1 \tag{101}$$

These factors contribute trivially and need not be computed.

Now consider a spectator mode m where the two configurations *differ* ($v_{\alpha,m} \neq v_{\beta,m}$). Because the potential term does not act on mode m ($p = 0$), the factor is the overlap between two *different* eigenstates of the same VSCF effective Hamiltonian:

$$\mathcal{I}_m(v_{\alpha,m},\, v_{\beta,m};\, 0) = \langle \tilde{\varphi}_m^{(v_\alpha)} | \tilde{\varphi}_m^{(v_\beta)} \rangle = \delta_{v_\alpha, v_\beta} = 0 \tag{102}$$

Since the modals of the same mode are orthogonal (they are eigenvectors of the same Hermitian operator), this overlap vanishes exactly. A single vanishing factor zeros the entire product in Eq. (100), killing the contribution of the term.

This is the mechanism behind the selection rule: a potential term can produce a nonzero off-diagonal matrix element $H_{\alpha\beta}$ only if it *touches* (has nonzero power on) every mode where α and β differ. Otherwise, at least one spectator factor is an orthogonality zero, and the entire product vanishes. For a quartic force field, at most four modes can be touched by any single term, so configurations differing in more than four modes are automatically decoupled.

For a pair (α, β) that differs in d modes (typically $d = 1$ or 2), only the potential terms involving *at least those d specific modes* survive. A naïve approach would check all $T_3 + T_4$ terms, but the inverted index allows:

1. Looking up the list of terms involving the first differing mode (typically $O(M)$ entries out of $O(M^3)$ or $O(M^4)$ total).

2. For each candidate, verifying that it also involves the remaining differing modes (a cheap check on the precomputed unique-mode lists).

This reduces the number of potential-term evaluations per off-diagonal pair from $O(T)$ to $O(T/M^{d-1})$ on average, which for $M = 30$ and $d = 2$ represents a factor-of-30 reduction.

For diagonal elements ($d = 0$) all terms contribute—exactly as in VSCF—so the inverted index provides no benefit there. However, diagonal elements are a tiny fraction of the total number of coupled pairs in the sparse Hamiltonian, so the overall speedup is substantial.

Table S7: Comparison of potential-term scanning strategies in VSCF and VCI. Here d denotes the number of modes in which two configurations differ.

	**VSCF**	**VCI (off-diagonal)**
Terms scanned per mode/pair	All T	$O(T/M^{d-1})$
Reason	Every term produces a $q \geq 0$ contribution	Spectator overlaps vanish by modal orthogonality
Key identity	$S_j^{(p)} \neq 0$ always	$\langle \tilde{\varphi}^{(v)} \| \tilde{\varphi}^{(v')} \rangle = \delta_{vv'}$
Inverted index useful?	No	Yes

13.4 Merged $\bar{V}_c$ and Mean-Field Passes

The VSCF energy (Eq. 34) contains the fully averaged coupling potential $\bar{V}_c$ (Section 4.8) to prevent double-counting of mean-field interactions. Since $\bar{V}_c$ is the fully contracted scalar product of all mode overlaps weighted by the potential constant—the same information needed for the $q = 0$ contributions to $X_i^{(0)}$—the two calculations are merged into a single pass over each potential type.

For each potential term the algorithm:

1. Computes the fully contracted product $\prod_j S_j^{(p_j)}$ using the overlap cache.

2. Checks whether the term is a $\bar{V}_c$ contributor (no single mode carries all the power) and, if so, accumulates it into $\bar{V}_c$.

3. Distributes the per-mode contributions into the appropriate $X_i^{(q)}$ slots for all M working modes.

This halves the number of passes over the potential data compared to computing $X_i^{(q)}$ and $\bar{V}_c$ in separate loops.

13.5 Sparse Pair List for VCI

The selection rule $n_{\text{diff}} \leq 4$ (Section 3) means that the VCI Hamiltonian matrix is extremely sparse for large configuration spaces. The fraction of coupled pairs decreases as $|\mathcal{S}|$ grows, since most pairs of states differ in more than four modes.

Building the sparse pair list requires two passes through the upper-triangular index space $\alpha \leq \beta$:

1. **Counting pass**: determine the number of pairs with $n_{\text{diff}} \leq 4$, allowing exact memory allocation.

2. **Filling pass**: record the pair indices (α, β), the number of differing modes n_{diff}, and the identities of the differing modes.

Storing the differing-mode information at pair-construction time avoids recomputing it during the Hamiltonian loop, where it is needed both for the inverted-index lookup and for selecting which modal integrals to evaluate.

13.6 Prefix–Suffix Overlap Products

Many VCI Hamiltonian contributions require the product of one-mode overlaps for all modes *except* a small subset (Section 5). Computing this naïvely as a product over $M - d$ modes costs $O(M)$ per evaluation and $O(M^2)$ over all modes. The prefix–suffix technique replaces this with a single $O(M)$ precomputation:

1. Compute the running product from the left: $\text{prefix}(k) = \prod_{m=1}^{k} o_m$.

2. Compute the running product from the right: $\text{suffix}(k) = \prod_{m=k}^{M} o_m$.

3. The product excluding mode k is $\text{prefix}(k-1) \times \text{suffix}(k+1)$.

where $o_m = \mathcal{I}_m(v_{\alpha,m},\, v_{\beta,m};\, 0)$ is the overlap integral for mode m between the two configurations.

As discussed in Section 13.3.2, for spectator modes with equal quantum numbers $o_m = 1$, while for spectator modes with differing quantum numbers $o_m = 0$ by modal orthogonality. The prefix–suffix structure naturally propagates these zeros: if any factor vanishes, all exclude-one products that do not exclude that mode will also vanish, and the algorithm detects this without explicit checking.

For multi-mode exclusion (excluding modes $k_1, \ldots, k_d$ with $d \leq 4$), the full product $\Pi = \prod_m o_m$ is divided by the excluded overlaps when no overlap vanishes. A fallback explicit product handles the case where any overlap is zero. A threshold of 10^{-30} on the product magnitude allows early termination of contributions that are numerically negligible.

13.7 OpenMP Parallelism

Table S8 summarises the parallelisation points. The VSCF module runs entirely in serial: with the overlap cache (Section 13.1) and merged passes (Section 13.4), each SCF iteration completes in milliseconds for typical systems, and thread-management overhead would dominate. Parallelism is reserved for the VCI stage, where the workload is orders of magnitude larger.

Table S8: OpenMP parallelisation points in ⟨Ψ| V_iBra.

Stage	Loop	Schedule
VCI/SCI	Modal integral precomputation	static, collapsed
VCI/SCI	Hamiltonian construction (sparse pairs)	dynamic, 64–256
SCI	Epstein–Nesbet PT2 screening	dynamic, 16
VCI/SCI	Dipole matrix construction	dynamic, 64
VCI/SCI	Dipole eigenvector transformation	static

Dynamic scheduling is chosen for the Hamiltonian construction loop because diagonal pairs ($n_{\text{diff}} = 0$) evaluate all nonzero potential terms while high-n_{diff} pairs evaluate far fewer, creating significant load imbalance. The chunk sizes (64, 256, 16) are tuned empirically to balance scheduling granularity against overhead.

The main program sets the linear algebra library and OpenMP to single-threaded during the sequential VSCF phase and switches to the user-specified thread count for VCI/SCI.

13.8 BLAS and LAPACK Usage

Matrix operations use optimised BLAS/LAPACK routines:

- **Matrix–vector product** (DGEMV): used for the dipole eigenvector transformation $\mathbf{t}_\alpha = \boldsymbol{\mu}_\alpha \cdot \mathbf{c}_0$, with cost $O(|\mathcal{S}|^2)$ per Cartesian component.
- **Dot product** (DDOT): used for each transition dipole moment $\langle \Psi_I | \hat{\mu}_\alpha | \Psi_0 \rangle = \mathbf{c}_I^T \cdot \mathbf{t}_\alpha$, with cost $O(|\mathcal{S}|)$ per state per component.
- **Divide-and-conquer eigensolver** (DSYEVD): used for the VSCF modal problems ($N_{\text{exp}} \times N_{\text{exp}}$ matrices) and for full VCI when all eigenvalues are requested.
- **Relatively robust representation eigensolver** (DSYEVR): used for VCI/SCI when only the lowest N_{states} eigenvalues and eigenvectors are needed, significantly faster than a full decomposition for partial spectra. Automatic workspace sizing is used in both eigensolvers.

13.9 SCI as Computational Reduction

The selected CI method (Section 6) is itself the most impactful optimisation for large systems. By replacing the full $|\mathcal{S}| \times |\mathcal{S}|$ Hamiltonian with a much smaller $|\mathcal{S}_{\text{active}}| \times |\mathcal{S}_{\text{active}}|$ matrix, both the construction and diagonalisation costs are dramatically reduced. The Hamiltonian construction scales as $O(|\mathcal{S}_{\text{active}}|^2)$ (or better with sparsity), and the diagonalisation as $O(|\mathcal{S}_{\text{active}}|^2 \times N_{\text{states}})$ with the partial eigensolver. Since $|\mathcal{S}_{\text{active}}| \ll |\mathcal{S}|$ for well-chosen thresholds ϵ_{SCI}, the overall wall time can be reduced by orders of magnitude while preserving accuracy for the lowest-lying states of interest. The maximum number of expansion–diagonalisation cycles is bounded by `MAXSCI` (Section **??**), giving the user direct control over the cost–accuracy trade-off.

13.10 Symmetry-Adapted VCI as a Cost-Reduction Strategy

For molecules whose true point group belongs to the set $\{C_1, C_s, C_i, C_2, C_{2h}, C_{2v}, D_2, D_{2h}\}$, block-diagonalising the Hamiltonian by irrep provides a complementary and exact cost reduction. If the $|\mathcal{S}|$ configurations split into n_Γ blocks of sizes $\{d_\Gamma\}$, the diagonalisation cost is reduced from $\mathcal{O}(|\mathcal{S}|^3)$ to $\sum_\Gamma \mathcal{O}(d_\Gamma^3)$, yielding an exact factor of n_Γ^2 for equally populated blocks (16× for D_{2h}, 16× for C_{2v}). The point group is supplied via the `PGROUP` keyword and the numerical tolerance for the irrep assignment via `PROJCT` (Section 7.3.8). Unlike SCI, no configurations are discarded and no perturbative approximation is introduced: the symmetry-adapted VCI is *exactly* equivalent to the full VCI for the correct group assignment, with strictly lower computational cost. The two strategies are complementary: SCI reduces the configuration space by discarding unimportant basis functions, while VCI-SYM reduces the effective matrix size by enforcing exact selection rules.

13.11 Memory Considerations

The full VCI Hamiltonian is stored as a dense symmetric matrix, which limits the practical configuration space size to $|\mathcal{S}| \sim 10^4$–10^5 depending on available memory (the matrix requires $|\mathcal{S}|^2 \times 8$ bytes). The SCI approach directly alleviates this by reducing the matrix dimension. The modal integral table is compact ($M \times (N_q{+}1)^2 \times 6$ entries). The sparse pair list and inverted index use integer arrays whose size is data-dependent but typically modest compared to the Hamiltonian itself.

14 Summary and Outlook

⟨Ψ| V_i**Bra** implements a complete pipeline for anharmonic vibrational spectroscopy:

Key features of ⟨Ψ| V_iBra

- Quartic force field from ORCA `.vpt2` files with automatic symmetrisation and degeneracy factors.
- VSCF with state-specific convergence for ground and fundamental states.
- Full VCI with precomputed modal integrals, sparse pair lists, inverted potential indices, prefix–suffix products, and nonzero flags.
- Symmetry-adapted VCI (VCI-SYM): exact block-diagonalisation by molecular point group for the eight Abelian groups with exclusively 1D irreps (C_1, C_s, C_i, C_2, C_{2h}, C_{2v}, D_2, D_{2h}); irrep labels on all eigenstates; factor of n_Γ^2 reduction in diagonalisation cost with no approximation. Point group set via `PGROUP`; atom displacement tolerance set via `PROJCT`.
- Selected VCI (SCI) with CISD reference and Epstein–Nesbet PT2 top-N selection; maximum iterations controlled by `MAXSCI`.
- First-order dipole for HO/VSCF; first and second-order dipole for VCI/SCI/VCI-SYM.
- OpenMP parallelism with tuned scheduling, BLAS/LAPACK.
- Three output files bridging computation and visualisation.
- Python GUI (CustomTkinter) for input and execution management.
- Interactive spectral viewer (Matplotlib) with Boltzmann weighting, JCAMP-DX overlay, ALS baseline correction, per-method controls.
- 3D normal-mode animation via 3Dmol.js.
- VCI wavefunction analysis with transition detail panel and mode-specific animation.

References

[1] J. K. G. Watson, "Simplification of the molecular vibration-rotation Hamiltonian," *Mol. Phys.* **15**, 479–490 (1968).
doi:10.1080/00268976800101381

[2] J. M. Bowman, "Self-consistent field energies and wavefunctions for coupled oscillators," *J. Chem. Phys.* **68**, 608–610 (1978).
doi:10.1063/1.435782

[3] R. B. Gerber and M. A. Ratner, "A semiclassical self-consistent field (SC SCF) approximation for eigenvalues of coupled-vibration systems," *Chem. Phys. Lett.* **68**, 195–198 (1979).
doi:10.1016/0009-2614(79)80099-8

[4] O. Christiansen, "Vibrational coupled cluster theory," *J. Chem. Phys.* **120**, 2149–2159 (2004).
doi:10.1063/1.1637579

[5] J. M. Bowman, T. Carrington, and H.-D. Meyer, "Variational quantum approaches for computing vibrational energies of polyatomic molecules," *Mol. Phys.* **106**, 2145–2182 (2008).
doi:10.1080/00268970802258609

[6] V. Barone, "Anharmonic vibrational properties by a fully automated second-order perturbative approach," *J. Chem. Phys.* **122**, 014108 (2005).
doi:10.1063/1.1824881

[7] O. Christiansen, "Vibrational structure theory: new vibrational wave function methods for calculation of anharmonic vibrational energies and vibrational contributions to molecular properties," *Phys. Chem. Chem. Phys.* **9**, 2942–2953 (2007).
doi:10.1039/b618764a

[8] M. Neff and G. Rauhut, "Toward large scale vibrational configuration interaction calculations," *J. Chem. Phys.* **131**, 124129 (2009).
doi:10.1063/1.3243862

[9] P. S. Epstein, "The Stark effect from the point of view of Schrödinger's quantum theory," *Phys. Rev.* **28**, 695–710 (1926).
doi:10.1103/PhysRev.28.695

[10] R. K. Nesbet, "Configuration interaction in orbital theories," *Proc. R. Soc. Lond. A* **230**, 312–321 (1955).
doi:10.1098/rspa.1955.0134

[11] F. Neese, "Software update: The ORCA program system, version 5.0," *WIREs Comput. Mol. Sci.* **12**, e1606 (2022).
doi:10.1002/wcms.1606
